# Safety and Reactogenicity of an MSP-1 Malaria Vaccine Candidate: A Randomized Phase Ib Dose-Escalation Trial in Kenyan Children

**DOI:** 10.1371/journal.pctr.0010032

**Published:** 2006-11-24

**Authors:** Mark R Withers, Denise McKinney, Bernhards R Ogutu, John N Waitumbi, Jessica B Milman, Odika J Apollo, Otieno G Allen, Kathryn Tucker, Lorraine A Soisson, Carter Diggs, Amanda Leach, Janet Wittes, Filip Dubovsky, V. Ann Stewart, Shon A Remich, Joe Cohen, W. Ripley Ballou, Carolyn A Holland, Jeffrey A Lyon, Evelina Angov, José A Stoute, Samuel K Martin, D. Gray Heppner, for the MSP-1 Malaria Vaccine Working Group

**Affiliations:** 1 United States Army Medical Research Unit–Kenya, Nairobi, Kenya; 2 Kenya Medical Research Institute, Nairobi, Kenya; 3 The Bill and Melinda Gates Foundation, Seattle, Washington, United States of America; 4 Statistics Collaborative, Washington, District of Columbia, United States of America; 5 Malaria Vaccine Development Program, United States Agency for International Development, Washington, District of Columbia, United States of America; 6 GlaxoSmithKline Biologicals, Rixensart, Belgium; 7 MedImmune, Gaithersburg, Maryland, United States of America; 8 Division of Communicable Diseases and Immunology, Walter Reed Army Institute of Research, Silver Spring, Maryland, United States of America

## Abstract

**Objective:**

Our aim was to evaluate the safety, reactogenicity, and immunogenicity of an investigational malaria vaccine.

**Design:**

This was an age-stratified phase Ib, double-blind, randomized, controlled, dose-escalation trial. Children were recruited into one of three cohorts (dosage groups) and randomized in 2:1 fashion to receive either the test product or a comparator.

**Setting:**

The study was conducted in a rural population in Kombewa Division, western Kenya.

**Participants:**

Subjects were 135 children, aged 12–47 mo.

**Interventions:**

Subjects received 10, 25, or 50 μg of falciparum malaria protein 1 (FMP1) formulated in 100, 250, and 500 μL, respectively, of AS02A, or they received a comparator (*Imovax*® rabies vaccine).

**Outcome Measures:**

We performed safety and reactogenicity parameters and assessment of adverse events during solicited (7 d) and unsolicited (30 d) periods after each vaccination. Serious adverse events were monitored for 6 mo after the last vaccination.

**Results:**

Both vaccines were safe and well tolerated. FMP1/AS02A recipients experienced significantly more pain and injection-site swelling with a dose-effect relationship. Systemic reactogenicity was low at all dose levels. Hemoglobin levels remained stable and similar across arms. Baseline geometric mean titers were comparable in all groups. Anti-FMP1 antibody titers increased in a dose-dependent manner in subjects receiving FMP1/AS02A; no increase in anti-FMP1 titers occurred in subjects who received the comparator. By study end, subjects who received either 25 or 50 μg of FMP1 had similar antibody levels, which remained significantly higher than that of those who received the comparator or 10 μg of FMP1. A longitudinal mixed effects model showed a statistically significant effect of dosage level on immune response (F_3,1047_ = 10.78, or F_3, 995_ = 11.22, *p* < 0.001); however, the comparison of 25 μg and 50 μg recipients indicated no significant difference (F_1,1047_ = 0.05; *p* = 0.82).

**Conclusions:**

The FMP1/AS02A vaccine was safe and immunogenic in malaria-exposed 12- to 47-mo-old children and the magnitude of immune response of the 25 and 50 μg doses was superior to that of the 10 μg dose.

## Editorial Commentary

**Background:** Malaria is thought to kill between 1 and 2 million people each year in sub-Saharan Africa; most of these are young children under the age of five, who are particularly prone to developing clinical malaria because their immunity is not yet developed. Many groups of researchers around the world are developing candidate vaccines of different types that it is hoped would protect against malaria. One of these types is a “blood-stage” vaccine, which would prevent parasite multiplication in red blood cells. A candidate blood-stage vaccine is FMP1/AS02A, which is designed to raise an immune response against a particular protein (merozoite surface protein-1) on the surface of the blood-stage infectious form of the malaria parasite. In early-stage clinical trials performed in people not exposed to malaria (healthy volunteers in the United States) and in African adults who were exposed to malaria, this candidate vaccine has already been shown to be safe and to bring about an immune response. As part of the next stage in developing this vaccine, a group of researchers next wanted to see whether the vaccine was also safe and brought about an immune response in the population most in need of a vaccine: young children living in an African region with very intense malaria transmission. Therefore, as reported here, this group performed a small trial in western Kenya, recruiting 135 children under 5 y of age to receive either the FMP1/AS02A vaccine (at three different doses) or rabies vaccine for comparison (thus ensuring that children in the control arm got some benefit from being in the trial). The outcomes that the researchers were interested in were primarily adverse events, which they categorized using a standard questionnaire at up to 7 d after vaccination; unsolicited events reported up to 30 d after vaccination; and, finally, any serious events occurring up to 8 mo later. The researchers also examined antibody responses to the FMP1/AS02A vaccine.

**What this trial shows:** Participants who received the FMP1/AS02A vaccine (as compared to the rabies vaccine) experienced more immediate symptoms, such as pain and swelling at the injection site. Most participants reported unsolicited events during follow-up, but the proportion of participants with adverse events did not seem to be different between the FMP1/AS02A vaccine groups and the rabies vaccine group. Unsolicited outcomes that were reported included, for example, clinical malaria, upper respiratory tract infections, and a few events that were thought to be related to the vaccines, such as fever and eczema. A few serious adverse events occurred up to 8 mo after vaccination, but the numbers did not seem to be different between the FMP1/AS02A and rabies vaccine groups, and the events were not judged to be related to vaccination. Finally, participants who received the FMP1/AS02A vaccine raised an antibody response to the vaccine, which was highest in those who received the highest vaccine dose. The researchers concluded that this vaccine was safe and brought about an immune response in the group of malaria-exposed children studied.

**Strengths and limitations:** The trial was conducted in a population that is likely to benefit from the vaccine, if it is shown to be effective in further studies. Therefore, the data obtained from this study will be informative in helping to design future trials on FMP1/AS02A. The randomization procedures used in this study were appropriate, and in particular participants of different ages were equally distributed to the different intervention groups, helping to minimize bias. Procedures were also set up to prevent participants and staff giving the vaccines and collecting data from knowing which interventions participants had received. However, the number of participants recruited into the trial was small, and it therefore was not powered to detect anything other than large differences in rates of adverse events between the study groups.

**Contribution to the evidence:** This study extends evidence from prior trials on the safety and immunogenicity of the FMP1/AS02A vaccine to a population that is representative of those most in need of an effective vaccine—young African children. The results suggest that the vaccine candidate should undergo further evaluation in trials examining vaccine efficacy in a similar population.

## INTRODUCTION

The world struggles to come to terms with the immensity of the public health, economic, and political consequences of malaria morbidity and mortality. Although no inhabited continent is unaffected, Africa is particularly hard hit. Annually, over half a billion Plasmodium falciparum infections occur worldwide, leading to between 1 and 2 million deaths in sub-Saharan Africans—most of them children [1–3]. The proportion of total mortality attributable to malaria in this pediatric population has increased in recent years because of a general breakdown of public health services in many areas, antimalarial drug resistance, interactions with the human immunodeficiency virus, and (perhaps) global climate change [4]. Nearly 100 candidate malaria vaccines are currently in various stages of evaluation [5], and a pre-erythrocytic (pre-blood stage, liver-stage) vaccine candidate, known as RTS,S, has recently demonstrated efficacy against clinical and severe disease in children in Mozambique [6,7]. An alternative to such pre-erythrocytic vaccines is the targeting of antigens that are expressed during the erythrocytic (blood) stage of the parasite life cycle. The rationale for such an erythrocytic stage vaccine is based on the observation that natural protection against clinical disease in adults and in young children depends upon maintenance of high antibody levels to antigens present on the form of the parasite (the merozoite) that circulates in the bloodstream [8,9]. The P. falciparum merozoite surface protein 1 (MSP-1) is a 195-kDa protein that is proteolytically cleaved to yield four fragments, which are associated with each other through noncovalent interactions on the merozoite surface. Among them is the carboxy-terminal 42-kDa fragment known as MSP-1_42_ [10,11]. Secondary processing of the 42-kDa fragment into 19-kDa and 33-kDa fragments is thought important for merozoite invasion [12]. Individuals living under high malaria transmission develop anti-MSP-1_42_ and anti-MSP-1_19_ antibodies that inhibit parasite growth in vitro [13]. Moreover, inoculation of Aotus monkeys with a recombinant MSP-1_42_ and potent adjuvant can confer protection against blood-stage challenge with P. falciparum [14–16]. In human subjects, the candidacy of MSP-1_42_ as a potentially efficacious malaria vaccine is further supported by epidemiologic studies demonstrating that antibodies to the relatively conserved domains are associated with a diminution of P. falciparum disease severity [9,17] and by the finding that the majority of antibodies active in growth inhibition in sera from endemic areas are p19 specific [18].

The MSP-1_42_ of the 3D7 clone of P. falciparum has been formulated into a final test product, termed the falciparum malaria protein 1 (FMP1) [19], and combined with GlaxoSmithKline's proprietary adjuvant AS02A [20]. The safety and immunogenicity of the FMP1/AS02A formulation has been confirmed in two phase I trials conducted in the United States [21] and in phase I trials in malaria-experienced populations in western Kenya [22] and Mali (C. Plowe, personal communication). The present study, a phase Ib trial in young children subject to intense malaria transmission, follows up the previous adult trial in the same area of western Kenya. It is part of our long-term malaria vaccine development plan that aims to develop products to prevent malaria morbidity and mortality in infants and young children in malaria endemic areas.

## METHODS

### Participants

The trial was conducted in Kombewa Division in the Kisumu District of Nyanza Province of western Kenya. The study subjects were drawn almost exclusively from the Luo ethnic group living in low-land country near Lake Victoria. The Luo are settled agriculturalists of Nilo-Saharan origin, most of whom engage in low-income subsistence farming or fishing. In this region of holoendemic malaria transmission, most infections (90%) are transmitted by mosquitoes of the Anopheles gambiae complex and the infecting parasite species is usually (95%) P. falciparum [23,24]. Transmission is intense, with documented parasite prevalence during disease seasons reaching as high as 83% among 1- to 4-y-old children [25] and monthly clinical attack rates in the same ages as high as 54% (unpublished data).

Data from a previous comprehensive census of Kombewa Division, as well as from a more focused census done immediately prior to recruitment, were used to identify potential field stations. This fairly comprehensive demographic database established how many eligibles (on the basis of age) were present in the areas to be drawn from. All parents known to have children in the relevant age groups and living within a 1-mile (1.6-km) radius of each of 12 field stations were invited to participate in a briefing.

Prior to enrollment in this study, a medical history, physical examination, and screening laboratory tests were performed on every subject to detect any underlying medical condition that might confound clinical evaluation or data analysis. Males and females were considered eligible for enrollment if they were 12–47 mo old at the time of screening and in good health and if a parent agreed to sign a written and witnessed informed consent form and to remain in the study area for 12 mo. Exclusion criteria included any evidence of acute or chronic medical conditions that might increase the risk to the participating subject. Specific exclusion criteria included prior receipt of an investigational malaria vaccine or of a rabies vaccine; recent or planned use of any investigational new drug (IND), vaccine, immunoglobulin, or any blood product; use of immunosuppressant drugs within the previous 6 mo; confirmed or suspected immunodeficiency; history of splenectomy; administration, or anticipated administration, of a vaccine not foreseen by the study protocol within 30 d of the first dose of vaccine (with the exception of tetanus toxoid); and concurrent participation in another clinical trial. Laboratory exclusion criteria were findings of serum alanine aminotransferase (ALT) of **>**45 IU/L; serum creatinine of **>** 1.1 mg/dL; absolute lymphocyte count (ALC) for 1-y-olds of < 4.0 × 10^3^/mm^3^, for 2-y-olds of < 3.0 × 10^3^/mm^3^, and for 3-y-olds of < 2.0 × 10^3^/mm^3^; platelet count of <100,000/mm^3^; hemoglobin of < 8 g/dL; homozygosity for sickle cell disease (SS) genotype (by protein gel electrophoresis); and malnutrition defined as weight for height of **<** −3 *z*-scores.

Participants were recruited under a human use protocol approved by and executed in accordance with the guidelines of the Office of the Surgeon General, United States Army; the Ethics Review Committee of the Kenya Medical Research Institute (Kenya Ministry of Health); and the Human Subjects Protection Committee of the Program for Appropriate Technology in Health (PATH). Informed consent was obtained from all participants in accordance with all applicable guidelines.

### Interventions

The expression, purification, biochemical, and immunological characterization of the Escherichia coli–produced, GMP-manufactured, FMP1 antigen have been described elsewhere [19], as has the manufacture and packaging of AS02A [22]. Immediately prior to immunization, the contents of one syringe prefilled with AS02A (approximately 0.6 mL) were injected into a vial of lyophilized FMP1 antigen (approximately 64 μg). The pellet of FMP1 was then dissolved by gently swirling the vial to ensure complete dissolution of the contents (yielding a milky white fluid) to yield final delivered volumes of 0.1, 0.25, or 0.5 mL (for doses of 10, 25, or 50 μg, respectively).

The comparator vaccine, an inactivated rabies vaccine (*Imovax*® Rabies, produced by Aventis Pasteur, Lyon, France), has been described elsewhere [22]. Immediately prior to immunization, the complete contents of a prefilled syringe containing diluent (1 mL of sterile water for injection) were injected into a vial of lyophilized vaccine, and the pellet was allowed to dissolve by gently swirling the vial to ensure complete dissolution before withdrawing 1.0 mL of the reconstituted rabies vaccine, a clear pink liquid.

Subjects were vaccinated by intramuscular injection alternately into right or left anterolateral thigh muscles according to a 0-, 1-, and 2-mo schedule. The trial had three immunization cohorts for purposes of dose escalation. Each cohort contained 30 subjects that received 10, 25, or 50 μg of FMP1/AS02A and 15 subjects that received standard doses of the rabies vaccine comparator. Each dosage cohort was age stratified to ensure that any imbalance in safety and reactogenicity rates was not due to a disproportion of young children in any one cohort (Table 1).

**Table 1:**
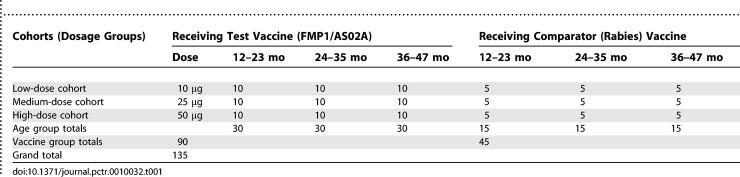
Numbers of Subjects by Cohort (Dosage Group), Study Arm (Vaccine Group), and Age Group

An independent data and safety monitoring board (DSMB) was appointed before the study began to review both the safety data reports as the trial progressed and the data analysis by Statistics Collaborative after study completion. DSMB membership included a statistician and four senior clinical research investigators with experience in conducting malaria vaccine trials, one of whom was a Kenyan. An experienced local clinician (Ambrose Misore, M.B.Ch.B.) served as the local medical monitor (LMM), reviewed all serious adverse events (SAEs) and safety data between dose escalations, and functioned as the patient advocate. In addition, the trial was monitored for regulatory compliance by representatives of the United States Army Medical Materiel Development Activity, GlaxoSmithKline Biologicals, and Pharmaceuticals Product Development (a contract research organization based in Wilmington, North Carolina, United States), all of whom made several visits to the study site. Initial approval of the study protocol and of subsequent protocol amendments was granted to the investigators by the Ethical Review Committee of the Kenya Medical Research Institute, Nairobi; by the Human Subjects Protection Committee of PATH, Seattle, Washington, United States; and by the United States Army HSRRB. The study was done under a Food and Drug Administration IND and was compliant with all relevant International Conference on Harmonization guidelines.

Vaccinations of the second and third cohorts were staggered from each other by 2 wk (Table 2). A safety report was produced prior to each dose escalation. The LMM and the DSMB reviewed all adverse events (AEs) occurring in the 7 d immediately following any vaccination that preceded a dose escalation. Written approval from the LMM and concurrence by the DSMB were required prior to any subsequent dose escalation.

**Table 2:**
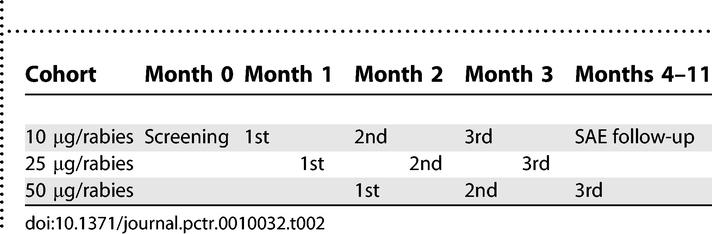
Timing of First, Second, and Third Vaccinations for Each Cohort (Dosage Group) over 12 wk

Stopping rules were to be invoked when either of the following observations was made: (1) > 20% of subjects with “severe” (grade 3) general AEs (not local AEs) related to the vaccine; or (2) any SAE, including death, judged to be vaccine related.

### Objectives

The primary objective was to assess the safety and reactogenicity of the FMP1/AS02A malaria vaccine in malaria-exposed 12- to 47-mo-old children living in western Kenya. The secondary objective was to assess the humoral immune response to the FMP1/AS02A malaria vaccine in malaria-exposed 12- to 47-mo-old children living in western Kenya.

### Outcomes

The primary endpoints were (1) occurrence of solicited symptoms (based on a standardized questionnaire) during a 7-d follow-up period after each vaccination (postvaccination clinic visits occurred on study days 1, 2, 3, and 7 after each vaccination); (2) occurrence of unsolicited symptoms during a 30-d follow-up period after each vaccination; and (3) occurrence of SAEs during an 8-mo follow-up period following the first dose of study vaccine (i.e., 6 mo following the last vaccination). The secondary endpoints were anti-FMP1 (anti-MSP-1_42_ 3D7 strain antibody) titers as determined by an enzyme-linked immunosorbent assay (ELISA) on study days 0, 14, 30, 44, 60, 74, 90, 180, 270, and 364.

#### Assessment of primary endpoints (safety and reactogenicity).

Following each vaccination, subjects were followed for occurrence of solicited symptoms for 7 d, unsolicited symptoms for 30 d, and SAEs for 8 mo (i.e., 6 mo after the last vaccination) or until resolution. Both local (injection-site pain and swelling) and general/systemic (fever, drowsiness, loss of appetite, and irritability/fussiness) symptoms were assessed (Table 3). After the final vaccination, subjects were followed monthly at home by field workers and were asked to return to the clinic every 3 mo until the end of the study for safety follow-up. An SAE was defined as any untoward medical occurrence that resulted in death, was life threatening, resulted in persistent or significant disability or incapacity, or required in-patient hospitalization (or prolongation of hospitalization). Important medical events that might jeopardize a subject or might require intervention to prevent one of the other outcomes listed above were considered SAEs. Serum creatinine, ALT, white blood cell count, lymphocyte count, platelet count, and hemoglobin were determined on study days 0, 14, 30, 44, 60, 74, and 90. Additional hemoglobin determinations were made on study days 180, 270, and 364. Normal ranges were calculated on the basis of previous data from the local pediatric population.

**Table 3:**
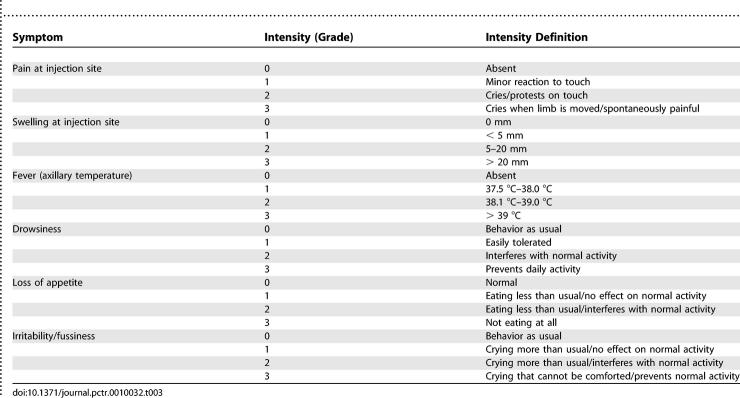
Assessment of Symptom Intensities

#### Assessment of malaria.

A peripheral blood smear was obtained from any subject who presented to the Walter Reed Project's Kombewa Clinic with fever, a history of fever within 48 h, or an illness that the attending doctor suspected might be due to malaria infection. After Giemsa staining and examination by oil-immersion light microscopy, detection of asexual parasitemia of > 0 parasites/μL resulted in the diagnosis and treatment for malaria.

#### Assessment of secondary endpoints (humoral responses).

Immunology samples were collected on study days 0, 14, 30, 44, 60, 74, 90, 180, 270, and 364; samples collected on study days 0, 30, and 60 were collected immediately prior to vaccination. Immune response to the FMP1/AS02A vaccine was determined by anti-FMP1 ELISA endpoint titers reported in optical density units (ODUs), the dilution yielding an ODU of 1.0 in our assay. This assay has been described in detail elsewhere [22].

### Sample Size

This trial represents the first time to our knowledge that this vaccine candidate has been evaluated in a pediatric population. Sample size was chosen after weighing the need to detect any possible vaccine associated AEs against the need to limit the number of subjects exposed to an investigational product. Incorporation of the *Imovax*® Rabies vaccine comparison group enabled broad initial estimates of the incidence of local and general side effects in a population that suffers from significant comorbidity from exposure to endemic illnesses. The control cohort also served as a comparator for the longitudinal immune responses to the FMP1 antigen in a malaria-exposed population. Although comparative statistics for the safety variables were calculated, the study had low power to detect anything other than large differences in the incidence of local and general side effects between the vaccination groups.

With sample sizes of 15, 30, and 45 (corresponding to the sizes of groups within the trial), we have 90% power to detect an AE that occurs in 14%, 7%, and 5% of the population, respectively. The study was not powered to detect an immune response because the primary focus of this trial was safety.

### Randomization: Sequence Generation

A randomization list generated by Statistics Collaborative contained sequential codes linked to a study vaccine assignment. These codes were assigned to subjects in the order in which they presented to the clinic on the day of first vaccination. Blocked blinded randomization was used with stratification for age and dosage groups. Fifteen subjects from each age group were randomized into each of the three cohorts (10 receiving the test article and five receiving the comparator). Because of the very small sample sizes (Table 1), these strata were not intended for analysis.

### Randomization: Allocation Concealment

The only persons at the study site with access to the randomization assignments were the study drug manager, the clinic pharmacist, and his assistant; it was necessary that these individuals have access to the codes for preparation of test articles. Each randomization assignment was sealed in a unique, tamper-evident envelope, which was opened at the time a subject presented for the first vaccination. The LMM also kept one set of the randomization codes in a sealed envelope in the event that emergency unblinding became necessary.

### Randomization: Implementation

Subjects were randomized in the order in which they presented on the first day of vaccination in a 2:1 ratio to receive either FMP1/AS02A (90 subjects) or the comparator vaccine (45 subjects). The subjects were enrolled by the study drug manager, clinic pharmacist, and his assistant, and these individuals assigned the subjects to their groups.

### Blinding

Because the color and volumes of the reconstituted FMP1/AS02A and comparator vaccines differed, the barrel of the syringe was covered with opaque tape to mask its contents and labeled with the subject identification number and randomization code. Subjects, parents, and the staff performing follow-up evaluations were all blinded. Immunizations were carried out simultaneously in four separate consultation rooms that were connected to a central pharmacy (the vaccine preparation room) by small, closable service hatches. On vaccination days, the prepared syringe was handed through a service hatch to a vaccinator for vaccine administration. For each subject, an identification number, a randomization code from a chart, and a randomization code on the syringe were recorded on a vaccination form. Following vaccine administration, subjects were assessed and follow-up visits conducted by a group of clinicians who had not been involved in the vaccinations. The study investigators became unblinded to treatment allocations in July 2005, after study completion (which was September 2004).

### Statistical Methods

Statistical analyses were performed by Statistics Collaborative using SAS version 8.2 (SAS Institute, Cary, North Carolina, United States). The incidence of solicited, unsolicited, and serious AEs were compared using two-sided Fisher's exact tests without correction for multiplicity. Geometric mean titers (GMTs) were calculated to assess immunogenicity at each timepoint when titers were collected. The titer data were transformed to a log_10_ scale and were modeled by longitudinal mixed models to assess the effect of dose and age on the mean level of the antibody responses over time. The models used a spatial covariance structure, which takes into account the number of study days between two measurements when determining the correlation between them.

## RESULTS

### Participant Flow

**Figure 1:**
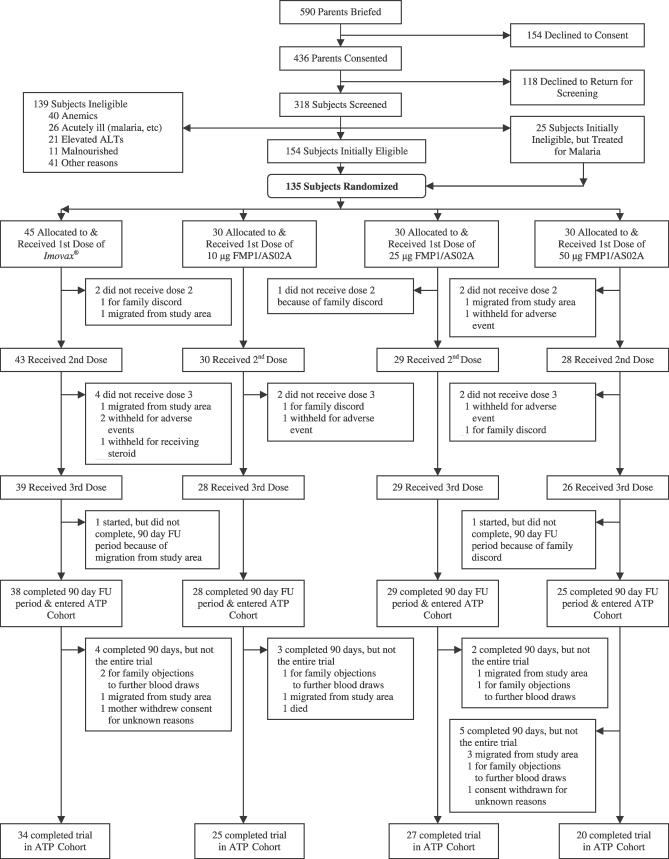
Trial Profile

A total of 590 parents of children were briefed; of these, 436 parents consented to screening. Of the 320 children who were returned for screening, 135 (77 girls and 58 boys) were enrolled and randomized to one of the three dosage cohorts. Among these were 25 children who were initially disqualified owing to clinical malaria; however, they were subsequently enrolled after successful treatment and confirmed cure. Of the 135 subjects who received the first vaccination, five did not receive the second vaccination and another eight did not receive the third. Thus, 122 subjects received all three vaccinations; 83 of the 90 subjects were randomized to receive FMP1/AS02A and 39 of the 45 randomized to receive the rabies vaccine. The 13 incompletely vaccinated subjects were evenly distributed among the three dose cohorts (Figure 1). Because all of the 135 enrolled subjects received at least one vaccination, all were to be followed per protocol for the study duration and included in the safety analyses. Twenty-five subjects withdrew prematurely from the study, approximately 20% from each study arm; these withdrawals were also evenly distributed across cohorts. Twelve subjects had consent withdrawn by a parent or grandparent because of family discord regarding participation in the study; 11 subjects migrated out of the study area; one subject attended the final study visit during which the mother refused the blood draw; and one subject died. With the exception of the last, no subject was withdrawn from the study (as opposed to withheld from further vaccinations) because of an AE.

### Recruitment

Recruiting and enrollment occurred from 25 July through 12 September 2003. The study duration was approximately 12 mo for a subject with the last study visit on 19 September 2004.

### Baseline Data

The study groups were comparable in baseline demography, height, weight, and vital signs (temperature, pulse, respiratory rate, and blood pressure). Baseline clinical laboratory measurements other than ALC were consistent across study arms; most measurements fell within the local normal range. Both study arms had a number of subjects outside of the normal range for ALC at the sampled timepoints, including prior to receipt of the first vaccination, but none of these was deemed clinically significant. Age, sex, height, weight, clinical laboratory values, and antibody to FMP1 prior to the first vaccination are presented in Table 4 for the four study arms.

**Table 4:**
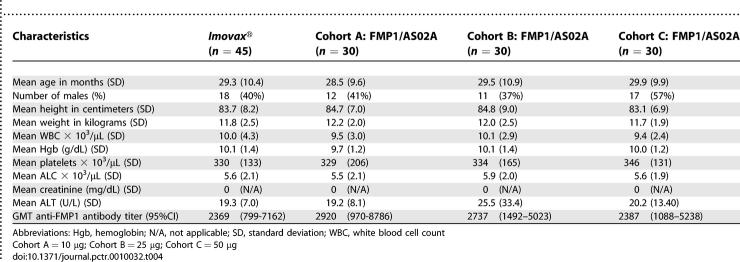
Demographic, Clinical, and Laboratory Baseline Characteristics of 135 Enrolled Subjects Prior to First Vaccinations

### Numbers Analyzed

This study randomized 135 children (aged 12–47 mo) into three cohorts of 45 subjects, each consisting of 30 children who received FMP1/AS02A (10, 25, or 50 μg of FMP1 in 0.1, 0.25, or 0.50 mL of AS02A, respectively) and 15 children who received the comparator vaccine. Each cohort contained 15 subjects, 10 receiving FMP1/AS02 and five the comparator, in each of three age groups (12–23, 24–35, and 36–47 mo, or 1-, 2-, and 3-y-olds) for a total of 45 subjects in each age group distributed among the three cohorts. The comparator groups received rabies vaccine (Table 1). Each subject was to be studied for approximately 12 mo. Safety analyses were performed on an intention-to-treat cohort; immunogenicity analyses were performed on an according-to-protocol cohort that received all three vaccinations (106 subjects) (Figure 1).

### Outcomes and Estimation of Safety and Reactogenicity

#### Solicited symptoms.

Both the test article and comparator vaccines were well tolerated. No parent or child withdrew from the study for a vaccine-related side effect. Table 5 summarizes the solicited signs and symptoms during the 7-d follow-up periods after vaccinations. Both local symptoms (pain and swelling) were defined as vaccine-related AEs. Subjects in all cohorts who received FMP1/AS02A experienced more local symptoms than those who received the comparator, and a dose-related response was apparent. The largest percentage of these subjects experienced a local reaction immediately following the first vaccination (46% of FMP1/AS02A subjects versus 2% of comparator subjects; *p*-value, < 0.001); the percentages of subjects experiencing local symptoms during second and third vaccinations were lower in both study arms (respectively, 40% versus 9%; *p*-value, < 0.001; and 37% versus 0%; *p*-value, < 0.001). The most common local reaction at any time was pain at the site of injection. Up to 38% of subjects receiving FMP1/AS02A (percentages varied by dosage group) also experienced injection-site swelling; however, no subject receiving the comparator experienced swelling.

**Table 5:**
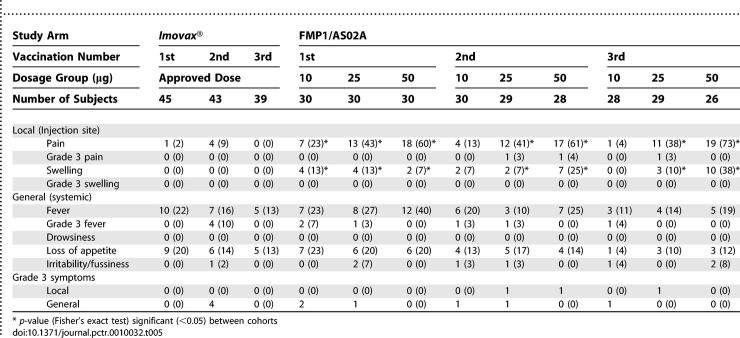
Number (Percentage) of Subjects Experiencing Solicited Signs and Symptoms during the 7-d Follow-Up after Each Vaccination by Study Arm

No sequence- or dose-related trends were apparent for general solicited symptoms (Table 5). The most common general symptoms in both study arms were fever and loss of appetite. No instance of drowsiness occurred during any solicited symptom follow-up period. Fever was seen after vaccination in all dosage groups, with the highest rates being seen in the 50-μg dosage group. Loss of appetite and irritability/fussiness were seen at similar rates in all dosage groups and the comparator vaccine.

Few subjects experienced grade 3 symptoms (Table 5). Grade 3 pain at the injection site was seen sporadically with the second and third immunization in the 25- and 50-μg dosage groups of FMP1/AS02A. Grade 3 fever was seen sporadically in both the test article and the comparator vaccine.

#### Unsolicited symptoms.

Unsolicited symptoms were recorded for 30 d following each vaccine administration and were categorized by a modified World Health Organization Adverse Reactions Terminology (WHOART) AE coding system. There were no differences by group or by cohort in the proportions of subjects experiencing an unsolicited symptom. All but one of the enrolled children experienced at least one unsolicited symptom during a follow-up period, and most experienced an unsolicited symptom during each of the three follow-up periods (77%–97% for FMP1/AS02A and 80%–100% for the comparator). The most common unsolicited symptoms were upper respiratory tract infections (URTIs) and malaria.

Approximately 80% of subjects in each study arm experienced at least one URTI during the three 30-d postimmunization follow-up periods. There was no indication that vaccinated groups had any increased risk of developing clinical malaria.

For unsolicited symptoms, vaccine relatedness was determined by temporal relationship to a vaccination with absence of any reasonably explanatory comorbidity. Very few vaccine-related unsolicited symptoms occurred during the postimmunization follow-up period (five and one among FMP1/AS02A and comparator recipients, respectively). These symptoms were mostly limited to injection-site reactions that differed from those elicited during the immediate follow-up period. They included a case of hyperpigmentation in the comparator group and three cases of fever by history, one of induration, and one of eczema in the FMP1/AS02A group. The last mentioned was a nonpruritic skin rash developing in the right flank area about 50 min after a second vaccination of the 10-μg dose of FMP1/AS02A. Initially presenting with a plaque-like appearance, these lesions developed a follicular eczematous pattern that then resolved over a few days. This child had been noted to have similar (albeit smaller) dermatitic or eczematous lesions prior to the first vaccination. A third vaccination was withheld from this subject. One grade 3 unsolicited symptom occurred during the postimmunization follow-up period, a 25-μg FMP1/AS02A subject experiencing grade 3 malaria beginning 2 d after receiving the first vaccination. (Grade 3 was defined as an event severe enough to prevent normal daily activities.) No subject in the study experienced a grade 3 vaccine-related, unsolicited symptom.

#### Serious and unexpected AEs.

Serious and unexpected AEs were collected on a real-time basis through study day 240. No SAE was judged causally related to a vaccination. Seven documented SAEs occurred during this period: four in the FMP1/AS02A group and three in the comparator group. In the former group, two children in the 10-μg dosage cohort experienced convulsions 17 and 189 d after second and third vaccinations, respectively; one child in the 50-μg dosage group experienced convulsions 12 d after the third vaccination. (All convulsions were febrile, malaria-associated seizures.)

A 37-mo-old female acquired malaria 26 d after receiving a third dose of 10 μg of FMP1/AS02A, was treated, and recovered. She developed hepatitis and severe anemia 7 d later. She died 62 d after her third vaccination, following several hospitalizations and blood transfusions. In this subject, an extravascular hemolysis caused severe organomegaly with secondary thrombocytopenia and hypersplenism, along with a possible partial obstruction of biliary outflow. The death of this subject led to a thorough review of the case by the principal investigator, the DSMB, and a pediatric hematology consultant not previously associated with the study. After considering all potential etiologies (including Epstein–Barr virus, parvovirus B19, and others) that could have precipitated such a clinical course and the clinical context (e.g., the long interval between vaccination and onset), it was concluded that there was not a likely causal biological connection between the immunization and the preterminal course and death. In accordance with the study protocol, the principal investigator's judgment was that “there are other, more likely causes and administration of the study vaccine is not suspected to have contributed to the adverse event.” Her death was judged not related to the study vaccine, but rather to autoimmune hemolytic anemia, most likely secondary to a viral infection, causing first hepatitis and then an unusual warm reactive immunoglobulin G. The clinical course and the temporal association are here reported so that in the event of similar instances, this information will be readily available.

In the comparator group, one subject experienced febrile, malaria-associated convulsions; one, an episode of severe bronchospasm; and one, an intestinal obstruction due to ascariasis. One unexpected AE occurred during the study: a subject from the comparator group experienced a markedly elevated ALT level and was diagnosed with hepatitis A. The subject was excluded from the third vaccination to eliminate the possibility of confounding the assessment of safety following subsequent vaccinations. In addition, two subjects from the FMP1/AS02A group developed phimosis, leading to elective hospitalizations and circumcisions.

#### Laboratory parameters.

Blood (venous samples from arms) for laboratory measurements (white blood cell count, hemoglobin, platelet count, ALC, creatinine, and ALT) was collected at baseline (study day 0) and on study days 14, 30, 44, 60, 74, and 90. Group mean safety laboratory values were generally unchanged in the 30-d postimmunization follow-up periods. The majority of subjects had all values within normal range. However, both vaccine and comparator groups had a number of subjects outside of the normal range of ALCs, including timepoints prior to first vaccination; none was judged to be clinically significant.

The protocol specified that each child was to receive 10 hemoglobin determinations occurring at the seven timepoints cited above and on study days 180, 270, and 364. None of the 90 children who received FMP1/AS02A had a hemoglobin level below the local lower limit of normal (9.75 g/dL ± 2 SD, 6.5–13.0 g/dL); one of the 45 children enrolled in the comparator arm fell below this limit at one timepoint.

### Outcomes and Estimation of Immunogenicity

**Figure 2:**
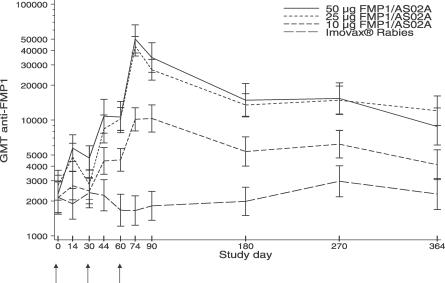
Anti-FMP1 GMTs over Time, by Vaccine Group Arrows indicate vaccination time points.

As expected of a subject population that had been highly exposed to malaria prior to vaccination, no baseline anti-FMP1 titer fell below the limit of detection. Baseline GMTs were comparable across study arms and dosage cohorts, ranging from approximately 1,000 to 3,000 ODUs. The overall baseline GMT in thousands (across all study groups) was 2.3 (95% confidence interval [CI] of 1.7, 3.2). After three vaccinations, no increase in antibody response was observed in subjects who received the comparator; however, antibody response increased with increasing dosage level of FMP1/AS02A (Figure 2). GMT peaked 2 wk after the third immunization (study day 74), at which time the GMTs (in thousands) were 10 (95%CI, 6–17), 43 (95%CI, 29–65), and 58 (95%CI, 38–89) ODU in the 10-μg, 25-μg, and 50-μg groups, respectively. GMT in the comparator group was 1.5 with a 95%CI of 0.8–2.7. The study day 90 GMT (in thousands) for the 10-μg dosage cohort remained constant at 10 (95%CI, 6–18), but the study day 90 GMT for the 25- and 50-μg dosage cohorts dropped to 27 (95%CI, 18–41) and 40 (95%CI, 25–62), respectively. From study day 180, the antibody levels in the 25- and 50-μg dosage groups begin to diminish; however, by study day 364 they still maintained higher antibody levels than the subjects in the 10-μg dosage group and in the comparator group.

**Figure 3:**
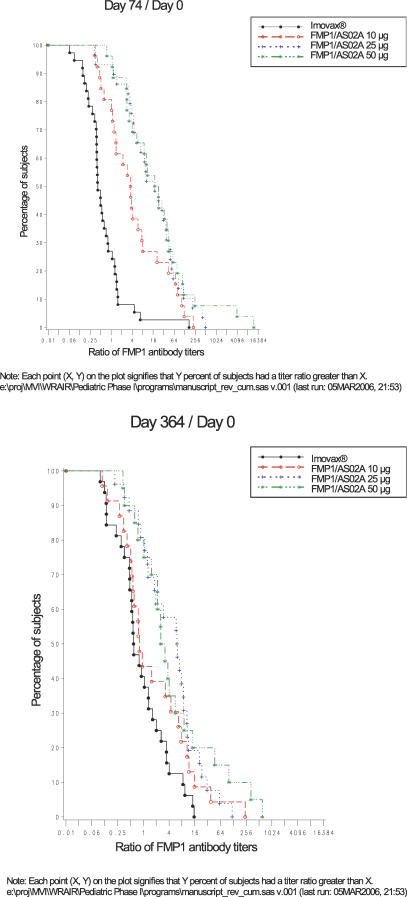
Reverse Cumulative Plot of Anti-FMP1 Titer Ratios, by Dose Cohort, for Subjects Receiving All Three Vaccinations (A) Ratios are depicted comparing study day 0 titers to study day 74. (B) Ratios are depicted comparing study day 0 titers to study day 364.

Titer ratio plots indicate the percentage of subjects experiencing specific fold rises in antibody response over baseline values (Figure 3). At study day 74, the graph indicates that approximately 50% of subjects receiving the 10-μg dose of FMP1/AS02A experienced at least a 4-fold rise, and approximately 50% of subjects receiving the 25- or 50-μg dose experienced at least a 16-fold rise. At study day 364, approximately 50% of subjects in the 25- and 50-μg dose cohorts of the FMP1/AS02A group showed at least a 4-fold rise in antibody titer, and at least 20% of these two cohorts maintained a 16-fold rise.

#### Longitudinal models.

To assess the effect of dose and age group on the mean level of antibody responses over time, we estimated least-square means and standard errors of log_10_-transformed values of anti-FMP1. All subjects were included in the model (data not shown). Tests of main effects (dose and age group), the age group × dosage level interaction, and dose response were performed. The results from the model for all randomized subjects showed the immune response differed among dosage levels (F_3,995_ = 11.22; *p* < 0.001). Neither the age group × dosage level interaction nor the age group main effect is statistically significant (F_6,995_ = 1.02, *p* = 0.41; F_2,995_ = 0.25, *p* = 0.78, respectively), providing no evidence that a subject's age influences the vaccine's immunogenicity over time. A highly significant linear trend in dose response was observed (F_1,995_ = 29.65; *p* < 0.001). The 50-μg dosage group had a higher response than the 10-μg dosage group (F_1,995_ = 6.68; *p* = 0.010); however, the 25- and 50-μg dosage groups did not show a significant difference (F_1,995_ = 0.2; *p* = 0.65).

## DISCUSSION

### Interpretation

This phase Ib dose-escalation and safety trial provided clear evidence of the safety and tolerability of 10-, 25-, and 50-μg doses of FMP1/AS02A when given to young children subject to intense malaria transmission in western Kenya. Although subjects allocated to the test article arm experienced more solicited local symptoms, the proportion affected is comparable to the symptoms seen with another vaccine antigen formulated with the same adjuvant system [6]. The frequency of solicited general symptoms was similar among the groups, and neither vaccine group experienced significant numbers of local or general solicited grade 3 reactions. The unsolicited symptoms experienced were quite similar in type, number, and intensity between the two vaccines, with malaria and URTI predominating. Laboratory parameters were comparable throughout the study. No local or systemic allergic event occurred. The seven SAEs occurring during the 8-mo observation period were evenly divided between test article and comparator arms, with none adjudged causally related to receipt of a vaccine. Further evidence of the general tolerability of FMP1/AS02A in this population is the equivalent dropout rate in the two arms.

The 25- and 50-μg doses generated a humoral immune response that was of greater magnitude than the 10-μg dose in this pediatric population. The previous FMP1/AS02A adult study at Kombewa [22] found a statistically significant antibody response to the same three-dose regimen in a semi-immune adult population that had a substantial baseline anti-FMP1 antibody titer. In that study, titers peaked 90 d after receipt of 50 μg of FMP1/AS02A with an average 2.7-fold increase over baseline levels. This immunogenicity of the vaccine in adults may have been masked by the high levels of preexisting antibody in that semi-immune population; this was supported by the observation that the greatest rise in antibody was seen in subjects with the lowest baseline levels. The present study confirms our anticipation that this vaccine candidate would induce an even greater increase in anti-FMP1 titers when administered to children with less malaria exposure and lower baseline titers.

The waning of antibody levels in the 25- and 50-μg dosage groups after study day 74 is congruent with previous experience with FMP1. Significantly, by study day 364, the 25- and 50-μg dosage groups maintained higher antibody levels than subjects in the 10-μg dosage and comparator groups. It is not clear what effect boosting from natural exposure had on the magnitude and persistence of the antibody response, but the anti-FMP1 response in the rabies comparator group did not significantly increase over the year-long observation period. The results from the log_10_-transformed linear modeling for all randomized subjects showed statistically convincing evidence of a dose-response relationship to antibody response.

Consistent with our ultimate goal of allowing administration of a malaria vaccine as part of the World Health Organization's Expanded Programme on Immunisation (EPI) to infants at greatest risk of malaria, we have here followed up the previous year's trial of FMP1/AS02A in adults in Kombewa [22] with another EPI-compatible 0-, 1-, and 2-mo schedule. This brisk schedule has now been shown to be well tolerated in both trials and was not associated with any new or higher incidence of postvaccination symptoms compared to trials of FMP1/AS02A given on a 0-, 1-, and 3-mo schedule in healthy malaria-naïve adults in the United States [21].

Reports of experimentally induced, malaria-associated anemia in *Aotus* monkeys subjected to prolonged P. falciparum parasitemia have raised concern that malaria vaccines eliciting immunity that controls, but does not eliminate, parasitemia might themselves increase the risk of anemia in endemic human populations [26]. In the previous year's adult study, hemoglobin levels were stable in both test article and comparator groups for 365 d. Results of the present study are similarly reassuring in indicating that hemoglobin levels do not deteriorate in children, even when they are exposed to high levels of malaria transmission after vaccination. Indeed, slight continuous increases in hemoglobin levels occurred in all groups, perhaps because of the enhanced medical care provided to these study subjects.

### Generalizability

The study population selected for this trial was chosen as representative of the target population for a malaria vaccine: at-risk, malaria-experienced children living in an area of endemic, holoendemic, or epidemic malaria. As the primary objective of the trial centered upon evaluation of safety and reactogenicity, results should be broadly generalizable to children of the representative age groups. Results pertaining to the secondary objective (evaluation of immunogenicity) should be generalizable to children of these age groups exposed to P. falciparum infections at levels obtaining in the study area, but may not be generalizable to children under other transmission intensities.

### Overall Evidence

Along with the previous year's adult trial, this trial accomplishes the first two of the three immediate goals of the clinical development plan [27] for FMP1/AS02A, namely, expansion of the safety and immunogenicity profile in endemic populations and dose exploration of the test article in children. The third goal—determination of the preliminary efficacy of this vaccine for reduction of clinical malaria in children at risk of disease—awaits the outcome of a phase IIb efficacy trial. On the basis of preliminary presentations of the results included here, this proposed efficacy trial received the endorsement of the DSMB and the relevant institutional review boards in mid-2004 and is currently underway in Kombewa. Results should be available by the end of 2006.

## SUPPORTING INFORMATION

CONSORT Checklist Click here for additional data file. (255 KB PDF)

Trial Protocol Click here for additional data file. (1.0 MB DOC)

## Abbreviations

AE: adverse event
ALC: absolute lymphocyte count
ALT: alanine aminotransferase
AS02A: adjuvant system 2 of GlaxoSmithKline without thimerosal
CI: confidence interval
DSMB: data and safety monitoring board
ELISA: enzyme-linked immunosorbent assay
FMP1: falciparum malaria protein 1
GMT: geometric mean titer
HSRRB: human subjects research review board
IND: investigational new drug
LMM: local medical monitor
MSP-1: merozoite surface protein 1
ODU: optical density unit
PATH: Program for Appropriate Technology in Health
RTS,S: a candidate pre-blood stage malaria vaccine antigen
SAE: serious adverse event
URTI: upper respiratory tract infection
USAID: United States Agency for International Development
