# Supplementary material for: Safety and Reactogenicity of an MSP-1 Malaria Vaccine Candidate: A Randomized Phase Ib Dose-Escalation Trial in Kenyan Children
Source: PLoS Clin Trials. 2006 Nov 24;1(7):e32. doi: 10.1371/journal.pctr.0010032 (PMC1851726; doi:10.1371/journal.pctr.0010032)
Supplement: Trial Protocol [file pctr.0010032.sd002.doc]

**A double blind, randomized, controlled, dose escalation phase I field trial in 12 to 47 month old children in western Kenya**

**to evaluate the safety and immunogenicity of WRAIR’s MSP-1 (FMP-1) malaria vaccine adjuvanted in GlaxoSmithKline Biologicals’ AS02A**

**versus rabies vaccine**

**IND No.** 9202

**WRAIR Protocol No.** 1030

**GSK CPMS No.** 257049/031 (Mal-031)

## KEMRI SSC Protocol No. 761

**Sponsor:** Department of the Army, Office of the Surgeon General

**Principal Investigator**: Mark R. Withers, LTC, MC, FS

*Kenya Medical Research Institute*

*US Army Medical Research Unit-Kenya*

*Unit 64109*

*APO AE 09831-4109*

*Tel 254-57-22942*

*Fax 254-57-22903*

*Cell 254-733-619322*

*STUDY REGULATORY MILESTONES:*

Submitted to WRAIR ORM: 4 FEB 03

Submitted to WRAIR SRC: 5 FEB 03

Submitted to KEMRI CCR: 5 FEB 03

Approved by KEMRI CCR: 10 FEB 03

Submitted to KEMRI SSC: 11 FEB 03

Approved by WRAIR SRC: 3 MAR 03

Approved by KEMRI SSC: 4 MAR 03

Submitted to KEMRI ERC: 4 MAR 03

Submitted to MRMC HSRRB: 4 MAR 03

Submitted to PATH HSPC: 4 MAR 03

Approved by PATH HSPC: 21 MAR 03

Approved by KEMRI ERC: 25 MAR 03

Original Study Day 0: 28 APR 03

Approved by MRMC HSRRB (Conditional): 12 JUN 03

Approved by MRMC HSRRB (Final): 24 JUN 03

Submitted to US FDA: 27 JUN 03

“Approved” by US FDA: 7 AUG 03

Actual Study Day 0: 11 AUG 03

Table *of* Contents

List of Abbreviations [6](#__RefHeading___Toc135366546)

Glossary of Terms [9](#__RefHeading___Toc135366547)

1. TITLE OF THE PROJECT: [10](#__RefHeading___Toc135366548)

2. INVESTIGATORS & INSTITUTIONAL AFFILIATIONS: [10](#__RefHeading___Toc135366549)

3. SUMMARY: [13](#__RefHeading___Toc135366551)

4. INTRODUCTION & BACKGROUND: [14](#__RefHeading___Toc135366552)

4.1 Life Cycle of the Malaria Parasite [14](#__RefHeading___Toc135366553)

4.2 Status of Merozoite Stage Malaria Vaccines [14](#__RefHeading___Toc135366554)

4.3 Clinical Experience with the AS02A Adjuvant [16](#__RefHeading___Toc135366555)

4.4 The FMP-1 Vaccine [17](#__RefHeading___Toc135366556)

4.5 Experience with the FMP-1/AS02A Malaria Vaccine [18](#__RefHeading___Toc135366557)

**4.5.1 Pre-clinical Trials** [18](#__RefHeading___Toc135366558)

**4.5.1 Clinical Trials** [18](#__RefHeading___Toc135366559)

4.6 Justification of 0, 1, & 2 Month Dosing [20](#__RefHeading___Toc135366560)

4.7 Comparison Vaccine [20](#__RefHeading___Toc135366561)

**4.7.1** **The Vaccine** [20](#__RefHeading___Toc135366562)

**4.7.2** **Vaccine Immunogenicity** [21](#__RefHeading___Toc135366563)

**4.7.3** **Vaccine Safety** [21](#__RefHeading___Toc135366564)

4.8 Malaria in Western Kenya [22](#__RefHeading___Toc135366565)

5 JUSTIFICATION [22](#__RefHeading___Toc135366566)

6 OBJECTIVES [23](#__RefHeading___Toc135366567)

6.1 Primary objective [23](#__RefHeading___Toc135366568)

6.2 Secondary objective [23](#__RefHeading___Toc135366569)

7 STUDY DESIGN & METHODOLOGY [24](#__RefHeading___Toc135366570)

7.1 Overview [24](#__RefHeading___Toc135366571)

7.2 Number of Subjects, Number of Study Arms & Number of Study Cohorts [25](#__RefHeading___Toc135366572)

7.3 Study Center [26](#__RefHeading___Toc135366573)

7.4 Inclusion Criteria [26](#__RefHeading___Toc135366574)

7.5 Exclusion Criteria [27](#__RefHeading___Toc135366575)

7.6 Treatments Potentially Interfering with Vaccine-Induced Immunity [28](#__RefHeading___Toc135366576)

7.7 Contraindications to Vaccination [28](#__RefHeading___Toc135366577)

7.8 Indications for Deferral of Vaccination [28](#__RefHeading___Toc135366578)

7.9 Safety Plan [29](#__RefHeading___Toc135366579)

**7.9.1** **Definitions** [29](#__RefHeading___Toc135366580)

**7.9.2** **Criteria for Discontinuation of Vaccination** [29](#__RefHeading___Toc135366581)

**7.9.3** **Safety Review Prior to Dose Escalation** [30](#__RefHeading___Toc135366582)

**7.9.4** **Safety Review Prior to Administration of Consecutive Doses** [31](#__RefHeading___Toc135366583)

**7.9.5** **Procedures for Resumption of Dosing Following Discontinuation of Vaccination, Dose**

**Escalation or Administration of Consecutive Doses** [32](#__RefHeading___Toc135366584)

**7.9.6** **Dose Discontinuation in Individual Subjects** [33](#__RefHeading___Toc135366585)

**7.9.7** **Safety Precautions for Study Personnel** [35](#__RefHeading___Toc135366586)

8.0 CONDUCT OF THE STUDY [36](#__RefHeading___Toc135366587)

8.1 General Study Aspects [36](#__RefHeading___Toc135366588)

8.1.1 Screening and enrollment process [36](#__RefHeading___Toc135366589)

8.1.2 Vaccination Process [37](#__RefHeading___Toc135366590)

8.1.3 Post-immunization Evaluation Procedures [38](#__RefHeading___Toc135366591)

8.1.4 Field Workers: Activities & Competencies [39](#__RefHeading___Toc135366592)

8.1.5 Long-term Follow-up Period [39](#__RefHeading___Toc135366593)

8.2 Detailed Description of Study Stages & Visits [40](#__RefHeading___Toc135366594)

8.3 Definition and Management of Symptomatic Malaria [47](#__RefHeading___Toc135366595)

8.3.1 Definition of Symptomatic Malaria [47](#__RefHeading___Toc135366596)

8.3.2 Management of Symptomatic Malaria during the Study [48](#__RefHeading___Toc135366597)

9 SAMPLE HANDLING & ANALYSIS [49](#__RefHeading___Toc135366598)

9.1 Overview of Collection Time Points [49](#__RefHeading___Toc135366599)

9.2 Handling of Biological Samples Collected by the Investigator [50](#__RefHeading___Toc135366600)

9.2.1 Instructions for Handling of Serum Samples [50](#__RefHeading___Toc135366601)

9.2.2 Labeling [50](#__RefHeading___Toc135366602)

9.3 Laboratory Assays [51](#__RefHeading___Toc135366603)

10 STUDY VACCINES & VACCINE ADMINISTRATION [52](#__RefHeading___Toc135366604)

10.1 Study Vaccines [52](#__RefHeading___Toc135366605)

10.1.1 FMP-1 Vaccine [52](#__RefHeading___Toc135366606)

10.1.2 Imovax® Rabies Vaccine [53](#__RefHeading___Toc135366607)

10.2 Vaccine Dosage & Administration [53](#__RefHeading___Toc135366608)

10.2.1 Reconstitution of FMP-1 Vaccine [53](#__RefHeading___Toc135366609)

10.2.2 Reconstitution of Imovax® Rabies vaccine [54](#__RefHeading___Toc135366610)

10.2.3 Administration of Vaccines [54](#__RefHeading___Toc135366611)

10.2 Vaccine Storage [55](#__RefHeading___Toc135366612)

10.3 Randomization of Treatment Allocation & Age [55](#__RefHeading___Toc135366613)

10.4 Method of Blinding & Breaking the Study Blind [56](#__RefHeading___Toc135366614)

**10.5.1** **Blinding** [56](#__RefHeading___Toc135366615)

**10.5.2** **Breaking the Study Blind** [57](#__RefHeading___Toc135366616)

10.5 Replacement of Unusable Vaccine Doses [57](#__RefHeading___Toc135366617)

10.6 Vaccine Accountability [57](#__RefHeading___Toc135366618)

10.7 Concurrent Medication/Treatment [57](#__RefHeading___Toc135366619)

**10.8.1** **Drugs for Treatment of Anaphylaxis** [58](#__RefHeading___Toc135366620)

**10.8.2** **Drugs to Treat Malaria** [58](#__RefHeading___Toc135366621)

11 ADVERSE EVENTS [58](#__RefHeading___Toc135366622)

11.1 Eliciting & Documenting Adverse Events [58](#__RefHeading___Toc135366623)

**11.1.1** **Definition of an Adverse Event** [58](#__RefHeading___Toc135366624)

**11.1.2** **Surveillance Period for Occurrence of Adverse Events** [59](#__RefHeading___Toc135366625)

**11.1.3** **Recording of Adverse Events** [59](#__RefHeading___Toc135366626)

**11.1.4** **Reporting of Adverse Events** [60](#__RefHeading___Toc135366627)

**11.1.5** **Solicited Adverse Events** [60](#__RefHeading___Toc135366628)

**11.1.6** **Unsolicited Adverse Events** [61](#__RefHeading___Toc135366629)

11.2 Assessment of Intensity [61](#__RefHeading___Toc135366630)

11.3 Assessment of Causality [63](#__RefHeading___Toc135366631)

11.4 Adverse Event Follow-up & Assessment of Outcome [64](#__RefHeading___Toc135366632)

11.5 Serious Adverse Events [64](#__RefHeading___Toc135366633)

**11.5.1** **Definition of a Serious Adverse Event** [64](#__RefHeading___Toc135366634)

**11.5.2** **Reporting Serious Adverse Events** [65](#__RefHeading___Toc135366635)

11.6 Treatment of Adverse Events [69](#__RefHeading___Toc135366636)

12 SUBJECT COMPLETION & DROP-OUT [69](#__RefHeading___Toc135366637)

12.1 Definition of a Drop-out [69](#__RefHeading___Toc135366638)

12.2 Procedures for Handling Drop-outs [69](#__RefHeading___Toc135366639)

12.3 Reasons for Drop-outs [69](#__RefHeading___Toc135366640)

13 DATA MANAGEMENT & ANALYSIS [69](#__RefHeading___Toc135366641)

13.1 Primary Endpoints [70](#__RefHeading___Toc135366642)

13.2 Secondary Endpoints [70](#__RefHeading___Toc135366643)

13.3 Study Cohorts & Data Sets to be Evaluated [70](#__RefHeading___Toc135366644)

**13.3.1** **Total Cohort** [70](#__RefHeading___Toc135366645)

**13.3.2** **Safety Cohort** [70](#__RefHeading___Toc135366646)

**13.3.3** **Immunogenicity Cohort** [70](#__RefHeading___Toc135366647)

13.4 Sample Size [71](#__RefHeading___Toc135366648)

13.5.1 Analysis of Demographics [71](#__RefHeading___Toc135366649)

13.5.2 Analysis of Immunogenicity [71](#__RefHeading___Toc135366650)

13.5.3 Analysis of Safety [71](#__RefHeading___Toc135366651)

13.5.4 Clinical Laboratory Parameters [72](#__RefHeading___Toc135366652)

13.6 Preliminary Analysis [72](#__RefHeading___Toc135366653)

13.7 Administrative Matters [72](#__RefHeading___Toc135366654)

14 ETHICAL CONSIDERATIONS [73](#__RefHeading___Toc135366655)

14.1 Ethics & Regulatory Considerations [73](#__RefHeading___Toc135366656)

14.1.1 Institutional Review Board/Ethics Review Committee (IRB/ERC) [73](#__RefHeading___Toc135366657)

14.2 The Local Medical Monitor (Local Safety Monitor) [74](#__RefHeading___Toc135366658)

14.3 The Data & Safety Monitoring Board (DSMB) [75](#__RefHeading___Toc135366659)

14.3.1 Composition of the Board [75](#__RefHeading___Toc135366660)

14.3.2 Role of the Board [75](#__RefHeading___Toc135366661)

14.4 Risks & Potential Benefits to Subjects [76](#__RefHeading___Toc135366662)

14.4.1 Vaccination [76](#__RefHeading___Toc135366663)

14.4.2 Medical Treatment of Subjects [77](#__RefHeading___Toc135366664)

14.4.3 Rabies Vaccination [77](#__RefHeading___Toc135366665)

14.5 Precautions to Minimize Risk [77](#__RefHeading___Toc135366666)

14.5.1 Vaccination [77](#__RefHeading___Toc135366667)

14.5.2 Malaria Treatment During the Study [77](#__RefHeading___Toc135366668)

14.6 Procedures for Maintaining Confidentiality [78](#__RefHeading___Toc135366669)

15 REFERENCES [79](#__RefHeading___Toc135366670)

16 STUDY BUDGET [83](#__RefHeading___Toc135366671)

16.1 KEMRI Budget [83](#__RefHeading___Toc135366672)

16.2 Budget Justification [84](#__RefHeading___Toc135366673)

17 APPENDICES [85](#__RefHeading___Toc135366674)

17.1 Appendix A: Study Personnel [85](#__RefHeading___Toc135366675)

**17.1.1 Roles of Study Personnel** [85](#__RefHeading___Toc135366676)

**17.1.2 Curricula Vitae of Study Personnel [All but the PI’s have been redacted]** [86](#__RefHeading___Toc135366677)

17.2 Appendix B: Overview of Study Operational Scheme [87](#__RefHeading___Toc135366678)

**17.2.1 Operational Study Milestones** [87](#__RefHeading___Toc135366679)

**17.2.2** **Study Recruitment Plan** [87](#__RefHeading___Toc135366680)

**17.2.3** **Study Operational Field Plan** [90](#__RefHeading___Toc135366681)

**17.2.4** **Generic Schedule of Events** [91](#__RefHeading___Toc135366682)

**17.2.5** **Specific Daily Schedule of Events** [95](#__RefHeading___Toc135366683)

17.3 Appendix C: Administrative Matters [101](#__RefHeading___Toc135366684)

**17.3.1 General Administrative Matters** [101](#__RefHeading___Toc135366685)

**17.3.2 USAMRMC Specific Administrative Procedures** [105](#__RefHeading___Toc135366686)

17.4 Appendix D: Parental Informed Consent [107](#__RefHeading___Toc135366687)

**17.4.1** **MSP-1 Malaria Vaccine (FMP-1) Study Recruitment Script** [107](#__RefHeading___Toc135366688)

**17.4.2** **Parental Informed Consent Explanation** [109](#__RefHeading___Toc135366689)

**17.4.2 Parental Schedule of Events** [113](#__RefHeading___Toc135366690)

**17.4.3 Informed Consent Agreement** [116](#__RefHeading___Toc135366691)

**17.4.4**  **Consent Form for Future Research Use & Long-Term Blood Sample Storage** [118](#__RefHeading___Toc135366692)

**17.4.4**  **Identification Photograph Consent Form** [119](#__RefHeading___Toc135366693)

## List of Abbreviations

®: Registered

3D7: Clone of *Plasmodium falciparum*

ACTG: AIDS Clinical Trials Group

AE: Adverse Event

ALC: Absolute lymphocyte count

ALT: Alanine aminotransferase

Anti R32LR: Anti-CSP repeat antibodies

AS02: Adjuvant System 2 of GSK with thimerosal

AS02A: Adjuvant System 2 of GSK without thimerosal

bac. virus Baculovirus

CBC: Complete blood count

CHF: Congestive heart failure

CMI: Cell mediated immunity

CRA: Clinical Research Associate

CRF: Case Report Form

CS gene: Circumsporozoite gene of *P. falciparum*

CSP: Circumsporozoite protein

CTL: Cytotoxic T lymphocyte

DIK: Anti-gamma interferon monoclonal antibody

DSMB: Data and Safety Monitoring Board

DMSO: Dimethyl sulphoxide

DNA: Deoxyribonucleic acid

EGF; Epidermal Growth Factor

ELISA: Enzyme linked immunosorbent assay

Elispot: Method for detection of antibody/cytokine-secreting cells

ERC: Ethical Review Committee

FDA: US Food and Drug Administration

FMP-1: Falciparum Merozoite Protein 1

GSK: GlaxoSmithKline Biologicals

GMT: Geometric Mean Titer

HLA: Human leukocyte antigen

hpf: High power field

HSPC: Human Subjects Protection Committee (PATH IRB)

HSRRB: Human Subjects Research Review Board (MRMC)

HURC: Human Use Research Committee (WRAIR)

IATA: International Air Transport Association

ICH: International Conference on Harmonization

ID: Identification

IEC: Institutional Ethical Committee

IFA: Indirect fluorescent antibody

IFN-gamma : Interferon gamma

IL‑2: Interleukin 2

IM: Intramuscular; intramuscularly

IRB: Institutional Review Board

IU: International units

IU/l: International units per liter

IV: Intravenous; intravenously

KEMRI: Kenya Medical Research Institute

KSDH: Kombewa Sub-District Hospital

LDH: Lactate dehydrogenase

LMM: Local Medical Monitor

mmol/l: Millimole per liter

MPL: Monophosphoryl Lipid A

MSP-1: Merozoite surface protein 1

MVI: Malaria Vaccine Initiative

NANP: Repeat epitopes of the circumsporozoite protein

NA: Not applicable

ND: Not done

NNPGH: New Nyanza Provincial General Hospital, Kisumu, Kenya

NR: Not related

OD: Optical density

PATH: Program for Appropriate Technology in Health

*P. falciparum*: *Plasmodium falciparum*

PB: Probable

PBMC: Peripheral blood mononuclear cells

PCR: Polymerase chain reaction

PCV: Packed [red blood] cell volume

PO: Administered orally

POC: Point of contact

PPD: Pharmaceutical Product Development

QS21: *Quillaja saponaria* 21 (saponin derivative)

RAP: Reporting and Analysis Plan

RIA Radioimmunoassay

RR: Relative risk

RTS,S: A candidate malaria vaccine antigen [See next 2 entries]

RTS: Fusion protein consisting of a circumsporozoite protein-based antigen and HBsAg

S: 226 amino acid polypeptide corresponding to the surface antigen of hepatitis B virus (adw serotype)

SAE: Serious adverse event

SB Biologicals/SB Bio: SmithKline Beecham Biologicals

SB62: An oil in water emulsion

SBAS2: SmithKline Beecham Adjuvant System 2

SCI: Statistics Collaborative, Inc

Sf9 Insect cells derived from *Spodoptera frugiperda* (army

worm)

SI: *Systemè* *International* [*d’Unité*]; International System [of

Units]

SID: Subject identification (number or card)

SOP: Standard Operating Procedure

SPP: Study Specific Protocol

SQ: Subcutaneous; subcutaneously

SU: Suspected

TRAP: Thrombospondin-related anonymous protein

UL: Unlikely to be related

USAID: US Agency for International Development

USAMMDA: US Army Medical Materiel Development Activity

USAMRMC: US Army Medical Research and Materiel Command

Vacc: Vaccination

WB: Western Blot assay

WRAIR: Walter Reed Army Institute of Research, Silver Spring,

MD, USA

WRAMC: Walter Reed Army Medical Center, Washington, DC, USA

WRP: Walter Reed Project (WRAIR in Kenya)

WRP KC: WRP Kombewa Clinic

## Glossary of Terms

| **Sponsor:** | The study sponsor is the Office of the Surgeon General (OTSG), Department of the Army; OTSG authority devolves to the US Army Medical Research & Materiel Command (USAMRMC); MRMC in turn monitors regulatory compliance via the US Army Medical Materiel Development Activity (USAMMDA; POC: Dr Charles English) & its human subject research is authorized by its Human Subjects Research Review Board (HSRRB; POC: Ms. Caryn Duchesneau, Vice Acting Chair). |
| --- | --- |
| **Subject:** | Term used throughout the protocol to denote a child enrolled in the study to receive either the test vaccine or comparator vaccine |
| **Parent:** | Term used throughout the protocol to denote the adult granting informed parental consent for a child to enroll in the study |
| **Local Medical Monitor:** | A medically qualified, host country individual who ensures responsible actions with regard to the ethics and clinical safety of a study and especially in the proper assessment of adverse events |
| **Study Monitor:** | An individual who is responsible for assuring proper conduct of a clinical study at one or more investigational sites |
| **Eligible:** | In complete accord with all inclusion and exclusion criteria and, thus, qualified for enrollment into the study |
| **Evaluable:** | Eligible, having complied with all procedures defined in the protocol, and, consequently, to be included in study data analysis (Sections 13 and 13.3 have specific criteria) |
| **Enrolled:** | Term used throughout the protocol to denote a subject who is both eligible and a recipient of at least one vaccine dose. |

# TITLE OF THE PROJECT:

A double blind, randomized, controlled, dose escalation phase I field trial in 12 to 47 month old children in western Kenya to evaluate the safety and immunogenicity of WRAIR’s MSP-1 (FMP-1) malaria vaccine adjuvanted in GlaxoSmithKline Biologicals’ AS02A versus rabies vaccine

**Running Title:** MSP-1 Phase I in Children

**IND No.** 9202

**WRAIR Protocol No.** 1030

**GSK CPMS No.** 257049/031 (Mal-031)

**Sponsor:** Department of the Army, Office of the Surgeon General

# INVESTIGATORS & INSTITUTIONAL AFFILIATIONS:

**Principal Investigator**: Mark R. Withers, LTC, MC, FS

*Kenya Medical Research Institute*

*US Army Medical Research Unit-Kenya*

*Unit 64109*

*APO AE 09831-4109*

*Tel 254-57-22942*

*Fax 254-57-22903*

*Cell 254-733-619322*

**Associate Investigators:**

***KEMRI*** Jose A. Stoute, LTC(P), MC

Bernhards Ogutu, MBChB, MMed (Peds), PhD

John N. Waitumbi, D.V.M., Ph.D.

Allan Otieno, MBChB

Joram Siangla, M.Sc.

Joseph Koros, HND

Willis Okoth, B.Sc.

Stacey Okallo, RCO

Valentine Pengo, RCO

Odika J. Apollo, RCO

*P. O. Box 54, Kisumu, Kenya*

*Tel 254-57-22942*

*Fax 254-57-22903*

***WRAIR*** Robert Bowden, LTC, MSC

D. Gray Heppner, COL, MC

Kent E. Kester, LTC, MC

Christian Ockenhouse, LTC, MC

James Cummings, MAJ, MC

Jeff Lyon, Ph.D.

Alan Magill, COL, MC

Evelina Angov, Ph.D.

Carolyn Holland, MPH

*Department of Immunology, WRAIR
Tel.: 301-319-9414*

*Fax: 301-319-7358*

**GSK Biologicals** Amanda Leach, MSc, MRCPCH

Clinical Development Manager

Tel: +32-2-656-7788

Fax: +32-2-656-6160

Alfred Tiono, MD

WHO/GSK Biologicals Trainee

Tel: +32-2-656-6535

Fax: +32-2-656-6160

**Malaria Vaccine Initiative** Ms. Jessica Milman

Program Officer

Malaria Vaccine Initiative at PATH

*Tel 301 770-5377*

*Fax 301 770-5322*

**US Agency for International** Carter Diggs, M.D., Ph.D.

**Development** Senior Technical Advisor

USAID Malaria Vaccine Development Program

*Tel 202-712-5728*

Fax 202-216-3702

E-mail: cdiggs@usaid.gov

Lorraine A. Soisson, PhD
 Technical Advisor
 USAID Malaria Vaccine Development Program
 6 Van Fleet Court
 Hillsborough, NJ 08844
 *Tel: 908-369-7704
 Fax: 908-369-7724* E-mail: soisson@patmedia.net

**Research Coordinator** Melanie Onyango, B.A.

WRP/KEMRI

**Clinical Coordinator** Denise McKinney, RN, CCRC, CCRA

Visiting Scientist

WRP/KEMRI

**Local Medical Monitor:** Ambrose Misore, MBChB, MMed Peds

Nyanza Provincial Medical Headquarters
P.O. Box 721
Kisumu, Kenya *Tel.: 254-57-40091/41550
Fax: 254-57-21870*

*Alternate LMM:*

Dr. Juliana Otieno

Chief, Dept of Pediatrics

New Nyanza Provincial General Hospital

P.O.Box 849

Kisumu, Kenya

*Cell 254-733-715917*

*Fax: 254-57-41330*

**Statistical Consultants:** Janet Wittes, Ph.D.

Kathryn Tucker, M.S.

Statistics Collaborative

1710 Rhode Island Ave, NW; Suite 200

Washington DC 20036

Tel: 202-429-9267

Fax: 202-429-9268

**Study Monitors:** US Army Medical Materiel Development Activity

GlaxoSmithKline (GSK) Biologicals

**IND Sponsor**: Department of the Army, Office of the Surgeon General

**Funding Sources:** MVI

USAID

# SUMMARY:

| **Title** | A double blind, randomized, controlled, dose escalation phase I field trial in 12 to 47 month old children in western Kenya to evaluate the safety and immunogenicity of WRAIR’s MSP-1 (FMP-1) malaria vaccine adjuvanted in GlaxoSmithKline Biologicals’ AS02A versus rabies vaccine |
| --- | --- |
| **Study Population** | Healthy 12-47 month old, malaria-exposed children |
| **Study Site** | The Walter Reed Project Kombewa Clinic at Kombewa Town, Kombewa Division, Kisumu District, Nyanza Province, wertern Kenya (and two small field stations within Kombewa Division) |
| **Objectives** | ***Primary***  • To assess the safety and reactogenicity of the FMP-1/AS02A malaria vaccine in malaria-exposed children living in western Kenya and aged 12-47 months |
|  | ***Secondary***  • To assess the humoral immune response to the FMP-1/AS02A malaria vaccine in malaria-exposed children living in western Kenya and aged 12-47 months |
| **Study design** | - A phase I, dose escalation (1/5, ½, full dose) vaccine trial in children - Double blind, randomized, controlled (Imovax® Rabies vaccine). - One study center, with 2 outlying field stations |
| **Number of Subjects** | One hundred thirty-five (135) subjects of both genders will be enrolled. Screening/enrollment of subjects will be incremental until the desired number of subjects has been reached. |
| **Number of**  **Cohorts** | Each of 3 study cohorts will consist of 30 children receiving a dose (10, 25, or 50 μg; Cohorts A, B, and C, respectively) of the study test article and 15 child-ren receiving the comparator vaccine (45 subjects per cohort). All test article and comparator groups will be further divided into 3 age groups (12-23, 24-35, and 36-47 months) of 10 (test article) or 5 (comparator) subjects each. |
| **Immunization**  **Schedule** | 0, 1, and 2 months (Provisionally: Study Days 0, 29, and 57 for Cohort A; 14, 42, and 70 for Cohort B; 28, 56, and 84 for Cohort C) |
| **Study Duration** | 12 months per subject |
| **Route** | IM injection into (alternately) right & left anterolateral thigh muscles |
| **Primary endpoint** | The primary endpoints are measures of the safety and reactogenicity of the vaccine as determined by:  • Occurrence of solicited symptoms during a 7 day follow-up period after each vaccination (5 visits: day of vaccination and days 1, 2, 3, and 7)  • Occurrence of unsolicited symptoms during a 30 day follow-up period after each vaccination (day of vaccination and the 29 subsequent days)  • Occurrence of serious adverse events during an 8 month follow-up period  following the first dose of study vaccine |
| **Secondary endpoints** | •    Antibody responses to MSP-1 by ELISA following immunization with the study vaccine through 364 days following the first dose of study vaccine |

# INTRODUCTION & BACKGROUND:

## Life Cycle of the Malaria Parasite

The malaria parasite has a complex life cycle. The bite of an infectious mosquito transmits the sporozoite form of the parasite to humans. These sporozoites travel through the bloodstream and ultimately invade liver cells where they multiply asexually as exoerythrocytic stage parasites. The exoerythrocytic forms of *Plasmodium falciparum* mature in five to seven days releasing thousands of tissue merozoites that invade erythrocytes and initiate the erythrocytic phase of the infection. The free merozoites invade erythrocytes, undergo asexual maturation, and ultimately rupture the erythrocytes releasing new merozoites. It is this phase, characterized by cyclic destruction of erythrocytes, that results in the clinical disease known as malaria. A small number of the invading merozoites do not multiply, but instead differentiate into sexual forms known as gametocytes. When ingested by a female mosquito, male and female gametocytes can unite to form a zygote, which then matures to release sporozoites. These subsequently migrate to mosquito salivary glands where they are available to infect the next person to be bitten, thus completing the parasite’s life cycle.

## Status of Merozoite Stage Malaria Vaccines

Malaria is a major world-wide problem with transmission occurring throughout Africa, Asia, Oceania, and Latin America. Over two billion people are estimated to live in malarious areas of the world. In Africa, for example, an estimated 300 to 500 million cases of malaria occur each year leading to about 1.5-2.7 million deaths (1). Sturchler estimated that there were 489 million clinical cases of malaria during 1986. Of these, 234 million (48%) were thought to be due to *P. falciparum* with more than 2.3 million fatalities (2).

The objective of developing an effective malaria vaccine has been a major focus of malaria research for many years. This task has proven to be more difficult than initially thought. To date, there is no effective malaria vaccine on the horizon although significant progress has been made. Two critical steps of the malaria life cycle have been closely examined as potential targets for vaccine-induced immunity: (1) the invasion of hepatocytes by sporozoites and (2) the invasion of erythrocytes by merozoites.

The merozoite stage of the parasite, like the sporozoite, is a logical target for a malaria vaccine since blockade of erythrocyte invasion would completely prevent clinical disease. Therefore, the identification of the molecular mechanisms of merozoite invasion of red blood cells has been an active area of investigation in the field of malaria vaccine research. Several antigens have been identified that have the potential to inhibit merozoite invasion of red cells. The most studied of these antigens, and also the most promising blood stage vaccine candidate, is the merozoite surface protein 1 (MSP-1), a 195 kDa antigen found on the surface of merozoites. It undergoes processing by proteolytic cleavage to a 42 kDA fragment and further to a 19 kDa fragment that has been implicated in the invasion of erythrocytes by the merozoite (3). Several lines of evidence lead to the conclusion that MSP-1 is a promising vaccine candidate. Antibodies directed against portions of MSP-1, in particular against the 19 kDa c-terminal fragment, inhibit erythrocyte invasion (3, 4). At least one field study has demonstrated an association between the existence of antibodies against MSP-1 and resistance to clinical malaria (5). Immunization with recombinant fragments of this molecule also protects monkeys against *P. falciparum* (6), when used with Complete Freund’s Adjuvant, and mice against *P. yoelii* (7, 8). Passive transfer of immune sera in mice also confers protection (9). Although the weight of the evidence indicates that antibodies against the c-terminal fragment of MSP-1 are protective, in one case immunization with c-terminal constructs did not result in protection (10). Lack of protection in some cases could be due to the use of antigenic constructs that do not have a proper conformational structure since this region of MSP-1 is known to be conformationally dependent. MSP-1 is also the target of CD4^+^ T cells and several T-cell epitopes have been identified (11, 12).

A recombinant version of the 42 kDA c-terminal portion of MSP-1 has been produced at the Walter Reed Army Institute of Research. This falciparum merozoite protein 1 (FMP-1) is produced as a histidine-tagged (His6) fusion protein in *Escherichia coli*. The antigen is derived from the 3D7 clone of *P. falciparum* and contains both T-cell and B-cell epitopes. Monoclonal antibodies raised against native parasite MSP-1 recognize correctly folded conformational disulfide-bonded epitopes within the recombinant 42 kD antigen. Additionally, the structural fidelity of this preparation has been confirmed by demonstrating that the recombinant 42 kD antigen binds specifically to human erythrocytes in a manner analogous to native parasite-derived MSP-1 binding to red blood cells. An efficient fermentation and purification process has been developed for the production of this antigen on a scale compatible with industrial manufacture. The vaccine is formulated in the same adjuvant system used in the RTS,S vaccine, now called AS02A (the second ‘A’ denotes absence of thimerosal). In order to increase product stability, the MSP-1_42_ antigen is manufactured as a lyophilized product and reconstituted just prior to injection.

The study proposed here is a Phase I safety and immunogenicity study of FMP-1 in a malaria endemic area of western Kenya. If shown to be safe and immunogenic, further studies will be pursued with this same formulation to assess its efficacy singly or in combination with RTS,S.

## Clinical Experience with the AS02A Adjuvant

The adjuvant system now called AS02A was previously known as SBAS2 and later as AS02. (The new designation indicates the absence of thimerosal in a new formulation.) It consists of an oil-in-water emulsion combined with two immuno-stimulants: (1) monophosphoryl lipid A (MPL) and (2) a saponin derivative known as QS21. The latter is a highly purified component of a saponin agent derived from the soap bark tree, *Quillaja saponaria* (13, 14). MPL is a detoxified (deacylated) form of monophosphoryl lipid A. Lipid A is a component of *Salmonella minnesota* lipopolysaccharide (LPS). LPS, and more specifically, its lipid A component, has long been known for its strong adjuvant effects. However, until recently, its high toxicity precluded its use in a vaccine formulation. Ribi et al. (15), showed that the monophosphorylated form of lipid A retains its adjuvant function and almost completely loses its endotoxin effects. Subsequently, the 3-deacylated form of MPL (3D-MPL) was shown to have a further decrease in its toxicity as tested in small animals, but retains its immunopotentiating effect (16). Several immunogenicity studies performed in mice, guinea pigs, monkeys, and humans have shown that inclusion of MPL into a vaccine preparation potentiates both specific antibody and cellular immune responses (16-18). The term MPL in this protocol refers to the 3-deacylated form of the compound. To date, the bulk of the experience with this formulation in malaria vaccines has been in conjunction with the RTS,S antigen cited above.

In a study conducted in malaria-naive subjects at the WRAIR, the RTS,S/SBAS2 formulation of the vaccine protected 6 out of 7 subjects who were challenged with a homologous NF54 (clone 3D7) strain of *P. falciparum* approximately 3 weeks after receiving a third dose of this vaccine (19). By contrast, all of 6 subjects who were not immunized were subsequently challenged with the same laboratory strain of *P. falciparum* and all developed parasitaemia.

Administration of RTS,S/SBAS2 in the past has been associated with some local signs of inflammation such as mild to moderate tenderness, swelling, and leg motion limitation that required the use of analgesics in selected subjects. In the first clinical trial (19) of the liquid version of RTS,S/SBAS2, two subjects were noted to have severe reactions. One subject with an antecedent history of migraine headaches presented with an uncomplicated migraine headache 7 days after the first dose of vaccine. A second subject presented with headache, fever, chills, myalgias, nausea, vomiting, photophobia, and severe pain at the injection site within 24 hours of receipt of the second vaccine dose. She had previously been diagnosed with aseptic meningitis 4 months prior to entry into the study. Extensive laboratory and radiological analyses, including computerized axial tomography of the head and cerebrospinal fluid studies, were unrevealing. This subject was treated with analgesic compounds and excluded from receipt of further doses of RTS,S/SBAS2. For safety reasons, a decision was made to reduce the final dose of RTS,S/SBAS2 to 0.1 ml from the standard 0.5 ml dose.

**[Material has been redacted here]**

Data generated in phase I/II vaccine trials involving malaria-experienced subjects in the Gambia (22) and Kenya (23) showed no increased reactogenicity after the administration of a full third dose of 0.5 ml of RTS,S/SBAS2 compared to that after the second dose, and no serious adverse events related to vaccination have been reported in these study populations.

Following demonstration of the safety, immunogenicity and efficacy of the RTS,S/AS02A vaccine in adults, the candidate vaccine progressed to clinical evaluation in children in The Gambia, with the first study in children aged 6-11 years (Malaria-015) and a subsequent study in toddlers and children aged 1-5 years (Malaria-020). Both studies were double-blinded, randomized and controlled (rabies vaccine). A dose-escalation approach was adopted using 1/5 (0.1 ml), 1/2 (0.25 ml) and full (0.5 ml) dose volumes of RTS,S/AS02A in order to ensure maximum safety.

In total, safety data have been generated following the administration of 60, 59 and 60 doses of 1/5 (0.1 ml), 1/2 (0.25 ml) and full (0.5 ml) RTS,S/AS02A dose volumes respectively to 20 children aged 6-11 years in each dose group, and 89, 89 and 89 doses of 1/5 (0.1 ml), 1/2 (0.25 ml) and full (0.5 ml) RTS,S/AS02A dose volumes respectively to 30 toddlers and children aged 1-5 years in each dose group.

**[Material has been redacted here]**

In all, these studies, with a cumulative experience of over 1,000 non-immune and semi-immune subjects, suggest that the AS02A adjuvant system has an excellent safety profile.

## The FMP-1 Vaccine

The falciparum merozoite protein 1 (FMP-1) study vaccine is a lyophilized recombinant antigen produced in, and purified from, *Escherichia coli* bacteria at the Walter Reed Army Institute of Research, Silver Spring, MD, USA. This antigen consists of the 42-kDa carboxy-terminal end (392 amino acids) of the merozoite surface protein-1 (MSP-1_42_) from the malaria parasite, *Plasmodium falciparum,* 3D7 clone. The protein is expressed as a fusion protein in which 6 histadines are added to the N terminal to facilitate purificat-ion. The FMP1 vaccine product used in the previous RTS,S/FMP1 combination trial (Malaria-019; under FDA IND #9772), in the previous phase I trial in adults at Kombewa (Malaria-024; under FDA IND #9202), and to be used in the present study, is designated BPR-425-00, Lot 0770. The lyophilized antigen will be dissolved in 0.5 ml of AS02A adjuvant prior to injection.

The total amount of antigen contained in a single adult dose has been 50 μg. This dose was chosen on the basis of results from phase I studies in malaria-naïve adult subjects as described in Section 4.5.1. The long-term strategy is to use the lowest practical dose. Data from some other approved vaccines (e.g., hepatitis B vaccine) supports the use of smaller-than-adult doses in children. (The specific smaller doses selected – 10 and 25 μg -- are principally related to ease of ability to accurately determine the antigen concentration in dilute solutions.)

## Experience with the FMP-1/AS02A Malaria Vaccine

### **4.5.1 Pre-clinical Trials**

The FMP-1/AS02A formulation was safe, well tolerated, and highly immuno-genic in a preclinical trial conducted in rhesus macaques (*Macaca mulatta*) performed at the Armed Forces Research Institute of the Medical Sciences (AFRIMS) in Bangkok, Thailand. Eight monkeys were immunized intramuscularly on a 0, 1, 3 month schedule using a standardized safety and immunogenicity model. All eight immunized monkeys seroconverted to the immunogen. Group mean antibody titers against a 19-kDa subunit of the immunogen rose to 10,000 ELISA units after 2 doses and to 17,000 units after 3 doses. Rhesus antibody against *Plasmodium falciparum*-parasitized red blood cells was also highly positive in an indirect fluorescence antibody assay.

Clinical grade lots of MSP-1_42_, adjuvanted with AS02A and mixed, have been administered to mice and guinea pigs. No significant local or systemic toxicities were observed in any of the animals. Immune responses to the formulation in mice indicated excellent antibody responses to the FMP-1 antigen.

### **4.5.1 Clinical Trials**

An open label, phase I, dose-escalation study to evaluate the safety, reactogenic-ity, and immunogenicity of FMP-1 with AS02A adjuvant was undertaken in October 2000 at the WRAIR. Fifteen human subjects were randomized to receive either 10μg (N=5), 25μg (N=5), or 50μg (N=5) doses of vaccine on a 0, 1, 3 month schedule by IM injection. The ratio of adjuvant to antigen was constant (i.e., 0.10 ml, 0.25 ml, or 0.50 ml of AS02A, respectively). After 3 doses, there were no grade 3 reactions (defined as a reaction that prevents normal day-to-day activities) and no serious adverse events. The laboratory tests have been normal except for an occasional elevated CPK level detected both at the time of immunization and 48 hours after immunization probably related to physical activity of the subjects. Seroconversion occurred in all 15 individuals after a single immunization. Boosting of antibody levels occurred after the second and third doses. The antibody titers against MSP-1_42_ are summarized below in OD units (the dilution that gives and OD_415_= 1).

| **Table 1: Immunogenicity (OD Units) of 3 different doses of the FMP-1/AS02A vaccine**  (N=5 in each arm) | | | | | | | | | | | | |
| --- | --- | --- | --- | --- | --- | --- | --- | --- | --- | --- | --- | --- |
| **Cohort** |  | **Day 0** |  | **Day 14** |  | **Day 28** |  | **Day 42** |  | **Day 84** |  | **Day 98** |
| 1/5 Dose (10 μg) |  |  |  |  |  |  |  |  |  |  |  |  |
| Average |  | 12 |  | 312 |  | 462 |  | 18066 |  | 7371 |  | 32648 |
| Std Dev |  | 8 |  | 191 |  | 236 |  | 8406 |  | 6304 |  | 24046 |
| Geo Mean |  | 10 |  | 272 |  | 412 |  | 16440 |  | 5749 |  | 26626 |
|  |  |  |  |  |  |  |  |  |  |  |  |  |
| 1/2 Dose (25 μg) |  |  |  |  |  |  |  |  |  |  |  |  |
| Average |  | 28 |  | 1285 |  | 2530 |  | 44172 |  | ND |  | 57771 |
| Std Dev |  | 6 |  | 1882 |  | 2631 |  | 21176 |  | ND |  | 24192 |
| Geo Mean |  | 28 |  | 636 |  | 1762 |  | 40744 |  | ND |  | 53569 |
|  |  |  |  |  |  |  |  |  |  |  |  |  |
| Full Dose (50 μg) |  |  |  |  |  |  |  |  |  |  |  |  |
| Average |  | 32 |  | 688 |  | 990 |  | 32461 |  | 15914 |  | 50053 |
| Std Dev |  | 32 |  | 414 |  | 306 |  | 19307 |  | 5650 |  | 29991 |
| Geo Mean |  | 22 |  | 586 |  | 951 |  | 28448 |  | 14938 |  | 42799 |

Although the above results suggest little or no difference between recipients of 25 and 50 μg, IFA titers against whole merozoites were higher in the latter group than in the former. Because antibody titers against whole merozoites may be more relevant, the doses of 10, 25 and 50 μg have again been chosen as the doses for the phase I trial pro-posed in the present protocol.

As a consequence of the safety and immunogenicity results from the open-label FMP-1/AS02A phase I, dose-escalation study, a double blind, phase I/IIa was also con-ducted at the WRAIR to evaluate the potential synergy of FMP-1/AS02A and RTS,S/ AS02A. Sixty subjects were randomized into 4 groups of 15 each, and received vaccines on a 0, 1, and 3 month schedule. The first group received separate arm injections of FMP-1/AS02A and AS02A; the second group separate arm injections of RTS,S/AS02A and AS02A; the third group separate arm injections of FMP-1/AS02A and RTS,S/ AS02A; and the fourth group an extemporaneous mixture of FMP-1/RTS,S/AS02A in separate arms. A fifth group (N=12) served as infectivity controls. Study endpoints included safety, immunogenicity, reactogenicity, and efficacy (defined as either delay or prevention of parasitemia in comparison to controls as determined by light microscopy). The purpose of the primary efficacy analysis was to determine major agonist or antagon-ist effects of FMP-1 on RTS,S/AS02A-mediated protection. Secondary analyses include-ed experimental molecular analyses to determine delay in release of hepatic merozoites, and correlation of efficacy results with the functional and quantitative antigen-specific antibody. The study began in April 2001 and the final results have not been published, but the protective effects seen in the RTS,S group and both RTS,S/FMP-1 groups were all similar (Personnel communication, Dr James Cummings, WRAIR).

## Justification of 0, 1, & 2 Month Dosing

Most previous FMP-1 studies have been conducted using a 0, 1, and 3 month immunization schedule. Our previous phase I trial in 40 adults in Kombewa District, Kenya used a 0, 1, and 2 month schedule. Our overall FMP-1 testing program, although initially directed at adults, aims ultimately at evaluating the vaccine in children. With this goal in mind, the more accelerated schedule anticipates that such intervals will ultimately be more amenable to incorporation into the World Health Organization’s Expanded Program of Immunizations (EPI).

For justification of the 0, 1, and 2 month dosing of the comparator vaccine, see Section 4.7.2.

## Comparison Vaccine

### **The Vaccine**

A rabies vaccine (Imovax® Rabies vaccine) will serve as the comparator in this study. Imovax® is produced by Aventis Pasteur, SA and the recommended doses and schedules are the same for infants, children and adults. (See Section 10.2 for more details.) Rabies vaccine is a parenteral preparation of inactivated rabies virus used to promote active immunity to rabies in individuals exposed to the disease or virus. Prophylactic doses of rabies vaccine are administered to certain groups of people at high-risk for acquiring rabies infection. Advantages of providing preexposure vaccination include reduced number of doses of the vaccine after a rabies exposure, elimination of need for rabies immune globulin postexposure, possible protection in persons whose post-exposure therapy is delayed or unavailable, and possible protection for persons at risk for inapparent exposures to rabies. Preexposure vaccine is recommended for individuals who are at risk of occupational exposure to the rabies virus (e.g., physicians, veterinarians, laboratory workers, animal handlers). Preexposure vaccination is also recommended for other persons whose activities involve frequent contact with rabies virus or potentially rabid animals such as bats, raccoons, skunks, cats, dogs, or other species at risk for having rabies. Preexposure vaccination is commonly recommended in international travelers who are likely to come in contact with animals in areas where dog rabies is enzootic and immediate access to appropriate medical care, including biologics, might be limited.

Receipt of Imovax® is expected to benefit this population of children as rabies is prev-alent throughout Kenya and is a particular concern in children subjected to dog bites. The investigators have elected to use rabies vaccine over hepatitis B vaccine as the comparat-or. A hepatitis B vaccine was recently introduced into western Kenya as part of the Kenya Expanded Programme on Immunizations (KEPI) and the investigators are aware that most of the Kombewa children now 12-24 months of age have received it. Rabies is prevalent throughout Kenya due to the presence of many unvaccinated dogs (24). There-fore, the investigators believe that rabies immunization may be more beneficial to this local population.

Although only the 45 comparison subjects will receive this comparator vaccine during the active phase of the study, it will also be offered free of charge to the remaining 90 children after study completion.

### **Vaccine Immunogenicity**

When Imovax® is administered according to the recommended immunization schedule (days 0, 7, 21), nearly 100% of subjects attain a protective titer. In two studies carried out in the US in 101 subjects, antibody titers > 0.5 IU/ml were obtained by day 28 in all subjects. In studies carried out in Thailand in 22 subjects, and in Croatia in 25 subjects, antibody titers of > 0.5 IU/ml were obtained by day 14 (injections on days 0, 7, 21) in all subjects (25-29). High antibody titers have also been demonstrated with off-label immunization with Imovax® Rabies. Among participants in England, Germany, France and Belgium who received two doses one month apart, nearly 100% of the participants developed specific antibody and the geometric mean titer for the group was 10 IU (30-33). The proposed immunization schedule of 0, 1, and 2 months is therefore expected to be highly successful in conferring protective immunity against rabies among the control participants.

### **Vaccine Safety**

Both safety and efficacy have been established for Imovax® Rabies vaccine in children. Local and/or mild systemic reactions may occur after injection, but these are usually transient and do not contraindicate continuing immunization. Imovax® is a human diploid cell vaccine (HDCV). In a study using 5 doses of HDCV, local reactions such as pain, erythema, and swelling or itching at the injection site were reported in about 25% of recipients (35).Mild systemic reactions such as headache, nausea, abdominal pain, muscle aches, and dizziness were reported in about 20% of recipients (35). Two cases of neurologic illness resembling Guillan-Barre syndrome, a transient neuroparalytic illness, and a focal subacute central nervous system disorder temporally associated with HDCV have been reported (36-38).

Systemic allergic reactions characterized by generalized urticaria and in some cases by arthralgia, angioedema, fever, nausea and vomiting have been reported following administration of HDCV. These reactions are uncommon in primary administrations but have been reported in up to 7% of persons receiving a booster dose (39).

## Malaria in Western Kenya

Malaria in western Kenya is holoendemic. Intense transmission occurs primarily by bites of the *Anopheles gambiae* mosquito. *P. falciparum* parasitemia is present in over 90% of malaria cases. *P. ovale* and *P. malariae* together constitute less than 10% and are usually present in mixed infections with *P. falciparum.* Malaria transmission occurs all year but at a very high level during the two rainy seasons. The “long rainy season”of late March through May, produces intense transmission from April through August; the “short rainy season” of October through December produces another, somewhat less intense, transmission season from November through January. The intensity of both of these transmission seasons can be predicted by the amount of rain seen in the preceding month and correlates with both the total population of female anopheline mosquitoes and the entomological inoculation rate (EIR, i.e., the number of infected mosquito bites per person per day). Cumulative malaria attack rates have been in the order of 95% during the long rains and 75% during the short rains. The adult population of several villages not far from the study site has been subject of several malaria chemotherapy and epidemi-ology studies. These studies typically demonstrate about 90% incidence of parasitemia over 12 weeks during a high transmission period. Approximately 25% of these infected adults will become symptomatic at some time during that period. A large-scale longitud-inal cohort project recently undertaken in Kisumu District (Bloland PB, Boriga DA, Ruebush TK, et al, “Longitudinal cohort study of the epidemiology of malaria infections in an area of intense malaria transmission II. Descriptive epidemiology of malaria infection and disease among children.” *Am J Trop Med* *Hyg* 1999 Apr; 60(4):641-8) documented, over the course of two disease seasons, that the malaria parasite prevalence in the proposed study district and in the proposed study age groups (1-4 year olds) was 83%. All individuals in this population are parasitemic multiple times over a lifetime. There is no doubt whatever that children in this population are “malaria-exposed”.

The Walter Reed Project (WRP) and the Kenya Medical Research Institute (KEMRI) have been involved in malaria research in western Kenya for many years. These studies cover nearly every aspect of the disease: epidemiology, entomology, immunology, hospital-based treatment trials, and community-based studies of antimalarials. The WRP/KEMRI laboratories near Kisumu have served as the base for both phase I and II trials of candidate malaria vaccines and antimalarial drugs over the past decade. In spite of these research efforts, malaria infections in this area continue unabated and improved control strategies are required.

# JUSTIFICATION

Children are the population with the highest rates of morbidity & mortality due to malaria, accounting for more than 1 million deaths in sub-Saharan Africa. Malaria is a very significant cause of morbidity and mortality in both adults and children in western Kenya. This area is understood to be one in which the burden of malaria falls particularly hard on the younger age groups, thereby making these groups unusually amenable to scientific study. Worldwide, the populations at risk for malaria include not only the infants, children and adults in malaria-endemic regions, but also non-immune travelers to malarious areas including vacationers and deploying military personnel. A safe and effective vaccine that prevented infection, or even merely clinical symptoms, of *P. falcip-arum* malaria would be a milestone public health achievement and would become a mainstay in efforts to control this most serious infectious disease around the world. The increasing prevalence of drug resistance in various malaria strains makes an effective vaccine an international priority in the struggle for control of this devastating disease.

The target population for this particular vaccine is children at risk for clinical disease (including severe disease) due to infection with *P. falciparum*. MSP-1 can only be tested for proof of concept in this population, as there is no predictive animal model and no reliable challenge system to detect efficacy against clinical malaria. This vaccine has been tested in malaria naïve adults (US) to establish safety, reactogenicity and to identify a dose for further evaluation. It was subsequently evaluated in malaria-exposed adults (Kenya) & has been found to be safe, well tolerated & immunogenic in this pop-ulation. The AS02A adjuvant has been tested in combination with the RTS,S vaccine in adults & children aged 1 year & older with prior (and ongoing) exposure to *P. falciparum* malaria. This AS02A-containing vaccine has been found to be safe & well tolerated.

The next logical step in the clinical development of FMP-1/AS02A is its introduct-ion into the pediatric population in a cautious, step-wise manner as described in this protocol.

# OBJECTIVES

## Primary objective

- To assess the safety and reactogenicity of the FMP-1/AS02A malaria vaccine in 12-47 month old, malaria-exposed children living in western Kenya

## Secondary objective

- To assess the humoral immune response to the FMP-1/AS02A malaria vaccine in 12-47 month old, malaria-exposed children living in western Kenya

# STUDY DESIGN & METHODOLOGY

## Overview

- Double blind, randomized, controlled, dose escalation, phase I study of a candidate vaccine/adjuvant
- One study center, with 2 outlying (satellite) field stations
- Screening will be incremental & will be done within 35 days prior to the first inoculation for each cohort.
- 135 subjects will be divided equally among 3 cohorts (1/5, ½ or full dosage groups: 10, 25 or 50 μg, respectively) of 30 subjects each and a comparison group of 15 subjects (Total subjects per cohort = 45).
- Each cohort will be stratified by age group (12-23, 24-35, and 36-47 months) (Total subjects in each of 3 age groups [among all cohorts]= 45).
- The comparison groups (total of 45 subjects in all 3 age groups) will receive Imovax® Rabies vaccine.
- Immunization schedule will be 0, 1, and 2 months for all cohorts (Provisionally: Study days 0, 29 and 57 for Cohort A; 14, 42, 70 for Cohort B; and 28, 56, 84 for Cohort C)
- Vaccinations of Cohorts B and C (FMP-1 doses of 25 ug and 50 ug respectively, with control subjects) will be offset (“staggered”): Cohort B following Cohort A by 2 weeks and Cohort C following Cohort B by 2 weeks. These intervals may be extended for up to 5 weeks, however, if *temporary discontinuation* (see Section 7.9.1) is deemed advisable due to SAEs or other concerns.
- Subjects in each cohort will be randomized separately from the other cohorts, at the times of first vaccinations.
- Route of inoculation will be by IM injection into right or left (alternately) antero-lateral thigh muscles (unless compelling reason for an alternate injection site is evident).
- Study duration will be approximately 12 months per subject.
- Seven (7) day follow-up period for solicited adverse events (5 visits: Vaccination day plus days 1, 2, 3 and 7 post-vaccination)
- Thirty (30) day follow-up period for unsolicited adverse events (Vaccination day plus 29 subsequent days)
- Follow-up of serious adverse events (SAE’s) for 8 months after the first dose of study vaccine (6 months after dose 3)
- At the end of the follow-up period for unsolicited AEs (i.e., after the third dose; Study day 90), subjects will be followed by field workers at home at monthly intervals and will be asked to return to clinic every 3 months for long-term clinical and immunogenicity follow-ups.

## Number of Subjects, Number of Study Arms & Number of Study Cohorts

The study will randomize 135 subjects, irrespective of gender, who fulfill all enrollment criteria. These subjects will be recruited into one of 3 cohorts (dosage groups), each containing a total of 45 subjects. Each cohort will be composed of 15 subjects in each of 3 age groups (12-23 months, 24-35 months, and 36-47 months; monthly ages are rounded down, e.g., a child who is 24.5 months old is considered to be 24 months old). Moreover, each cohort will be composed of 30 FMP-1 recipients (either 10 μg, 25 μg or 50 μg) and 15 Imovax® (comparator vaccine) recipients.

Age-stratification will be maintained down to the smallest groups; i.e., an FMP-1 to Imovax® ratio of 10: 5 will be maintained in each age-group of a given cohort. Recruitment of subjects into each age-group of a cohort will be separately incremental (i.e., recruitment will not stop in any age group of a cohort until 15 children have been recruited, randomized, and vaccinated). Recruitment will stop altogether when the total number of subjects enrolled has reached 135.

Cohort A: The 30 subjects receiving 1/5^th^ dose = 10 μg of FMP-1 (10 subjects from each of the 3 age groups), plus the 15 subjects receiving the Imovax® comparator (5 subjects from each of the 3 age groups), together constitute Cohort A.

Cohort B: The 30 subjects receiving ½ dose = 25 μg of FMP-1 (10 subjects from each of the 3 age groups), plus the 15 subjects receiving the Imovax® comparator (5 subjects from each of the 3 age groups), together constitute Cohort B.

Cohort C: The 30 subjects receiving full dose = 50 μg of FMP-1 (10 subjects from each of the 3 age groups), plus the 15 subjects receiving the Imovax® comparator (5 subjects from each of the 3 age groups), together constitute Cohort C.

These age groups, study arms and cohorts are presented in tabular fashion in Tables 2 and 3.

**Table 2: Numbers of Subjects by Cohorts, Study (Dosage) Arms & Age Groups**

|  | **Receiving Test**  **(FMP-1) Vaccine** | | | | **Receiving Comparator (Rabies) Vaccine** | | |
| --- | --- | --- | --- | --- | --- | --- | --- |
| Ages (Months) | **Dose** | 12-23 | 24-35 | 36-47 | 12-23 | 24-35 | 36-47 |
| **Cohort A** | 10 μg | 10 | 10 | 10 | 5 | 5 | 5 |
| **Cohort B** | 25 μg | 10 | 10 | 10 | 5 | 5 | 5 |
| **Cohort C** | 50 μg | 10 | 10 | 10 | 5 | 5 | 5 |
| **Age Group Totals** |  | **30** | **30** | **30** | **15** | **15** | **15** |
| **Vaccine Group Totals** | **90** | | | | **45** | | |
| **Grand Total** | **135** | | | | | | |

**Table 3: Numbers of Subjects by Cohorts & Age Groups**

|  | **Total Vaccinated**  (FMP-1 & Rabies)  in each age group | | | **Totals**  in each cohort |
| --- | --- | --- | --- | --- |
| Ages (Months) | 12-23 | 24-35 | 36-47 | **45**  **45**  **45** |
| **Cohort A** | 15 | 15 | 15 |
| **Cohort B** | 15 | 15 | 15 |
| **Cohort C** | 15 | 15 | 15 |
| **All Cohorts** | **45** | **45** | **45** |
| **Grand Total 135** | | | | |

## Study Center

The Walter Reed Project (WRP) Kombewa Clinic (WRP KC) is located in the village of Kombewa, in Kombewa Division, Kisumu District, Nyanza Province. Kom-bewa Town is about 40 kilometers west of Kisumu Town.

The WRP KC is located directly across the Kombewa-Maseno road (a murram road) from the Kombewa Sub-District Hospital (KSDH) and it is less than 1 kilometer north of the Kisumu-Bondo road (which is macadamized). The KSDH is a Ministry of Health sub-district hospital. It is an outpatient and inpatient facility with a 24-26 bed capacity. The director is a Clinical Officer (physician assistant equivalent) assisted by a team of 9 nurses. The facility is staffed 24 hours/day and has a basic laboratory that can do malaria smears for diagnosis.

The WRP KC, the study center itself, is readily accessible by foot or ground vehicle to all study subjects. It consists of a clinical laboratory equipped to carry out malaria diagnosis and basic clinical laboratory assays (such as measurement of serum chemistries and complete blood counts), a phlebotomy room, an outpatient facility that includes 6 examination/consultation rooms, a pharmacy, an active records room, and an archive. Source documents and CRFs will be kept in locked cabinets in the latter two rooms. Additionally, the facility has a dedicated data entry room, offices for clinical officers and clinical coordinators, on-call rooms, an urgent treatment room, a 4-bed overnight-care ward, a wash room, a general store, and a kitchen with dining facility. The PI, associate investigators, and clinical officers who are experienced clinicians will carry out the clinical evaluations, including laboratory evaluations, in this facility. A clinical staff member will be available there 24 hours/day and 7 days/week during active studies.

## Inclusion Criteria

- A healthy male or female child, 12 to 47 months of age at the time of screening.
- Written informed consent obtained from at least one parent before study start.
- Available to participate for the duration of the study (12 months).

## Exclusion Criteria

**The following criteria should be checked at the time of study entry. If any apply at the time of study entry, the subject must not be included in the study:**

- Acute disease at the time of entry into the study
- Axillary temperature of ≥37.5°C
- Respiratory rate ≥ 50
- Serum ALT ≥ 45 IU/l (i.e., > 1.5 X ULN)
- Decreased renal function: serum creatinine levels > 92.2 mM/l (> 1.1 mg/dl).
- Significant anemia (Hgb <8 gm/dL).
- Thrombocytopenia (Platelets < 100,000 per mm^3^)
- Impaired immunity: (Absolute lymphocyte count [ALC] for 1 year olds < 4.0 x 10^3^/mm^3^; for 2 year olds < 3.0 x 10^3^/mm^3^; for 3 year olds < 2.0 10^3^/mm^3^.
- History of homozygous sickle cell disease (SS).
- Malnutrition (Z score; Malnutrition = Weight for height < - 3 z scores)
- Blood transfusion or use of blood-based product in previous 6 months.
- Prior receipt of a rabies vaccine or an investigational malaria vaccine.
- Use of any investigational drug or vaccine other than the study vaccine within 30 days preceding the first dose of study vaccine, or planned use up to 30 days after the third dose.
- Administration of chronic (defined as more than 14 days) immunosuppressants or other immune-modifying drugs within six months of vaccination. (For cortico-steroids, this will mean prednisone, or equivalent, greater than or equal to 0.5 mg/kg/day. Inhaled and topical steroids are allowed).
- Administration or anticipated administration of a vaccine not foreseen by the study protocol within 30 days of the first dose of vaccine(s) with the exception of tetanus toxoid.
- Previous vaccination with a vaccine containing MPL or QS21 (e.g., RTS,S).
- Any confirmed or suspected immunosuppressive or immunodeficient condition, including human immunodeficiency virus (HIV) infection. (No HIV testing will be undertaken as part of this study.)
- History of allergic reactions or anaphylaxis to immunizations or to any vaccine components.
- History of surgical splenectomy.
- Administration of immunoglobulins or any blood products within the 3 months preceding the first dose of study vaccine or planned administration during the study period.
- Simultaneous participation in any other clinical trial.
- Acute or chronic cardiovascular, pulmonary, hepatic or renal condition, which in the opinion of the PI may increase the risk to the subject from participating in the study.
- Any other condition or circumstance that in the opinion of the investigator may pose a threat to the subject.

## Treatments Potentially Interfering with Vaccine-Induced Immunity

**The following criteria should be checked at each visit. If any become applicable during the study, the subject will not be required to discontinue, but a separate analysis may be done which excludes these individuals. See Section 13.3 for definitions of study cohorts and data sets to be evaluated.**

- Use of any investigational drug or vaccine other than the study vaccine during the study period.
- Administration of chronic (defined as more than 14 days) immunosuppressants or other immune-modifying drugs within 6 months of vaccination. (For corticosteroids, this will mean prednisone, or equivalent, greater than or equal to 0.5 mg/kg/day. Inhaled and topical steroids are allowed.)
- Administration of a vaccine not foreseen by the study protocol during the period starting from 30 days prior to first dose to 30 day after the third dose visit.
- Administration of immunoglobulins or any blood products up to 30 days after the last dose of vaccine.

## Contraindications to Vaccination

**The occurrence, in any eligible study subject, of any of the events enumerated in Section 7.9.6 (“Dose Discontinuation in Individual Subjects”) represents a contra-indication to further immunization of that subject. However, the parent(s) of such a subject will be encouraged to continue participation in the safety and immunogenicity evaluation for the duration of the study.**

## Indications for Deferral of Vaccination

**The following adverse events constitute grounds for deferral of vaccine administration at a given point in time; if any one of these adverse events occurs at the time scheduled for vaccination, the subject may be vaccinated at a later date, within the allowable time interval specified in the protocol (see Section 8.2), or may be permanently discontinued from further vaccination at the discretion of the investigator (the only withdrawals from follow-up, i.e., from the study proper, would be for withdrawal of consent). The subject must be followed until resolution of the event as with any adverse event (see Section 11). If the subject is discontinued from further vaccination, the parent(s) will be encouraged to keep the subject in the safety and immunogenicity evaluation for the duration of the study.**

- Axillary temperature > 37.5 °C or evidence of clinical malaria (see Section 8.3.1) at the time of vaccination will warrant deferral of immunization until fever and symptoms resolve.
- Any other condition that in the opinion of the investigator poses a threat to the individual if immunized or that may complicate interpretation of the safety of the vaccine following immunization.

Such subjects will be followed daily in the clinic until the symptoms resolve or the window for immunization expires. No further vaccination will be performed if the subject does not recover (axillary temperature < 37.5°C or lack of symptoms, as appropriate) within 7 days of the originally scheduled vaccination date. The subject, however, will continue to be followed for safety and immunogenicity. **If the subject meets any of the above criteria for deferral on the day of first immunization the PI may elect to exclude the subject from further participation in the study.**

## Safety Plan

### **Definitions**

“Discontinuation of vaccination” is the temporary or permanent withholding of further vaccinations from all or some study groups in the trial.

“Dose escalation” refers to the decision to progress from 1/5 dose to ½ dose or from ½ dose to full dose. This progression is from one cohort (dose level) to another.

“Administration of consecutive doses” refers to the progression from the 1^st^ dose to the 2^nd^ dose, and from the 2^nd^ dose to the 3^rd^ dose. This progression is from one monthly inoculation to the next in the subjects of a given cohort.

“Dose limiting toxicities” refer to AEs that preclude further dose escalation or administration of consecutive doses.

### **7.9.2 Criteria for Discontinuation of Vaccination**

If any of the following SAEs occur, discontinuation of vaccination will be invoked and the events will be reported as described in Section 11.5.

1. Death in any subject in which the cause of death is judged to be possibly, probably or definitely related to study vaccine
2. The occurrence in any subject of an anaphylactic reaction to study vaccine
3. The occurrence in any subject of a life-threatening SAE whose causal relationship to vaccination is judged to be probable or definite
4. The occurrence of one or more non-life-threatening SAEs whose causal relationship to vaccination is judged to be definite
5. The occurrence, in one or more subjects, of Grade 4 laboratory abnormalities (See Table 5, “General Toxicity Table”), judged to be probably or definitely related to receipt of study vaccine

The following dose limiting toxicities will preclude dose escalation to the next higher dose level or administration of consecutive doses within a dose level (See Table 5):

1. In 4 (10%) or more children within a cohort, occurrence of Grade 3 or greater solicited systemic (not local) AEs judged probably or definitely related to the study vaccine, , , or
2. In 4 (10%) or more children within a cohort, occurrence of Grade 2 or greater unsolicited AEs judged probably or definitely related to the study vaccine, or
3. In 4 (10%) or more children within a cohort, occurrence of laboratory studies indicating Grade 2 or greater toxicities that are judged to be probably or definitely related to the study vaccine.

### **7.9.3 Safety Review Prior to Dose Escalation**

Following the 7 day period after immunization (and prior to a dose escalation) the LMM will review all available information regarding solicited and unsolicited AEs and SAEs. As there are 6 dose escalations (Doses 1b, 1c, 2b, 2c, 3b, and 3c), there will be 6 such reviews by the LMM. (See Section 8.1.2: ‘Vaccination Process’ and Appendix B [Section 17.2.5: ‘Specific Schedule of Events’]).

Dose escalation will not proceed until all of the following four events have occurred:

1. All study subjects in a given cohort (dose level) have received an injection of study vaccine, and
2. All such subjects have been followed for at least 7 days after the injection, and
3. The LMM has reviewed the available safety data (and has had the opportunity to discuss the data with the DSMB, if he feels it necessary), and recommends further dose escalation and
4. The PI has determined that none of the SAEs or dose limiting toxicities outlined in Section 7.9.2 have occurred.

An additional requirement obtains prior to the first two dose escalations (i.e., prior to Doses 1b and 1c, during the first month of injections). This is a requirement that the DSMB will also review the LMM safety data report (number 3 above) and must concur on the decision to dose escalate, and convey this directive to the PI in writing. For these first two dose escalations, therefore, concurrence to proceed must be forthcoming from both the LMM and the DSMB. Courtesy copies of these reports will also be sent to GSK Biologicals, MVI and SCI. The DSMB is not required to respond to data reports prior to subsequent dose escalations (Doses 2b, 2c, 3b or 3c), although it may do so at its own discretion.

### **7.9.4 Safety Review Prior to Administration of Consecutive Doses**

Reports from the PI to the LMM detailing all AEs and SAEs for the 7 day, post-dose observation period (as in Section 7.9.3), will also be provided to the DSMB, MVI, GSK Biologicals, and SCI following the administration of each of the three consecutive doses (i.e., the first report is due after the first doses [1/5 dose, ½ dose and full dose] have been administered, the second and third reports are provided after doses 2 and 3, respectively).

Administration of consecutive doses (Doses 1b, 1c, 2a, 2b, 2c, 3a, 3b, and 3c) will not proceed until all of the following four events have occurred:

1. All study subjects in a given cohort (dose level) have received an injection of study vaccine, and
2. All such subjects have been followed for at least 7 days after the injection, and
3. The LMM has reviewed the available safety data (and has had the opportunity to discuss the data with the DSMB, if he feels it necessary), and recommends further consecutive doses and
4. The PI has determined that none of the SAEs or dose limiting toxicities outlined in Section 7.9.2 have occurred.

The cumulative total of data safety reports to the LMM required per Sections 7.9.3 and 7.9.4, will be nine, one after each of the nine inoculations. These reports, and who will receive them, are summarized in Table 4.

**Table 4: Summary of 7-day post vaccination AE reports**

| Dose | Report must go to | | |
| --- | --- | --- | --- |
|  | LMM | DSMB | GSK, MVI, SCI |
| 1a | * | * | * |
| 1b | * | * | * |
| 1c | * | * | * |
| 2a | * |  |  |
| 2b | * |  |  |
| 2c | * | * | * |
| 3a | * |  |  |
| 3b | * |  |  |
| 3c | * | * | * |

NB: Upon review of all reports, the LMM must concur with the PI on proceeding with subsequent doses; The DSMB is required to concur only upon the reports for Doses 1a and 1b.

After the “Follow-up Period for Unsolicited AEs” (Day 120), a comprehensive Interim Safety Data Report will be compiled by SCI (See Sections 8.1.5 and 13.6) and submitted to the DSMB by early October 2003.

### **7.9.5 Procedures for Resumption of Dosing Following Discontinuation of Vaccination, Dose Escalation or Administration of Consecutive Doses**

Within 5 working days of discontinuation of further vaccinations, the DSMB Chairperson will convene a meeting (via teleconference, videoconference, face-to-face or a combination of these) to include the following 5 deliberating participants: the PI, the LMM, GSK Biologicals, MVI and the sponsor, to review and discuss the safety data and events leading to the vaccination discontinuation. (Other “observing” or consultative participants, e.g., SCI, USAID, etc, may also attend at the Chairperson’s pleasure.) At least 3 days prior to this meeting, the DSMB will disseminate copies of all relevant safety data to all meeting participants. The Chairperson of the DSMB will also chair this meeting.

There must be a unanimous decision by all meeting participants to re-start the vaccine administrations, either in all study subjects or in specific cohorts. If not, further vaccinations will not commence (again, either in all study subjects or in specific cohorts.) Meeting participants may request additional information prior to making a decision on re-starting or terminating vaccinations, in which case, the DSMB Chairperson will organize a subsequent meeting within 3 working days after the meeting participants have received the requested additional information.

The DSMB (or its designee) will produce and distribute minutes of any such meetings to participants within 5 working days and copies will be maintained at in the study site and with the study sponsor. In the event of either a re-start of vaccine administrations (after a temporary suspension) or a permanent vaccine discontinuation (in a cohort or in all study subjects), the PI will report this decision to all participating IRBs and ERCs within 5 working days.

The decision for *permanent discontinuation* of further vaccinations (in all study subjects or in specific cohorts) will take the final form of a formal recommendation by the DSMB to the sponsor (i.e., the Office of the Surgeon General, U.S. Army; POC: Dr. Charles English, USAMMDA, Ft Detrick, MD, USA).

### **7.9.6 Dose Discontinuation in Individual Subjects**

Reasons for discontinuation of study vaccine administration in an individual subject include, but are not limited to, the following:

1. Anaphylactic reaction following administration of study vaccine
2. Significant (Grade 3 or 4) reactions following administration of study vaccine (including severe pain, severe swelling, severe headache, persistent high fever or other severe systemic or local reactions) that, in the opinion of the investigator or sponsor, preclude further administration of study vaccine
3. The occurrence of an SAE (See Section 11.5.1) whose relationship to the study vaccine is judged to be possible or probable
4. Receipt of any investigative drug, investigative vaccine or immunosuppressive medication (inhaled or topical corticosteroids are allowed) within 28 days of study vaccine
5. The occurrence of other medical conditions, unrelated to study vaccine, which, in the opinion of the PI, contraindicate administration of study vaccine
6. Moderate or severe unsolicited AEs (clinical events or laboratory tests) that are indicative of new and significant end organ dysfunction

**Table 5: General Toxicity Table** (Modified from the Pediatric ACTG Toxicity Tables; See Ref. 43)

| **Event** | **Grade I** | | **Grade 2** | | **Grade 3** | | | **Grade 4** | |
| --- | --- | --- | --- | --- | --- | --- | --- | --- | --- |
| **Laboratory Values** | | | | | | | | | |
|  | Conventional  Units | SI Units | Conventional Units | SI Units | Conventional Units | SI Units | | Conventional Units | SI Units |
| Hemoglobin | 7.0-7.9 gm/dL | 4.4-4.9 mmol/L | 6.0-6.9 gm/dL | 3.8-4.39 mmol/L | <6.0 gm/dL | <3.8 mmol/L | | CHF 2^o^ to anemia | CHF 2^o^ to anemia |
| Leukocytes | 3.0-3.9 10^3^  per mm^3^ | 3.0-3.9 10^9^/L | 2.0-2.9 10^3^  per mm^3^ | 2.9-2.0 10^9^/L | 1.0-1.9 10^3^  per mm^3^ | 1.9-1.0 10^9^/L | | <1.0 10^3^  per mm^3^ | <1.0 10^9^/L |
| Platelets | 90,000-109,999 per mm^3^ | 90.0-109.9 10^9^/L | 75,000-89,999  per mm^3^ | 75.0-89.9 10^9^/L | 50,000-74,999 per mm^3^ | 50.0-74.9 10^9^/L | | <50,000 per mm^3^ | <50.0 10^9^/L |
| Creatinine | 1.1-1.9 mg/dL | 92-176 μmol/L | 2.0-3.9 mg/dL | 177-353 μmol/L | 4.0-5.9 mg/dL | 354-530 μmol/L | | >6.0 mg/dL | >530 μmol/L |
| ALT | 45-60 IU/l | 45-60 IU/l | 61-105 IU/l | 61-105 IU/l | 106-147 IU/l | 106-147 IU/l | | > 148 IU/l | > 148 IU/l |
| **Systemic Events** | | | | | | | | | |
| Fever | 37.5-38.0^o^C | | 38.1-39.0^o^C | | 39.1-40.5^o^C | | >40.5^o^ persistent >48 hours | | |
| Loss of Appetite | Eating less than usual; No effect of normal activity | | Eating less than usual; Interferes with normal activity | | Not eating at all | | Not eating at all; Requires supplemental intake | | |
| Drowsiness | Drowsiness easily tolerated | | Drowsiness that interferes with normal activity | | Drowsiness that prevents normal activity | | Drowsiness eventuating in seizures or coma | | |
| Irritability/  fussiness | Crying more than usual; No effect on normal activity | | Crying more than usual; Interferes with normal activity | | Crying that cannot be comfort-ed; Prevents normal activity | | Irritability/fussiness eventuat-ing in seizures or coma | | |
|  |  | |  | |  | |  | | |
| **Local Events** | | | | | | | | | |
|  | | | | | | | | | |
| Injection site pain or necrosis | Minor reaction to touch | | Cries/protests on touch | | Cries when limb is moved; Spontaneously painful | | Necrosis at the site of injection (With or without pain) | | |
| Injection site swelling | < 5 mm | | 5-20 mm | | 21-35 mm | | > 35 mm | | |

### **7.9.7 Safety Precautions for Study Personnel**

Laboratory safety and blood-borne precautions for all USAMRU-K laboratories are delineated in the following USAMRU-K SOPs:

1. Lab Safety SOP #1: “Blood-borne and Infectious Control SOP”
2. Lab Safety SOP #2: “Hazard Communication and Chemical Hygiene SOP”
3. Lab Safety SOP #1: “Laboratory Safety SOP”
4. Phlebotomy SOP #1: “Phlebotomy Training and Emergency SOP”
5. Phlebotomy SOP #2: “Finger-stick SOP”

# CONDUCT OF THE STUDY

## General Study Aspects

### Screening and enrollment process

(See also SSP No. SM-01: Recruitment & Informed Consent Process, and SSP No. SM-02: Screening Day Procedures.)

Recruitment and screening will be incremental until the desired number of subjects are enrolled. Non-coercive means of recruitment will be used according to existing U.S. Army regulations (viz., AR 70-25 and AR 40-38). Community leaders (assistant chiefs and local village elders) will be formally briefed in their own language on the nature and purpose of the study. They will be allowed to freely ask questions of the PI. Parents of prospective subjects will view a videotaped briefing on the nature and purpose of the study and will then receive both oral and written explanations of the study, after which written informed consent will be obtained from at least one parent per subject. No questions relating to health will be asked of parents or children prior to consenting. So that illiteracy will be no bar to informed consent, all such briefings and explanations will be in the parents’ own language. All screening tests (see below) will be performed after only parental informed consent has been obtained.

A source document file will be prepared by the investigator at screening. These forms will be labeled with the subject's initials and a subject identification (SID) number, and will contain information about the subject's date of birth and medical history, date of screening visit, whether the subject was enrolled or not and (where applicable) reasons for exclusion from the study.

Parents of subjects will provide a medical history (with special attention to any history of recurrent infections to suggest immune suppression, previous history of splenectomy, and prior vaccine reactions). Subjects will also undergo physical examination and routine standard laboratory screening tests, which include complete blood count (CBC) and serum chemistry profiles (creatinine, ALT). Subjects will be excluded from participation if they meet any of the exclusion criteria (Section 7.5). Subjects excluded from this study because of significant abnormalities will be referred to the local health center for evaluation. All screening tests will be completed within 35 days prior to entry into the study; laboratory tests may be conducted at other times during the course of the trial if judged necessary for the safety of the subject by the investigators. All subjects randomized into the study will be identified and followed by SID number and by name (full name in source documents, but no name or initials in CRFs). All screening and follow-up diagnostic laboratory studies will be performed at the WRP Kombewa Clinic laboratory. Information gathered during screening (medical history, physical examination, and laboratory analysis) will be recorded in the screening source documents.

The investigator will ensure randomization (See also Section 10.3) of 135 eligible subjects who fulfill all the inclusion criteria and none of the exclusion criteria. Subjects in each cohort will be randomized separately from the other cohorts, at the times of first vaccinations. A picture ID of parent and child will be created and given to each parent. Copies of these pictures will also be kept in the source document file. A Case Report Form (CRF) will be filled in for all subjects enrolled in the study. All data collected during the study will be recorded on source documents and transcribed into the CRF by a transcription team trained in this process.

### Vaccination Process

On the days of each subsequent vaccination, criteria for continued eligibility will again be reviewed and verified. A history-directed physical examination will be done and axillary temperature, blood pressure, pulse and baseline gen-eral symptom history will be recorded. Venous blood will be collected for laboratory analysis as detailed in Section 9.2.1.

After the identity of the subject is checked (by comparing the SID number and photographs in source document with the parent/subject’s photo ID), subjects will be vaccinated by intra-muscular injection into the right or left anterolateral thigh muscles, unless a compelling reason for an alternate injection site is evident (See also SSP No. SM-04: Vaccination Day Procedures). All vaccinations will be done in a fashion that maintains double blinding (See Section 10.5.1). Every effort will be made to vaccinate all 45 children in a given cohort on a given vaccination day.

Although all the foregoing dates are provisional (circumstances may require delays of up to 5 weeks), vaccinations will occur on or about Study Days 0, 14, and 28 (Dose 1); 29, 42, and 56 (Dose 2); and 57, 70, and 84 (Dose 3). The dose-by-dose scheme is as follows:

Dose 1/Study Days 0, 14 & 28: The first 45 subjects to be vaccinated (Dose 1a) will receive either 1/5^th^ dose = 10 ug of FMP-1 (10 subjects in each of 3 age groups = 30) or Imovax® (5 subjects in each of 3 age groups = 15). Grand total = 45.

Two weeks later (Study Day 14), or up to 5 weeks later if temporary discontinuation is deemed necessary, a second 45 subjects will be vaccinated (Dose 1b) with either ½ dose = 25 ug of FMP-1 (10 subjects in each of 3 age groups = 30) or Imovax® (5 subjects in each of 3 age groups = 15). Grand total = 45.

Two weeks later (Study Day 28), or up to 5 weeks later if temporary discontinuation is deemed necessary, a third 45 subjects will be vaccinated (Dose 1c) with either full dose = 50 ug of FMP-1 (10 subjects in each of 3 age groups = 30) or Imovax® (5 subjects in each of 3 age groups = 15). Grand total = 45. At this point (after Dose 1c is administered) all 135 study subjects will have been recruited and vaccinated once.

(2) Dose 2/Study Days 29, 42 & 56: The first 45 subjects to be vaccinated a second time (Dose 2a) will receive either 1/5^th^ dose = 10 ug of FMP-1 (10 subjects in each of 3 age groups = 30) or Imovax® (5 subjects in each of 3 age groups = 15) on Study Day 29. Grand total = 45.

Two weeks later (Study Day 42), or up to 5 weeks later if temporary discontinuation is deemed necessary, a second 45 subjects will be vaccinated (Dose 2b) with either ½ dose = 25 ug of FMP-1 (10 subjects in each of 3 age groups = 30) or Imovax® (5 subjects in each of 3 age groups = 15). Grand total = 45.

Two weeks later (Study Day 56), or up to 5 weeks later if temporary discontinuation is deemed necessary) a third 45 subjects will be vaccinated (Dose 2c) with either full dose = 50 ug of FMP-1 (10 subjects in each of 3 age groups = 30) or Imovax® (5 subjects in each of 3 age groups = 15). Grand total = 45. At this point (after Dose 2c is administered) all 135 study subjects will have been vaccinated twice.

(3) Dose 3/Study Days 57, 70 & 84: The first 45 subjects to be vaccinated a third time (Dose 3a) will receive either 1/5^th^ dose = 10 ug of FMP-1 (10 subjects in each of 3 age groups = 30) or Imovax® (5 subjects in each of 3 age groups = 15) on Study Day 57. Grand total = 45.

Two weeks later (Study Day 70), or up to 5 weeks later if temporary discontinuation is deemed necessary, a second 45 subjects will be vaccinated (Dose 3b) with either ½ dose = 25 ug of FMP-1 (10 subjects in each of 3 age groups = 30) or Imovax® (5 subjects in each of 3 age groups = 15). Grand total = 45.

Two weeks later (Study Day 84), or up to 5 weeks later if temporary discontinuation is deemed necessary) a third 45 subjects will be vaccinated (Dose 3c) with either full dose = 50 ug of FMP-1 (10 subjects in each of 3 age groups = 30) or Imovax® (5 subjects in each of 3 age groups = 15). Grand total = 45. At this point (after Dose 3c is administered) all 135 study subjects will have been vaccinated thrice.

NB: All “a” doses are the 1/5^th^ dose = 10ug dose (Cohort A); all “b” doses are the ½ dose = 25 ug dose (Cohort B); all “c” doses are the full dose = 50 ug dose (Cohort C).

**Table 6: MAL-031 Timelines for Vaccinating each Cohort (Dosage Group)**

|  | Apr 2003 | May | June | July | Aug + 5 mo |
| --- | --- | --- | --- | --- | --- |
| Cohort A | **1a**  Screening | **2a** | **3a** |  | SAE Follow-Up  (Immunogenicity F/U: Aug + 8 mo) |
| Cohort B |  | **1b** | **2b** | **3b** |  |
| Cohort C |  | **1c** | **2c** | **3c** |  |

### Post-immunization Evaluation Procedures

Immediately after immunization, each subject will proceed to the next station for assessment of local and systemic reactions. Each subject will be observed for 60 minutes post-vaccination or longer if necessary. Signs and symptoms (pain, swelling, fever [defined as axillary temperature ≥ 37.5°C], drowsiness, loss of appetite, and irritability/ fussiness) will be solicited from subject and parent and then recorded in the source document by an evaluator according to adverse events recording procedures as outlined in Section 11.

Subjects will complete a one week follow-up after each vaccination (5 visits: day of vaccination, plus days 1, 2, 3 and 7 post-vaccination) at the WRP Kombewa Clinic. All AEs will be followed until resolution (see Section 11). If any solicited signs or symptoms persist beyond the first four days post-vaccination, the subject will be followed daily until resolution of the adverse event. Unsolicited adverse events will be followed as deemed appropriate by the investigator. At each visit, the subjects will be evaluated by soliciting post vaccination signs and symptoms from the parent and by examination of the injection site. A full physical exam will be performed on Day 7 (Visit 5). Scheduled clinic visits will also be conducted on post-vaccination days 14 and 30 (the latter may coincide with the subsequent vaccination day visit). Every effort will be made to insure parental and subject compliance with visits. However, if a parent and subject do not appear for a scheduled clinic visit, a clinician or designee (e.g., field worker) will attempt to contact them on the same day. The staff member who contacts the subject will measure vital signs and collect information on any solicited or unsolicited symptoms for the previous 24 hours. If the clinician determines that the subject has experienced a serious adverse event (SAE), appropriate measures will be taken to notify the Local Medical Monitor (LMM), the DSMB, the IRBs and sponsor (See Section 11.5.2).

### Field Workers: Activities & Competencies

Field workers will undergo a 2-3-week training period during which they will be taught basic study procedures such as taking vital signs, preparing blood smears and additional didactic information regarding malaria and other general health concerns. At the end of this period they will be tested on their knowledge and skills. Only those who satisfactorily pass the test will participate in the study.

Throughout the study period and monthly after the third dose of vaccine, field workers will assist in tracking the location of parents/subjects and in reminding parents of scheduled visits.

Field worker supervisors will visit a sample of subjects periodically to insure that field workers are indeed tracking and collecting the required information (See SOP No. 101.08, v2: Fieldworker Evaluation).

### Long-term Follow-up Period

Study day 120, marks the beginning of a 4-month “Post-vaccination Safety Surveillance Period for SAEs” (Day 120 ± 14 to Day 240 ± 14), which is in turn followed by a 4-month “Long-term Follow-up Period” (Day 240 ± 14 to Day 364 ± 14). Subjects are to be followed for SAEs only through Study day 240 (or Study month 8, i.e., 6 months after last dose). Malaria episodes occurring after this period are not defined as AEs or SAEs and are not reported as such. However, during the “Long-term Follow-up Period”, immune responses and malaria episodes will be documented (See Table 7).

After the end of the “Follow-up Period for Unsolicited AEs” (Day 120), available data will be compiled into an Interim Safety Data Report (statistical analysis) and submitted to the DSMB in early October 2003 (around Day 157). However, the PI and field team members will remain blinded to all results until 30 days after the 30 day post-vaccination follow-up visit for Cohort C’s third vaccine dose; unblinding of “by individual” study results will not occur until the end of the “Post-vaccination Safety Surveillance Period” (Day 240; See Section 13.6 for fuller details).

**Table 7: MAL-031 Follow-up (F/U) & Surveillance Periods**

| **Month** | **1** | | **2** | **3** | **4** | | | **5** | **6** | **7** | **8** | **9** | | **10** | **11** | **12** |
| --- | --- | --- | --- | --- | --- | --- | --- | --- | --- | --- | --- | --- | --- | --- | --- | --- |
| Study Day | 0-30 | | 31-60 | 61-90 | 91-120 | | | 121-150 | 151-180 | 181-210 | 211-240 | 241-270 | | 271-300 | 301-330 | 331-364 |
|  | V  V  V | | V  V  V | V  V  V |  | | |  |  |  |  |  | |  |  |  |
|  | F/U Period for Unsolicited AEs | | | | |  | |  |  |  |  |  | |  |  |  |
|  | F/U Period for SAEs | | | | | | | | | | |  | |  |  |  |
|  |  |  | |  |  | | Post-vacc Safety Surveillance Period for SAEs | | | | |  |  | |  |  |
|  |  |  | |  |  | | |  |  |  |  | Long-term F/U Period (for Malaria & Immunogenicity) | | | | |
|  |  |  | |  |  | | | Monthly Field Workers Visits | | | | | | | | |
|  |  |  | |  |  | | |  |  | Quarterly WRP Kombewa Clinic Visits | | | | | | |

V = 7 day F/U for Solicited AEs following each vaccination (All cohorts shown)

“Study days” presented for Cohort A only

## Detailed Description of Study Stages & Visits

This study will be performed according to the following procedures. Each cohort (A, B or C) will be scheduled independently as indicated here. For a day-by-day schedule for all cohorts see Section 17.2.5: Specific Daily Schedule of Events.

**Days –35 to -1** **Screening & Enrollment of Subjects**

***Visit 1***

- Written Informed Parental Consent
- Assignment of Subject Identification (SID) number
- Provision of medical history by subject’s parent
- Health assessment and history directed physical examination (includes physical examination of all major body systems: ENT, pulmonary, cardiovascular, musculoskeletal, central nervous, renal, gastrointestinal and skin). Abnormal findings will be recorded on source documents.
- Check of all inclusion and exclusion criteria.
- Check weight
- Collect 2 ml venous blood sample for measurement of:
- Complete blood count (CBC),
- Chemistry profiles (Creatinine, ALT)
- Provide each parent with SID card containing subject’s unique study number and parent/subject photograph. The photograph will be taken only of eligible subjects who agree to participate and have been consented. It may be taken on any day within this period prior to the first vaccination.

**Day 0 Vaccination 1 (Dose 1a, 1b, or 1c)**

***Visit 2***

Before vaccination:

- Check subject’s SID card to confirm identity
- Review screening laboratory test results
- Review inclusion/exclusion criteria and check of contraindications/precautions
- Randomization
- Record any parental or subject complaints, symptom-directed physical examination and examination of the immunization leg for any abnormalities
- Record vital signs (axillary temperature [must be <37.5C], blood pressure, pulse)
- Record baseline data for solicited general symptoms.
- Check weight
- Collect 2 ml of whole venous blood for the measurement of:
- CBC, creatinine, ALT
- Serum for anti-MSP-1 antibody titer (store at ≤–50°C)
- Confirm that the subject’s randomization number and SID number agree with label on syringe
- Administer study vaccine dose 1; record date and time of injection.

After vaccination:

- Observe for 60 minutes.
- Record blood pressure, pulse, axillary temperature (at start and end of 60 minute observation period)
- Record site of injection examination
- Record solicited and unsolicited AEs
- Instruct parent(s) to return with subject to WRP Kombewa Clinic immediately should subject manifest any sign or symptom perceived as serious.

**Days 1-3 Daily Post- vaccination 1 follow-up visits**

***Visit 3-5***

- Record vital signs (temperature, blood pressure, pulse)
- Examine site of injection
- Record solicited and unsolicited AEs
- Symptom-directed general physical examination
- Check concomitant medications

**Day 7 ± 3 days Day 7 Post-vaccination 1 follow-up visit**

***Visit 6***

- Record vital signs (temperature, blood pressure, pulse)
- Examine site of injection
- Record solicited and unsolicited AEs (of previous 4 days)
- Complete physical examination
- Check concomitant medications

**Days 14 ± 3 days Day 14 Post vaccination 1 follow-up visit**

***Visit 7***

- Record vital signs (temperature, blood pressure, pulse)
- Brief medical history
- History-directed physical examination
- Record any unsolicited adverse events occurring after the last vaccine dose.
- Check weight
- Check concomitant medications
- Collect 2 ml of blood for determination of:
- Serum for anti-MSP-1 antibodies (Store at ≤ -50°C)
- CBC, creatinine, ALT

**Day 30 ± 7 days Vaccination 2 (Dose 2a, 2b, or 2c)**

***Visit 8***

Before vaccination:

- Check of subject’s SID number to confirm identity
- History-directed physical examination and examination of the site of injection for any abnormalities
- Check contraindications/precautions
- Record vital signs (axillary temperature, blood pressure, pulse)
- Review medical history and record any unsolicited adverse events occurring since last visit.
- Record baseline data for solicited general symptoms
- Check weight
- Collect 2 ml of blood for measurement of:
- Serum for anti-MSP-1 antibodies (Store at ≤ -50°C)
- CBC, ALT, creatinine
- Confirm that the subject’s randomization number and SID number agree with label on syringe
- Administer study vaccine dose 2, record date and time of injection.

After vaccination:

- Observe subject for at least 60 minutes following vaccination.
- Record blood pressure, pulse, and axillary temperature (at start and end of 60 minute observation period)***.***
- Record solicited and unsolicited AEs
- Examine site of injection
- Instruct parent(s) to return with subject to WRP Kombewa Clinic immediately should subject manifest any sign or symptom perceived as serious.

**Days 31-33 ± 7 Daily Post-vaccination 2 follow-up visits**

***Visit 9-11***

- Record vital signs (axillary temperature, blood pressure, pulse)
- Examine site of injection
- Record solicited and unsolicited AEs
- Symptom-directed physical examination
- Check concomitant medications

**Day 37 ± 7 Day 7 Post-vaccination 2 follow-up visit**

***Visit 12***

- Record vital signs (axillary temperature, blood pressure, pulse)
- Examine site of injection
- Record solicited and unsolicited AEs (of previous 4 days)
- Complete physical examination
- Check concomitant medications

**Day Visit 44 ± 7 Day 14 Post-vaccination 2 follow-up visit**

***Visit 13***

- Record vital signs (temperature, blood pressure, pulse)
- Brief medical history
- History-directed physical examination
- Record any unsolicited adverse events occurring after the last vaccine dose
- Check weight
- Check concomitant medications
- Collect 2 ml of blood for determination of:
- Serum for anti-MSP-1 antibodies (Store at ≤ -50°C)

- CBC, creatinine, ALT

**Day 60 ± 7 days Vaccination 3 (Dose 3a, 3b, or 3c)**

***Visit 14***

Before vaccination:

- Check subject’s SID number to confirm identity
- History-directed physical examination
- Check contraindications/precautions
- Record vital signs (axillary temperature, blood pressure, pulse)
- Review medical history and record any unsolicited adverse events occurring since last visit.
- Record baseline data for solicited general symptoms
- Check weight
- Collect 2 ml of blood for measurement of:
- Serum for anti-MSP-1 antibodies (Store at ≤ -50°C)
- CBC, ALT, creatinine
- Confirm that the subject’s randomization number and SID number agree with label on syringe
- Administer study vaccine Dose 3; record date and time of injection.

After vaccination:

- Observe subject for at least 60 minutes following vaccination
- Record blood pressure, pulse, and axillary temperature (at start and end of 60 minute observation period)
- Record solicited and unsolicited adverse events
- Examine site of injection
- Instruct parent(s) to return with subject to WRP Kombewa Clinic immediately should subject manifest any sign or symptom perceived as serious

**Days 61-63 ± 7 Daily Post-vaccination 3 follow-up visits**

***Visit 15-17***

- Record vital signs (axillary temperature, blood pressure, pulse)
- Examine site of injection
- Record solicited and unsolicited AEs
- Symptom-directed physical examination
- Check concomitant medications

**Day 67 ± 7 Day 7 Post-vaccination 3 follow-up visit**

***Visit 18***

- Record vital signs (axillary temperature, blood pressure, pulse)
- Examine site of injection
- Record solicited and unsolicited AEs (of previous 4 days)
- Complete physical examination
- Check concomitant medications

**Day 74 ± 7 Day 14 Post-vaccination 3 follow-up**

***Visit 19***

- Record vital signs (temperature, blood pressure, pulse)
- Brief medical history
- History-directed physical examination
- Record any unsolicited adverse events occurring after the last vaccine dose
- Check weight
- Check concomitant medications
- Collect 2 ml of blood for determination of:
- Serum for anti-MSP-1 antibodies (Store at ≤ -50°C)
- CBC, creatinine, ALT

**Day 90 ± 10 Day 30 Post-vaccination 3 follow-up**

***Visit 20***

- Record vital signs (temperature, blood pressure, pulse)
- Brief medical history
- History-directed physical examination
- Record any unsolicited adverse events occurring after the last vaccine dose
- Check weight
- Check concomitant medications
- Collect 2 ml of blood for determination of:
- Serum for anti-MSP-1 antibodies (Store at ≤ -50°C)
- CBC, creatinine, ALT

**Day 120 ± 14 to Day 240 ± 14 Post-vaccination Safety Surveillance Period for SAEs**

- Children will be followed for SAEs through Study day 240
- Beginning on Study day 120 (for Cohort A; appropriate subsequent days for Cohorts B and C), parents/subjects will be visited monthly ± 14 days by field workers to remind them of appointments and to confirm their location.
- Parents/subjects will continue to attend the WRP Kombewa Clinic whenever they are sick.

**Day 240 ± 14 to 364 ± 14 Long Term Follow-up Period**

*Visit 21-23*

- Beginning on Study day 180 (for Cohort A; appropriate subsequent days for Cohorts B and C), parents/subjects will be asked to return to the WRP Kombewa clinic every 3 months ± 14 days when 2 ml of blood will be obtained for hemoglobin and serum anti-MSP-1 antibody measurement and history directed physical. This quarterly visit will substitute for a monthly field worker visit.
- Passive follow-up for malaria will consist of visits to the WRP Kombewa Clinic whenever the subject is sick. During each visit the subject will be evaluated and a malaria smear will be done whenever symptomatic malaria is suspected based on criteria outlined in Section 8.3.1.

**Table 8: Intervals between Study Visits**

| **Interval** | **Size of interval** |
| --- | --- |
| ***(Visit 2→Visit 6)*** | ***7 ± 3 days*** |
| ***(Visit 2→Visit 7)*** | ***14 ± 3 days*** |
| ***(Visit 2→Visit 8)*** | ***30 ± 7 days*** |
| ***(Visit 8→Visit 12)*** | ***7 ± 3 days*** |
| ***(Visit 8→Visit 13)*** | ***14 ± 3 days*** |
| ***(Visit 8→Visit 14)*** | ***30 ± 7 days*** |
| ***(Visit 14→Visit 18)*** | ***7 ± 3 days*** |
| ***(Visit 14→Visit 19)*** | ***14 ± 3 days*** |
| ***(Visit 12→Visit 20)*** | ***30 ± 7 days*** |

**Table 9: Schedule^a^** of Routine Subject Evaluations for Cohort A

| Visit | 1 | 2 | 3-5 | 6 | 7 | 8 | 9-11 | 12 | 13 | 14 | 15-17 | 18 | 19 | 20 | 21-23 |
| --- | --- | --- | --- | --- | --- | --- | --- | --- | --- | --- | --- | --- | --- | --- | --- |
| **Study Day** |  | **0** | **1-3** | **7** | **13** | **29** | **30,31,34** | **36** | **42** | **62** | **63-65** | **69** | **73** | **91** | **120-364** |
| Written Informed Parental Consent | x |  |  |  |  |  |  |  |  |  |  |  |  |  |  |
| Verification of Eligibility Criteria | x | x |  |  |  | x |  |  |  | x |  |  |  |  |  |
| Medical History | x | x |  |  |  | x |  |  |  | x |  |  |  | x |  |
| Physical Examination | x | x |  | x | x | x |  | x | x | x |  | x | x | x | x |
| Weight checks | x | x |  |  | x | x |  |  | x | x |  |  | x | x | x |
| CBC^d^ | X^b^ | x**^c^** |  |  | x | x**^c^** |  |  | x | x**^c^** |  |  | x | x | x |
| Serum Chemistry (ALT, Cr) | X^b^ | x**^c^** |  |  | x | x**^c^** |  |  | x | x**^c^** |  |  | x | x |  |
| Serum for Antibody Responses |  | x**^c^** |  |  | x | x**^c^** |  |  | x | x**^c^** |  |  | x | x | x**^e^** |
| Vital Signs (T, BP, P) | x | x**^f^** | x | x | x | x**^f^** | x | x | x | x**^f^** | x | x | x | x |  |
| Study Vaccine Administration |  | x |  |  |  | x |  |  |  | x |  |  |  |  |  |
| Check contraindications/precautions |  | x |  |  |  | x |  |  |  | x |  |  |  |  |  |
| Assess Adverse Events**^g^** |  | x | x | x | x | x | x | x | x | x | x | x | x | x |  |
| Assess Serious Adverse Events**^h^** |  | x | x | x | x | x | x | x | x | x | x | x | x | x | x**^h^** |
| Concomitant Medications**^i^** |  | x | x | x | x | x | x | x | x | x | x | x | x | x |  |
| Interim History |  |  | x | x | x | x | x | x | x | x | x | x | x |  | x |
| Monthly field worker visits**^j^** |  |  |  |  |  |  |  |  |  |  |  |  |  |  | x |
| Quarterly WRP KC visits |  |  |  |  |  |  |  |  |  |  |  |  |  |  | x |
| Review of Health Status**^k^** |  |  |  |  |  |  |  |  |  |  |  |  |  |  | x |
| Scheduled blood volumes | 2 | 2 | 0 | 0 | 2 | 2 | 0 | 0 | 2 | 2 | 0 | 0 | 2 | 2 | 6 |
| **Cumulative** Blood Vol. (mL) | 2 | 4 | 4 |  | 6 | 8 | 8 |  | 10 | 12 | 12 |  | 14 | 16 | 22 |
| 1. Visit numbers & Study day numbers are the same (with slight variations for the latter) for all cohorts; Days/dates for doses 2 & 3 are for planning purposes & are provisional only. 2. Performed within 35 days prior to randomization. 3. Blood collected just before immunization. 4. Limited to Hgb, WBC, lymphocytes, platelets; Hgb determination every 3 mo from Study days 120 to 364. 5. Serum for MSP-1 antibodies collected every 3 mo from Study day 120 to 364 6. Pre-dose, post dose and 60 min after each dose. 7. Solicited symptoms collected for 4-days and on day 7 after each dose. Unsolicited AEs collected for 30 days after each dose. 8. SAEs collected from 1st injection (Study day 0) through 6 months (180 days) after the last injection (Study day 240). 9. Concomitant medications collected through 30 days after last dose of study vaccine. 10. Monthly field worker visits to check on status of subjects. 11. Record any new onset chronic or acute diseases or other medically significant conditions, unscheduled clinic visits, and any new treatments since previous scheduled study visit. | | | | | | | | | | | | | | | |

## Definition and Management of Symptomatic Malaria

### Definition of Symptomatic Malaria

Because the present study is a safety, not an efficacy, trial, there is no need for a formal case definition of malaria. However, in the event that a subject presents to the WRP Kombewa Clinic with fever, history of fever within 48 hours, or an illness which the attending doctor suspects may be due to malaria infection, a blood slide will be taken to confirm the diagnosis. All subjects with asexual parasitemia will be treated with antimalarials as below.

### Management of Symptomatic Malaria during the Study

At the present time, Kenyan Ministry of Health recommendations (40) for the first-line treatment for symptomatic malaria in children 1 to 4 years old remain 1/2 tablet once of sulfadoxine 500 mg/pyrimethamine 25 mg. However, the treatment of malaria will differ from the MoH *Guidelines* because, currently, parasite strains present in Kenya show significant resistance to both the *Guideline*’s first- and second-line drug options (41, 42). Therefore, artemether/lumefantrine (Coartem®) will be used for treatment of children who acquire uncomplicated malaria during the study. This drug has been shown to be safe and effective in children in Africa (43). Artemether/lumefantrine will be ad-ministered as a 6 dose regimen over 48 hours in accordance with the scheme outlined in Table 10.

**Table 10: Artemether/Lumefantrine (Coartem/Riatem®) Dosing Schedule**

| **Body weight (Kg)** | **No. tablets* at 0, 8, 24, 36, 48 & 60 hrs** |
| --- | --- |
| 5 to 14.9 | 1 |
| 15 to 24.9 | 2 |
| 25 to 35 | 3 |

*Each tablet contains 20 mg of artemether and 120 mg of lumefantrine

In addition to artemether/lumefantrine, and in the unlikely event of treatment failure with that drug combination, the following regimen will also be available at the WRP KC as an alternative for the treatment of uncomplicated malaria:

- *Malarone* Pediatric Tablets (atovaquone 62.5 mg and proguanil Hcl 25 mg) dosed according to the body weight schedule presented in Table 11.

**Table 11: *Malarone* Dosage for Treatment of Acute Uncomplicated Malaria in Pediatric Patients**

| **Weight (kg)** | ***Malarone* Pediatric Tablets**: **Total Daily Dosage** | **Dosage Regimen** |
| --- | --- | --- |
| 11-20 | 250 mg/100 mg | 4 *Malarone* Pediatric Tablets as a single dose daily for 3 consecutive days |
| 21-30 | 500 mg/200 mg | 8 *Malarone* Pediatric Tablets as a single dose daily for 3 consecutive days |
| 31-40 | 750 mg/300 mg | 12 *Malarone* Pediatric Tablets as a single dose daily for 3 consecutive days |
| >40 | 1 g/400 mg | 16 *Malarone* Pediatric Tablets as a single dose daily for 3 consecutive days |

For subjects of < 11 kg body mass, the following regimen will also be available at the WRP KC as an alternative for the treatment of uncomplicated malaria:

- Quinine: Standard oral course of quinine 10 mg/kg t.i.d. for 7-10 days.

Children with manifestations of severe malaria (e.g., parasitemia > 5%, hemo-globin < 6 g/dL, coma, convulsions) will be treated with parenteral quinine, 15 mg/kg loading dose followed by 10 mg/kg, q12 hrs. Quinine will be administered in 5% dextrose. Definitive clinical management will done at the New Nyanza Provincial General Hospital (NNPGH), Kisumu, Kenya. Subjects with uncomplicated malaria and vomiting will be admitted for parenteral anti-malarials.

All study subjects treated for malaria will be followed-up (at the clinic or by field worker home visit) until resolution of symptoms. All treatment courses will be administered under supervision of the treating physician or clinical officer and recorded in the source document and in the medication section of the CRF. Subjects found to have clinical malaria will be treated before any further immunization. Subjects evaluated in this manner will be given the appropriate dose of vaccine if their clinical symptoms resolve within 3 days. If the clinical symptoms do not resolve within 3 days the subject will not be vaccinated. However, he or she will be followed for collection of safety and immunogenicity data.

During the course of evaluation for malaria, other illnesses may be uncovered in the study subjects, such as upper respiratory infections and other minor bacterial or viral infections. The pharmacy at the WRP Kombewa Clinic will provide subjects with common over-the-counter analgesics such as paracetamol as well as antibiotics for the treatment of minor ailments and infections. If further evaluation or treatment is necessary, the subject will be transferred to the NNPGH.

# SAMPLE HANDLING & ANALYSIS

## Overview of Collection Time Points

Blood will be collected from study subjects by venipuncture up to 11 times during the study. The maximum amount of blood requested from any subject for standard collection during the study should not exceed 22 ml. However, additional blood may be obtained as deemed necessary by one of the investigators or clinicians to evaluate any illness or condition.

***Safety***

Tests for CBC, creatinine, ALT, at screening, and approximately at Study days 0, 14, 30, 44, 60, 74, 90, and Hbg every 3 months thereafter will be performed at the WRP Kombewa Clinic.

***Serology***

Separation of serum/plasma from the venous blood will be performed at the WRP Kombewa Clinic and serum samples (approximately 0.5-1.0 ml) will be aliquoted for later use to determine anti-MSP-1 antibody levels at approximately Study days 0, 14, 30, 44, 60, 74, 90 and every 3 months thereafter until the conclusion of the study.

## Handling of Biological Samples Collected by the Investigator

### Instructions for Handling of Serum Samples

1. Collection

Venous whole blood will be collected observing appropriate aseptic conditions. Serum will be collected whenever possible using Vacutainer® tubes with integrated serum separator (e.g. Becton-Dickinson Vacutainer® SST or Corvac® Sherwood Medical) or serum microtainers so as to minimize the risk of hemolysis and to avoid blood cell contamination of the serum when transferring to standard serum tubes. Serum samples will be collected from EDTA or heparinized blood following centrifugation at 2000 g for 3 min.

2. Serum separation

These guidelines aim to ensure high quality serum by minimizing the risk of hemolysis, blood cell contamination of the serum or serum adverse cell toxicity at testing.

- For separation of serum using Vacutainer® tubes, the instructions provided by the manufacturer should be followed.
- Following separation, the serum should be transferred to the appropriate standard tubes using a disposable pipette. The serum should be transferred as gently as possible to avoid blood cell contamination.

• The tube should not be overfilled (max. 3/4 of the total volume) to allow room for expansion upon freezing.

• The tube should be identified by an appropriate cryo-resistant label.

### Labeling

• Standard cryolabels will be used to label each serum or blood sample. In addition to a barcode, each label will contain the KEMRI SSC number, WRAIR protocol number, the SID number, and the date of collection. To ensure adequate blinding, a separate set of ID numbers will be generated for the vials to be used for the anti-MSP-1 assay. A link will be created with the SID numbers and provided to a non-blinded staff member.

• If necessary, any hand-written additions to the labels should be made using indelible ink.

• To ensure optimal attachment of the label, it should be attached to the tube as follows:

- First attach the blank end of the label to the tube

- Wrap the label around the tube so that the opposite end of the label overlaps the blank end ensuring that no written portion is covered

• Labels should not be attached to caps.

• The tubes of serum should be stored in a vertical position at a temperature ≤ -50°C.

## Laboratory Assays

The PI and laboratory officer will maintain detailed SOP’s governing laboratory assays in the WRP Kombewa Clinic laboratory. The following overview summarizes the methods that will be used.

***Hematology/Biochemistry:***

A complete blood count (CBC), renal and liver function tests will be measured at regular intervals throughout the study period. Performance of laboratory reference value checks will be ensured by the PI and lab officer and documentation will be provided to the study monitor before study start.

***Procedure for the Laboratory Diagnosis of Malaria***

If a subject is suspected of having clinical malaria, diagnostic blood smears will be made. (“SOP: Malaria Smear” for details.) Results from each blood smear will be recorded in a smear log sheet, as well as on a “Blood Smear Report” form. All blood smears will be archived in the WRP Laboratory, Kisian, or at the WRP Kombewa Clinic, for future reference.

***Serology (Antibody Responses)***

Serological assays will be performed at the USAMRU-Kenya, for antibody determination. Serum will be collected at indicated time points (see Section 9.1). Blood for analysis of antibody responses will be obtained from each subject and allowed to clot for one hour at room temperature, and serum will be separated and frozen at ≤ -50^o^C until tested. All blood samples will be labeled with the KEMRI SSC number, WRAIR protocol number, the SID number and the date the sample was obtained (See Section 9.2.2 ).

Immunogenicity (antibody levels) will be determined by evaluating antibody (IgG) responses to the *P. falciparum* MSP-1_42_ as measured using standard ELISA methodologies with appropriate capture antigens.

*Optional Immunological Readouts*

Results from these optional tests will not be encoded into the final database but will be analyzed and summarized separately by WRP or WRAIR. Immunofluorescence (IFA) titers against whole merozoites may also be measured in subjects both pre- and post-immunization; standard procedures will be followed. A subject will be considered a seroconverter by IFA if, two weeks after the second dose of vaccine, the subject develops an antibody titer (as measured by the highest IFA titer that gives a positive reaction) that is greater than 4 times that of the pre-immune serum. IFA assessments will be performed in either the Department of Immunology, WRAIR or at WRP Kisian.

Any serum not immediately used in antibody assays will be stored indefinitely at ≤ -50°C and may be used in additional *in vitro* tests which may be useful in optimizing the follow-up of study subjects or in evaluating the immune response to the test vaccine preparation in these subjects. If this assay is not specified in this protocol, approval will be requested from the pertinent IRB(s) should the need for that assay arise.

*Other Studies*

Long-term storage of sera will be undertaken so that other laboratory assays or tests, unanticipated at the present time, may be done in the future. Any such proposal for future testing, however, must be submitted for approval to the KEMRI ERC.

# STUDY VACCINES & VACCINE ADMINISTRATION

## Study Vaccines

### FMP-1 Vaccine

The candidate vaccine to be used has been developed and manufactured by the WRAIR (See Section 4.4). The adjuvant, AS02A is manufactured by GSK Biologicals (See Sections 4.3 to 4.6). The Quality Control Standards and Requirements for each component of the vaccine are described in separate release protocols and the required approvals have been obtained.

**ACTIVE INGREDIENTS**

*FMP-1 Vaccine*

The lot of formulated FMP1 to be used in this trial (#0770) consists of vials with volumes of 600 μl each. This includes the excipients (cryoprotectants) Tween 80 (0.2%) and lactose (3.15%), as well as the FMP1 antigen itself (approximately 63.6 μg of lyophilized protein per 600 μl vial or 106 μg/ml of the formulated vaccine).

*AS02A Adjuvant*

**[Material has been redacted here**; The AS02A adjuvant is a proprietary oil in water emulsion with the immunostimulants monophosphoryl lipid A (MPL;Corixa,Seattle,WA, USA )and *Quillaja saponaria* (QS21; Antigenics,New York,NY,USA ).**]**

*Reconstitution of Vaccine with Adjuvant*

Each vial of FMP-1 vaccine (approximately 63.6 μg) will be reconstituted with 0.6 ml of AS02A adjuvant. Thus, injection of the following volumes of the reconstituted vaccine will result in administration of the indicated doses:

Injection of 0.10 ml will deliver a 10.6 µg dose of FMP-1 (Cohort A).

Injection of 0.25 ml will deliver a 26.5 µg dose of FMP-1 (Cohort B).

Injection of 0.50 ml will deliver a 53.0 µg dose of FMP-1 (Cohort C).

### Imovax® Rabies Vaccine

The rabies vaccine, Imovax® Rabies, manufactured by Aventis Pasteur, SA, is a sterile, stable, freeze-dried suspension of rabies virus prepared from the strain PM-1503-3M, obtained from the Wistar Institute, Philadelphia, PA. Each 1.0 ml dose of reconstitut-ed vaccine contains 100 mg of human albumin, < 150 μg of neomycin sulfate and equal or greater than 2.5 IU of rabies antigen. The potency of the final product is determined by the NIH mouse potency test using the US reference standard. The vaccine is supplied as single dose vials containing lyophilized antigen with 1.0 ml of diluent in pre-filled syringe (sterile water for injection).

## 10.2 Vaccine Dosage & Administration

### Reconstitution of FMP-1 Vaccine

The top of a lyophilized FMP-1 vaccine vial will be disinfected with alcohol swabs and allowed to dry for a few seconds. The contents of one syringe pre-filled with AS02A (approximately 0.6 ml) will be injected using a sterile needle into a vial of lyophilized vaccine. The needle will be discarded and the used syringe retained for accountability purposes. The pellet of FMP-1 will then be dissolved by gently swirling the vial and waiting for about 1 minute to ensure complete dissolution of vial contents before withdrawing 0.1, 0.25 or 0.5 ml (for doses of 10, 25 or 50 µg, respectively) of reconstituted FMP-1 into a sterile syringe with a 1” 23 gauge needle (See also Section 10.5.1 for instructions on concealment of the syringe contents).

### Reconstitution of Imovax® Rabies vaccine

The top a vaccine vial of Imovax® Rabies vaccine will be disinfected with alcohol swabs and allowed to dry for a few seconds. The complete contents of a pre-filled syringe containing diluent (1 ml of water for injection) will be injected into a vial of lyo-philized vaccine. The pellet will then be allowed to dissolve by gently swirling the vial and waiting for 1 minute to ensure complete dissolution of vial contents before withdraw-ing 1.0 ml of the reconstituted rabies vaccine into a sterile syringe with a 1” 23 gauge needle (See also Section 10.5.1 for instructions on concealing the syringe contents). Once the single-dose vial has been penetrated, the withdrawn vaccine will be used promptly and the vial will be retained for accountability purposes.

### Administration of Vaccines

Each 0.1, 0.25 or 0.5ml (for 10, 25 or 50 µg doses, respectively) of FMP-1 and each 1.0 ml (2.5 IU dose) of Imovax® Rabies vaccine will be administered by slow intramuscular injection into the right or left anterolateral thigh muscles, alternately with sequential injections. This is the recommended route and site for Imovax® administration in neonates, infants and young children. In the case of FMP-1, injection will be done immediately after reconstitution. (“Immediately” in this context implies not more than several minutes.)

Intravenous, subcutaneous and intradermal routes of administration will not be used. Aspiration will be performed prior to injection to avoid injection into a blood vessel. If a vessel is inadvertently penetrated, the needle will be withdrawn and reinjected at a slightly different site. As an alternative site of immunization, the same anterolateral thigh muscles (right or left) as previously injected, will be used when injection at the preferred site is contraindicated or not advisable such as in the case of severe pain, infection or if the subject or parent voices a preference to be immunized in the alternative site. A separate syringe and needle will always be used for each subject receiving either vaccine. In no case (whether FMP-1 or Imovax®)will more than one vaccine dose be obtained from a single vial. The vaccines will be visually inspected for particulate matter and discoloration prior to administration if solutions and containers permit. Once the single-dose vial has been penetrated, the withdrawn vaccine will be used promptly, and the used vial will be retained for accountability purposes. The vaccinees will be observed closely for at least 60 minutes, with appropriate medical treatment readily available in the unlikely event of anaphylactic reaction following the administration of the vaccine (See SOP No. 403.03, “Treatment of Anaphylaxis in Children”)

The vaccines will be administered in the WRP Kombewa Clinic under the supervision of physicians skilled in the management of anaphylactic reactions. Subjects will receive the vaccine given by injection into the right or left anterolateral thigh muscles. In order to maintain the study blind, the team of clinicians tasked to administer the vaccine will be different from those involved in the evaluation of subjects following immunization.

## Vaccine Storage

# ALL VACCINES MUST BE STORED IN THE REFRIGERATOR (+ 2°C to + 8°C) AND MUST NOT BE FROZEN. All AS02A SYRINGES should be stored at +2°C to +8°C. Vaccines will be maintained in a refrigerator that has 24 hr temperature recording. A back-up refrigerator and generator will be available in case of breakdown/power failure. The refrigerator that holds the vaccines and the adjuvant will be kept locked and keys to it will only be routinely possessed by the designated drug manager, the clinic pharmacist, or the deputy clinic director for administration. This refrigerator will be used solely for this purpose. Records will be maintained that document receipt, temperature during transit, release for immunization, disposal, or return to the manufacturer for all vaccine vials. Copies of these records will be provided to the sponsor for archiving. (See also SOP No. 701.01: “Vaccine and/or Drug Refrigerator Monitoring and Maintenance”, SOP No. 701.02: “Vaccine and/or Drug Refrigerator Failure”, and SSP No. PM-01: “Vaccine Storage and Packaging for Transport”.)

## Randomization of Treatment Allocation & Age

Subjects in each cohort (dose group) will be randomized separately from the other cohorts, each cohort at the time of its first vaccination. The study will use blocked randomization with stratification for age, but not for gender. It is not expected that this will result in an important imbalance in the gender distribution between the test (N=90) and comparison (N=45) groups. However, subjects in each cohort will be randomized to one of the three age groups. Calculations were performed by SCI based on the most extreme allocations of the comparator vaccine (where the allocation of FMP-1 to comparator is 2:1 and 15 subjects are randomized in each of 3 age categories for each of cohort). Outcomes of concern would be those that randomized no more than 1 subject from a given age group into the comparator vaccine group, for example:

Number receiving the comparator vaccine among

    1 year olds 2 year olds    3 year olds

           15                  0                  0

           14                  1                  0

           12                  0                  3

            0                  9                  6

The calculations indicated that for a given cohort, the chance of one of these outcomes occurring is approximately 2%. Further, the chance of this occurring in at least one of the three cohorts is roughly 6%. Although these chances are not extremely high, they are thought to be high enough to raise concern. Therefore, SCI has recommended that the PI incorporate age stratification into the study design. SCI does not, however, recommend analyzing within these strata because of the very small sample sizes.

The randomization list will contain sequential codes linked to a study vaccine assignment (FMP-1 or rabies vaccine). The codes will be assigned to subjects in the order in which they present to the clinic on the first day of immunization. The only persons at the study site with access to the randomization list are the study drug manager, clinic pharmacist and his assistant(s); therefore these individuals will need to be unblinded. It is essential that these unblinded individuals understand the importance of not revealing the contents to anyone else involved in the study. The LMM will also keep one set of the randomization codes in a sealed envelope in the event that emergency unblinding is necessary.

## Method of Blinding & Breaking the Study Blind

### **Blinding**

The comparator vaccine will be in the same package as received from the manu-facturer and after agitation it will appear white and slightly opaque. The FMP-1 vaccine and the AS02A diluent will be packaged separately. The reconstituted FMP-1 vaccine in the AS02A diluent will have exactly the same milky white appearance as the comparator vaccine. After preparation, the test article and comparison vaccines should have the same appearance. The drug manager, an experienced nurse or a pharmacist, will be responsible for vaccine preparation. Additionally, a drug manager assistant will be assigned to the drug manager to ensure and verify that the proper vaccine and vaccine dose is delivered to each subject. To determine which vaccine each subject will receive, the drug manager will refer to the unique randomization code assigned to that subject (See Section 10.3). The drug manager will check the subject number on the parent/ subject’s photo ID and will make sure that it matches that in the source document and CRF. The drug manager will then refer to a key matching the randomization code given to the subject to the vaccine to be administered. The drug manager assistant will also confirm that the randomization code of the subject matches the vaccine to be given in the key list. The vials will be prepared (Imovax®) or reconstituted (FMP-1), as in Sections 10.2.1 and 10.2.2, and the appropriate volumes will be drawn into syringes. Each syringe will be labeled with a sticker containing the randomization code for the individual and the individual’s SID number. The clinicians or investigators assigned the task of immunizing will be different from those assigned to do post-immunization assessment.

Immunization will be carried out simultaneously in 4 different exam/consultation rooms which are connected to the vaccine preparation room by small, closable service hatches. The hatches will remain closed at all times except to exchange records and the pre-filled syringe for immunization. The vaccine-filled syringes will be handed through this window to the vaccinators. The vaccinators will be medically qualified personnel (e.g. a medical doctor, physician assistant, or nurse) with experience in IM injection of vaccines. For each subject, the SID number, the randomization code from the chart, and the randomization code on the syringe containing the vaccine will be recorded on the source document vaccination form. (For more details, see SSP PM-02, “Vaccine Preparation and Administration”).

### **Breaking the Study Blind**

A subject's study randomization code may be unblinded only for safety purposes. Such procedures are exceptional and should be discussed by the sponsor, the PI, the LMM, and the DSMB. If deemed necessary for reasons such as safety, the LMM or the Drug Manager in Kenya or SCI in the USA will unblind a specific subject without revealing the study blind to the investigators. Any opening of these coded envelopes will be well documented.

The PI and Kenya field team members will remain blinded to all study data until the 30 days after the 30 day post-vaccination follow-up visit for Cohort C’s third dose (provisionally: Study Day 114; thus, this partial unblinding occurs on Study Day 144). Full (“by individual”) study unblinding will occur after the end of the “Post-vaccination Safety Surveillance Period” (Study Day 240; See Section 13.6). In the event that a study code has become known to the PI, the sponsor must be notified immediately by the PI and the reasons for the event documented in the study record.

## Replacement of Unusable Vaccine Doses

In addition to study vaccines, additional single doses of FMP-1, AS02A and Energix-B® will be provided to replace broken or lost doses. All the procedures described in Section 10 still apply.

## Vaccine Accountability

The study vaccines and diluents will be kept in a locked refrigerator at either the WRP Clinic or the Kisian Laboratory both of which have 24 hr temperature recording capability (See SSP No. PM-01: “Vaccine Storage and Packaging for Transport” and Section 10.3 above). Movement of these items into and out of the site will be documented appropriately. In no case (whether FMP-1 or Imovax®) will more than a single vaccine dose be obtained from a single vial. Used vaccine vials, as well as unusable vaccine vials will be retained for vaccine accountability purposes.

## Concurrent Medication/Treatment

At each study visit or other clinical encounter, an investigator will question a subject’s parent about any concurrent medication taken by the subject.

Concurrent medications—including any vaccine other than the study vaccines, and any other medication relevant to the protocol, including any specifically contraindicated—administered during the period starting from one week before each dose and ending one month (maximum 30 days) after must be recorded in the case report form with trade name or generic name of the medication, medical indication, start and end dates.

### **Drugs for Treatment of Anaphylaxis**

Drugs to treat anaphylaxis include epinephrine 1:1,000 (Aqueous), diphen-hydramine, and methylprednisolone, all of which are readily available at the WRP Kombewa Clinic. Epinephrine SQ may be administered to children, 0.01 ml/kg/dose (max single dose, 0.5 ml); diphenhydramine PO, IM or IV may be given 5 mg/kg/24 hrs (max dose, 300 mg/24 hrs); and methylprednisolone IV, may be given as a loading dose of 2 mg/kg/dose X 1 hr and a maintenance dose of 2 mg/kg/24 hrs (given every 6 hrs) as needed to treat anaphylaxis. A kit with supplies necessary for airway management and oxygen will also be available on site. A physician familiar with Pediatric Advanced Life Support (PALS) procedures will be present on-site during immunizations. The WRP Kombewa Clinic guideline is SOP No. 403.03, “Treatment of Anaphylaxis in Children”.

### **Drugs to Treat Malaria**

*Coartem*® (artemther/lumefantrine) will be the first line agent for the treatment of uncomplicated malaria. In addition, both *Malarone*® Pediatric Tablets (atovaquone 62.5 mg and proguanil Hcl 25 mg) and oral and parenteral quinine will be available on site at the WRP Kombewa Clinic as alternatives. These drugs will be used in standard doses and regimens as described in Section 8.3.2.

# ADVERSE EVENTS

The recording of adverse events is an important aspect of study documentation. It is the responsibility of the investigator to document all adverse events according to the detailed guidelines set out below. The subjects’ parent(s) will be instructed to contact the investigator immediately should they manifest any sign or symptom perceived as serious.

## Eliciting & Documenting Adverse Events

### **Definition of an Adverse Event**

Serious adverse events (SAEs) are discussed in Section 11.5 below.

An adverse event (AE) includes any noxious, pathological or unintended change in anatomical, physiological or metabolic functions as indicated by physical signs, symptoms or laboratory test abnormalities occurring in any phase of the clinical study whether associated with the study vaccine and whether or not considered vaccine-related. This includes an exacerbation of pre-existing conditions or events, intercurrent illnesses, or vaccine or drug interaction or the significant worsening of the disease under investigat-ion that is not recorded elsewhere in the CRF. A symptom present just prior to a first vaccination, and not exacerbated after the vaccination is administered, should not be considered an AE. Similarly, anticipated day-to-day fluctuations of pre-existing conditions, including the disease under study, that do not represent a clinically significant exacerbations need not be considered AEs. However, discrete episodes of chronic conditions occurring during a study period (after the first vaccination) should be reported as AEs in order to assess changes in frequency or severity.

AEs should be documented in terms of a medical diagnosis. When this is not possible, an AE will be documented in terms of signs and symptoms observed by the investigator or reported by the subject at each study visit.

Pre-existing conditions, or signs and symptoms (including any which are not recognized at study entry but are recognized during the study period), which are present in a subject prior to the start of the study should be recorded on the Medical History Form within the subject's CRF. Any of the signs or symptoms to be solicited present during physical examination of the subject at each vaccination visit should be recorded on the Pre-Vaccination Assessment page of the subject's source document.

AEs which occur after informed consent is obtained, but prior to a first vaccination, will be documented on the Medical History Form within the subject's CRF.

Any non-elective hospitalization will be considered an SAE, rather than an AE, regardless of the reason for hospitalization and possible causal relationship to vaccine administration (See Section 11.5).

Solicited AEs to be recorded are described in Section 11.1.5 . Any other AEs will be recorded as unsolicited AEs.

### **Surveillance Period for Occurrence of Adverse Events**

All AEs occurring within one month (maximum 30 days) following administrat-ion of each dose of vaccine must be recorded on the Adverse Event Form in the subject's CRF, irrespective of severity or likelihood of vaccination-relatedness.

### **Recording of Adverse Events**

At each visit/assessment, all AEs either observed by the investigator or one of his clinical collaborators or reported by the subject’s parent(s) spontaneously or in response to a direct question will be evaluated by the investigator. AEs not previously documented in the study will be recorded in the Adverse Event Form within the subject's CRF. The nature of each event, date and time (where appropriate) of onset, outcome, intensity and relationship to vaccination will be established. Details of any corrective treatment should be recorded on the appropriate page of the source document. See Section 11.5 for instructions for reporting and recording of SAEs.

As a consistent method of soliciting AEs, the subject’s parent(s) will be asked a non-leading question such as:

"Has the child behaved or reported feeling different in any way since receiving the vaccine or since the previous visit?"

N.B. The investigator should record only those AEs having occurred within the time frames defined above, see Section 8.2.

AEs already documented in the CRF, i.e. at a previous assessment, and designated as ‘ongoing’ should be reviewed at subsequent visits, as necessary. If these have resolved, the documentation in the CRF should be completed. If an AE changes in frequency or intensity during a study period, a new record of the event will be started.

Solicited AEs will be elicited for the first 4 days and on day 7 post-immunization and unsolicited AEs will be recorded during a 30-day follow-up period (day of vaccination and 29 subsequent days). SAE’s will be recorded through the 6^th^ month after the last vaccination (through Study month 8). Thereafter, SAEs are not recorded/reported, although malaria episodes are documented and blood samples are collected for immunological testing.

### **Reporting of Adverse Events**

Reports detailing solicited AEs will be provided by the PI to the DSMB (See Sections 7.9.3 and 7.9.4 for details) after doses 1a and 1b (See also Section 8.1.2: ‘Vaccination Process’ and Appendix B [Section 17.2.5: ‘Specific Schedule of Events’]). These reports will reflect solicited AE data as of Study days 7 and 21, respectively. It is on the basis of its review of these reports that the DSMB renders concurrence or non-concurrence for the dose escalations represented by doses 1b and 1c, respectively. Add-itionally, the DSMB’s concurrence to proceed to dose 1c, would represent concurrence for all subsequent doses (Doses 2a through 3c) as well. Although the DSMB receives 2 more AE/SAE reports (after consecutive doses 2 and 3; see Section 7.9.4), no further directives are required for the subsequent dose escalations.

Reports detailing solicited AEs will be provided by the PI to the LMM 8 days after each of the 9 doses (i.e., provisionally on, Study days 8, 22, 36, 37, 50, 64, 65, 78, and 92) After reviewing each report, the LMM will, if he concurs, recommend proceeding with the next scheduled dose. This concurrence may be given informally (e.g., telephonically) but will be recorded by the PI in the study regulatory file.

### **Solicited Adverse Events**

**Table 12. Solicited Adverse Event (AE) Categories**

| Categories | Adverse Events |
| --- | --- |
| Local (injection site) AEs | Pain at the injection site |
|  | Swelling at the injection site |
| General (systemic) AEs | Fever (i.e., axillary temperature ≥ 37.5°C) |
|  | Drowsiness |
|  | Loss of appetite |
|  | Irritability/fussiness |

N.B.: Axillary temperature will be recorded at the time of the clinic visit. Should additional temperature measurements be recorded at another time of day, the highest temperature will be recorded.

The assessment of severity/intensity will be as described in Section 11.2. For general signs and symptoms reported, the investigator should assign a causality as described in Section 11.3.

For all signs and symptoms reported, the investigator should report the outcome as described in Section 11.4.

**If an SAE (see Section 11.5) occurs at any time during the study period, the PI will inform the USAMRMC-RCQ within 24 hours by e-mail, fax or telephone and with a written report within 3 working days. The WRP/KEMRI Ethics Review Commit-tee, GSK Biologicals, HSRRB, MVI, the DSMB and the Local Medical Monitor will also receive the written report and so will also be notified within 3 working days.**

### **Unsolicited Adverse Events**

Space will be provided in the CRF for the recording of unsolicited symptoms. Unsolicited symptoms are any AEs reported by the subjects or their parents within 30 days of a vaccination that are different from those solicited symptoms recorded during the 7 day post-vaccination follow-up period. Should any general (systemic) signs or symptoms be reported, their relationship with the study vaccine will be assessed by the investigator and transcribed into the CRF, as described in Section 11.3.

## Assessment of Intensity

Intensity of solicited AEs will be assessed and graded by the investigator as presented in Table 13.

| **Table 13. Assessment of Solicited Adverse Event (AE) Intensity** | | |
| --- | --- | --- |
| **AE** | **Grade** | **Intensity Definition** |
| Pain at injection site | 0 | Absent |
|  | 1 | Minor reaction to touch |
|  | 2 | Cries/protests on touch |
|  | 3 | Cries when limb is moved/spontaneously painful |
| Swelling at injection site | 0 | Absent |
|  | 1 | < 5 mm |
|  | 2 | 5-20 mm |
|  | 3 | > 20 mm |
| Fever | 0 | < 37.5°C |
|  | 1 | 37.5-38.0°C |
|  | 2 | 38.1-39.0°C |
|  | 3 | > 39.0°C |
| Irritability/Fussiness | 0 | Behavior as usual |
|  | 1 | Crying more than usual/ no effect on normal activity |
|  | 2 | Crying more than usual/ interferes with normal activity |
|  | 3 | Crying that cannot be comforted/ prevents normal activity |
| Drowsiness | 0 | Behavior as usual |
|  | 1 | Drowsiness easily tolerated |
|  | 2 | Drowsiness that interferes with normal activity |
|  | 3 | Drowsiness that prevents normal activity |
| Loss of appetite | 0 | Normal |
|  | 1 | Eating less than usual/ no effect on normal activity |
|  | 2 | Eating less than usual/ interferes with normal activity |
|  | 3 | Not eating at all |

NB: These intensity grades for solicited AEs are consistent with the intensity grades for the same events presented “Table 5: General Toxicity Table”, except that in Table 5 the grade 3 intensities (for all 6 events) are further divided into grade 3 and 4 intensities.

For each solicited symptom, the subject’s parent(s) will be asked if medical advice was sought (i.e. contact attempted with medical personnel) for this symptom.

For all other AEs, maximum intensity should be assigned to one of the following categories:

0 = No adverse event

1 = An adverse event that is easily tolerated by the subject, causing minimal discomfort and not interfering with everyday activities.

2 = An adverse event that is sufficiently discomforting to interfere with normal everyday activities.

3 = An adverse event that prevents normal, everyday activities. (Such an adverse event would, for example, prevent attendance at school, church or other typical assemblies, and would necessitate the administration of corrective therapy.)

## Assessment of Causality

Every effort will be made by the investigator to explain each AE and determine its causal relationship, if any, to administration of the study vaccine.

The degree of certainty with which an AE can be attributed to administration of the study vaccine (or alternative causes, e.g., natural history of underlying diseases, concurrent therapy, etc.) will be determined by how well the event can be understood in terms of one or more of the following:

- Reactions of a similar nature having previously been observed with this type of vaccine or formulation.
- The event having often been reported in literature for similar types of vaccines.
- The event being temporally associated with vaccination or reproduced on re-vaccination.

All solicited local (injection site) reactions will be considered causally related to vaccination.

Assessment of causality in all other AEs should be documented by the investigator by answering the following query on the “Serious and Unexpected Adverse Event Report”:

In your opinion, did the vaccine(s) possibly contribute to the adverse event?

NO; Either the adverse event is not causally related to administration of the study vaccine or

there are other, more likely causes and administration of the study vaccine is not suspected

to have contributed to the adverse event.

YES; There is a reasonable possibility that the vaccine contributed to the adverse event.

Non-serious and serious AEs will be evaluated as two distinct events given their different medical nature. If an event meets the criteria to be determined “serious” (see Section 11.5.1 for definition of an SAE), it will be investigated by the PI to the extent that a determination of ALL relevant contributing factors can be made.

A partial list of possible contributing factors might include:

- Past medical history
- Concurrent medications
- Protocol required procedures
- Lack of efficacy of the test vaccine
- Erroneous vaccine administration

## Adverse Event Follow-up & Assessment of Outcome

Investigators should follow-up subjects with SAEs until the event has subsided, or until the condition has stabilized, regardless of when this occurred in relation to the study conclusion. Investigators should follow-up subjects with non-serious AEs until study conclusion for a given subject. Thus, all follow-ups for a given cohort end on the same calendar day, but the day follow-ups end is different for each cohort. This will be exactly 8 months after the last subject of a given cohort was enrolled into the study. Re-ports relative to the subsequent course of an AE noted for any subject must be submitted to the Study Monitor.

Outcome should be documented as one of the following:

1 = Recovered without sequelae

2 = Recovered with sequelae

3 = Ongoing at subject study conclusion

3a = Ongoing and worsened

3b = Ongoing, but improved

3c = Ongoing and unchanged

4 = Died

5 = Unknown

## Serious Adverse Events

### **Definition of a Serious Adverse Event**

A *serious adverse event* (SAE) is any untoward medical occurrence that results in death, is *life threatening*, results in persistent or significant *disability or incapacity*, requires in-patient *hospitalization* or prolongation of existing hospitalization or is a congenital anomaly or birth defect in the offspring of a study subject. In addition, important medical events that may jeopardize the subject or may require intervention to prevent one of the other outcomes listed above should be considered serious. (Examples of such interventions include intensive treatment in an emergency room or at home for allergic bronchospasm; blood dyscrasias or convulsions that do not result in hospitalization; and development of drug dependency or drug abuse.)

Although not considered to be (or reported as) SAEs, elective hospitalizations and cancers diagnoses should be reported in the same way as SAEs.

Additional definitions of terms used in the above definition of an SAE are:

Life threatening: An adverse event is life threatening if the subject was at risk of death at the time of the event; it does not refer to an event that hypothetically might have caused death if it were more severe.

Disabling or incapacitating: An adverse event is incapacitating or disabling if the event results in a substantial disruption of the subject's ability to carry out normal life functions. This definition is not intended to include experiences of relatively minor medical significance such as headache, nausea, vomiting, diarrhea, influenza, and accidental trauma (e.g. sprained ankle).

Hospitalization: In general, hospitalization signifies that the subject has been detained (usually involving at least an overnight stay) at the hospital or emergency ward for treatment that would not have been appropriate in the physician’s office or other out-patient setting.

Routine Clinical Procedure: A procedure which is defined in the protocol as one which may take place during the study period and should not interfere with the study vaccine administration or any of the ongoing protocol specific procedures.

**If anything untoward is reported during an elective procedure, that occurrence must be reported as an AE, either ‘serious’ or ‘non-serious’, according to the usual criteria.**

**When in doubt as to whether ‘hospitalization’ occurred or was necessary, the event should be considered an SAE.**

### **Reporting Serious Adverse Events**

In the event that any SAE is suspected of being related to vaccination in any study subject, no further vaccinations will be administered to any study subjects until a written report has been submitted to the sponsor (i.e., Office of the Surgeon General, U.S. Army; POC: Dr. Charles English, USAMMDA, Ft Detrick, MD, USA), GSK Biologicals, MVI, the DSMB, and the FDA, and the investigators have conferred with the LMM.

Any SAEs that are both serious *and* unexpected, whether or not they are judged to be related to the study vaccine or comparator vaccine, will be immediately reported by e-mail or telephone to the USAMRMC Office of Regulatory Compliance and Quality (301-619-2165) (during non-duty hours call 301-619-2165 **and** send information by facsimile to 301-619-7803). A written report will follow the initial report within 3 working days. The report will be addressed to the U.S. Army Medical Research and Materiel Command, ATTN: MCMR-RCQ, 504 Scott Street, Fort Detrick, Maryland 21702-5012. In addition, the following agencies will also receive copies of the written report and so will be within 3 working days: the sponsor, WRP/KEMRI Ethics Review Committee, the LMM, the DSMB, GSK Biologicals and MVI. Every SAE that is not resolved at the time the initial written report is filed will have a follow-up report submitted when information is avail-able. Any submitted report will be identified as "initial," "follow-up," or "medical monitor".

An initial notification should include:

• Study protocol number and the name of the PI

• Subject ID number, initials, age and gender

• Date of onset of the event and date of the most recent administration of the study vaccine

The PI will not wait to collect additional information to fully document the event before making notification of an SAE. The telephone or email report should be followed by a full written report utilizing the “Serious Adverse Events Form” within the CRF, detailing relevant aspects of the SAEs in question.

Instances of death, cancer or congenital abnormality in offspring if brought to the attention of the investigator AT ANY TIME after cessation of study test article AND suspected by the investigator to be related to study medication, should be reported to the Study Monitors.

All information should be sent PROMPTLY **by the PI to the sponsor (i.e., Office of the Surgeon General, U.S. Army; POC: Dr. Charles English, USAMMDA, Ft Detrick, MD, USA), the WRAIR Human Use Research Committee, WRP/KEMRI Ethics Review Committee, MVI, GSK Biologicals, the DSMB and the LMM.** Points of contact for these institutions and committees are given below.

**Human Subjects Research Review Board (HSRRB)**

USAMRMC Deputy for Regulatory Compliance and Quality

Attn: LTC Laura Brosch, Acting Chair, HSRRB

Human Subjects Protection Division

U.S. Army Medical Research and Materiel Command

Fort Detrick, MD, USA

Tel: DSN 343-7803 or 301-619-2165/6

Fax: DSN 343-7803 or 301-619-7803

**WRAIR Human Use Research Committee (HURC):**

Dr. Sara Rothman

Office of Research Management

WRAIR, Silver Spring, MD, USA

Tel 301-319-9961

Fax 301-319-9940

**WRP/KEMRI Ethics Review Committee (ERC):**

Dr. Monique K. Wasunna

Director, Centre for Clinical Research

Kenya Medical Institute of Research

Nairobi, Kenya

Tel 254-2-722541

**Malaria Vaccine Initiative (MVI):**

Ms. Jessica Milman

Program Officer

Malaria Vaccine Initiative at PATH

Tel 301 770-5377

Fax 301 770-5322

**Local Medical Monitor** **(LMM)**

Dr. Ambrose Misore

Nyanza Provincial Headquarters

P.O.Box 721

Kisumu, Kenya

Tel 254-57-40091/41550

Fax 254-57-21870

*Alternate LMM:*

Dr. Juliana Otieno

Chief, Dept of Pediatrics

New Nyanza Provincial General Hospital

P.O.Box 849

Kisumu, Kenya

Cell 254-733-715917

Fax 254-57-41330

GlaxoSmithKline Biologicals (GSK Biologicals):

Alfred Tiono, MD

WHO/GSK Biologicals Trainee

Rue de L’Institute, 89

B-1330 Rixensart Belgium

Tel: +32-2-656-6535

Fax: +32-2-656-6160

*Alternate:*

**Dr Marc Ceuppens**

Manager Clinical Safety Vaccines

Tel: +32 2 656 8798

Fax: +32 2 656 8009

**Data & Safety Monitoring Board (DSMB):**

*DSMB Chairman:*

Professor Fred Binka,

School of Public Health

University of Ghana

P.O. Box LG 13

Legon / Ghana

Tel: +233 21-500 388

Email: [fbinka@africaonline.com.gh](mailto:fbinka@africaonline.com.gh)

*Other DSMB members:*

Dr. Marcel Tanner

Professor & Director, Swiss Tropical Institute

Socinstrasse 57 P.O. Box 4002

Basel / Switzerland

Tel: +41 (61) 2848283

Email: [marcel.tanner@unibas.ch](mailto:marcel.tanner@unibas.ch)

Dr. William Blackwelder

8613 Hempstead Avenue

Bethesda, MD 20817

Tel: (301) 564-6137

Email: [wcb@boo.net](mailto:wcb@boo.net)

Dr. Clara Menendez

Unidad de Epidemiologia Hospital Clinica

(Universidad de Barcelona)

c/ Villarroel 170 E-08036

Barcelona Spain

Tel: 34 932 275 706

Email: [cmenende@medicina.ub.es](mailto:cmenende@medicina.ub.es)

Dr. Norbert Peshu

Centre for Geographic Medicine Research-Coast

Kenya Medical Research Institute

Tel: 254 125 22063

Fax: 254 125 22390

Email: npeshu@kilifi.mimcom.net

## Treatment of Adverse Events

Treatment of any AE is at the sole discretion of the investigator or the LMM and should be in accord with accepted, local standards of good medical practice. The applied measures should be recorded in the source documents of the vaccinee. The recording of AEs is an important aspect of study documentation. It is the responsibility of the investigator to document all AEs according to the detailed guidelines set out herein. The subjects’ parents will be instructed to contact the PI immediately should a subject manifest any sign or symptom perceived as serious.

# SUBJECT COMPLETION & DROP-OUT

## Definition of a Drop-out

From the perspective of data analysis, a 'drop-out' is any subject who is not brought back for the concluding visit foreseen in the protocol. A subject who is brought in for the concluding visit foreseen in the protocol is considered to have completed the study and is therefore not a drop-out.

## Procedures for Handling Drop-outs

Investigators should make an attempt to contact parents of those subjects who have not been brought for scheduled visits or follow-up. Information gathered should be described on the Study Completion page of the CRF and in source documents (progress notes).

## Reasons for Drop-outs

The Study Completion page of the CRF will specify which of the following possible reasons were responsible for drop-out of the subject from the study:

• Serious adverse event (SAE)

• Non-serious adverse event (AE)

• Protocol violation (Specify)

• Withdrawal of parental consent, not due to an AE or SAE

• Migration of family or subject from the study area

• Loss to follow-up

• Other (Specify)

# DATA MANAGEMENT & ANALYSIS

Data entry will be performed on site at the WRP Kombewa Clinic. Duplicate CRF pages will be removed by the study coordinators periodically (after review and/or approval of the study monitors) and submitted to the data entry center. Data analysis and reporting will be done by Statistics Collaborative, Inc., Washington, DC, USA.

## Primary Endpoints

Occurrence of solicited symptoms during a 7 day follow-up period after each vaccination (5 visits: day of vaccination and days 1, 2, 3, and 7)

Occurrence of unsolicited symptoms during a 30-day follow-up period after vaccination (day of vaccination and 29 subsequent days)

Occurrence of any SAEs during an 8 month follow-up period following the first dose of study vaccine (i.e., 6 months following the last dose)

## Secondary Endpoints

Anti-MSP-1 antibody titers at timepoints at which blood samples are taken for serology.

## Study Cohorts & Data Sets to be Evaluated

### **Total Cohort**

The “total cohort” will include all subjects enrolled in the study.

### **Safety Cohort**

The “safety cohort” will consist of all subjects who have received at least one dose of study vaccine or comparator vaccine and for whom any data on safety are available for analysis. A subset of the safety cohort will include those who received the test article vaccine.

The presentations of safety data will explore separately the adverse experiences among subjects who received all 3 vaccinations and among those who received fewer; among those who received each of the 3 vaccine doses; and among those with clinically important violations of study protocol.

### **Immunogenicity Cohort**

The “immunogenicity cohort” will include all subjects for whom assay results are available for antibodies against the specified study vaccine antigen component or the comparator. There are no standard “cut off” titres for seropositivity determinations in general use for this assay.

## Sample Size

This phase I trial consists of what are essentially 9 small treatment groups (10 subjects each) and 9 small control groups (5 subjects each; See Table 2, Section 7.2). It does not have adequate power to detect small differences between groups. Although comparative statistics for the safety variables will be calculated, the study will have low power to detect anything other than large differences in the incidence of local and general side effects between the vaccination groups. It will be more useful and significant to compare symptom rates for the test article recipients of entire cohorts (N=30) versus that cohort’s comparators (N=15), rather than, for example, Cohort A’s 12-23 month olds receiving FMP-1 (N=10) versus Cohort A’s 12-23 month old Imovax® recipients (N=5).

Sample size was chosen after weighing the need to detect any possible untoward reactions against the need to limit the number of subjects involved for safety purposes. A total sample size of about one hundred (135 in this case) is typical for the initial assessments of the safety, tolerability, and immunogenicity of an investigational vaccine. Incorporation of the Imovax® Rabies vaccine comparison group will enable broad initial estimates of the incidence of local and general side effects and of immune responses among vaccine recipients.

**13.5 Final analyses**

### Analysis of Demographics

Demographic characteristics (age, gender, etc) of each study cohort will be tabulated. The mean age (with range and standard deviation) by gender of the enrolled subjects, by dose group, and in aggregate, will be reported.

### Analysis of Immunogenicity

For each dose group and age group, and at each time point, anti-MSP-1 antibody levels will be presented as geometric means of OD units with 95% confidence interval tabulated. The time course of antibody levels will be described through longitudinal models.

### Analysis of Safety

The overall percentage of subjects with at least one local AE (solicited and unsolicited) and the percentage with at least one general AE (solicited and unsolicited) during the four-day follow-up period after vaccination will be tabulated. The incidence, intensity, and relationship of individual solicited symptoms over a one week period of follow‑up (day of vaccination, plus days 1, 2, 3, and 7) will be calculated per age group and dose.

The number of subjects with at least one report of unsolicited AE, classified by the “World Health Organization Preferred Terms”, reported up to 30 days after vaccination will be tabulated by age group and dose. The intensity and relationship to vaccination of the unsolicited symptoms reported will also be assessed.

SAEs are expected to be rare, but where observed will be described. Comparisons of incidence of all symptoms, local symptoms and general symptoms will utilize a two-sided Fisher’s Exact Test. Analysis of safety during the 8-month follow-up period (6 months after dose 3) will consist of comparison of incidence of SAEs as well as hemoglobin levels.

### 13.5.4 Clinical Laboratory Parameters

Hematological (CBC) and biochemical (ALT, creatinine) laboratory parameters will be measured at specific time points, *viz*., days 0, 14, 30, 44, 60, 74, 90, and starting on day 180 every 3 months for Hbg. Clinically relevant abnormal values will be tabulated and longitudinal analysis could be performed if deemed useful.

## Preliminary Analysis

Safety, reactogenicity, and immunogenicity data after 3 vaccine doses will be compiled by Statistics Collaborative, Inc., Washington, D.C. from the CRF data trans-cribed on site in Kenya. This will initially be done after the end of the “Follow-up Period for Unsolicited AEs” (Day 120), when all available data will be complied into an Interim Safety Data Report (statistical analysis) for submission to the DSMB in early October 2003. The Final Safety Data Report to the DSMB will be compiled after the end of the “Post-vaccination Safety Surveillance Period for SAEs” (Day 240) for submission in November 2004. For both reports, safety will be analyzed for dose group overall and by age and dose group without revealing the assignment of individual subjects. The PI and Kenya field team members will remain blinded until one month (30 days) after the 30 day post-vaccination follow-up visit for Cohort C’s third vaccine dose (according to the current provisional daily schedule, that visit comes on Study Day 114 and so unblinding would be on Study Day 144). This unblinding will be a “by group”, but not a “by individ-ual” unblinding. (The purpose of this partial unblinding is to allow assessments of the "by group" data which are requisite for the planning of any subsequent phase I or phase II pediatric trials of MSP-1 -- particularly where dosages and sample sizes are concerned.) The full study unblinding for individual results will be after the end of the “Post-vaccination Safety Surveillance Period for SAEs” (Study Day 240).

## 13.7 Administrative Matters

To comply with Good Clinical Practice (GCP), important administrative obligations relating to investigator responsibilities, monitoring, archiving data, audits, confidentiality and publications must be fulfilled. These are outlined in Appendices C and F. Any missing, unused or spurious data, or previous reports on any deviations from the original statistical plan, will be cited and accounted for in the appropriate analysis (preliminary, interim or final; open or closed; blinded, partially unblended, or unblinded) as they become apparent to study investigators or statisticians.

# ETHICAL CONSIDERATIONS

## Ethics & Regulatory Considerations

The study will be performed under FDA IND 9202. The study will be conducted according to Good Clinical Practice, US 21 CFR Part 50—Protection of Human Subjects, and Part 56—Institutional Review Boards, U.S. Army Regulation AR 40-38, and AR 70-25 and local rules and regulations of the host country.

Primary monitoring responsibilities will be undertaken by USAMMDA, with GSK Biologicals as a co-monitor. However, if USAMMDA is unable to provide a monitor for the site, MVI will provide a back-up monitor through its existing contract with PPD. The term “study monitor” applies to designated representatives of any of these institutions. Scientific review committees of the WRAIR and KEMRI will have reviewed the study. In addition to the review by the Human Subjects Research Review Board (HSRRB) of the Office of the Surgeon General, US Army, the study will be reviewed and approved by the Kenya Medical Research Institute (KEMRI) Ethics Review Committee (ERC) according to the local laws and customs of Kenya. Documentation of this approval will be submitted to the HSRRB. The study will also be reviewed and approved by the Program for Appropriate Technology in Health (PATH) Human Subjects Protection Committee (HSPC). All other required approvals (initial, continuing review, amendments) must be secured from the primary IRB(s) prior to submission to the HSRRB.

### 14.1.1 Institutional Review Board/Ethics Review Committee (IRB/ERC)

The IRB/ERC must be constituted according to the local laws and customs of each participating country. It is recommended that it should include:

At least 5 members.

At least one member whose primary area of interest is non-scientific.

At least one member who is independent of the sponsoring institution and study site.

Only those IRB/ERC members who are independent of the investigator and the study sponsor

should vote and provide opinions on study-related matters.

A list of IRB/ERC members will be kept in the investigator’s study file.

All amendments will be submitted to the HSRRB through Office of Research Management, and to the KEMRI Ethical Review Committee, and to PATH HSPC. No amendments will go into effect without written approval from HSRRB, KEMRI ERC, and PATH HSPC except when the amendments are purely of an administrative nature or when the changes are necessary to eliminate immediate hazards to study subjects. Administrative changes to the protocol are defined as corrections or clarifications that have no effect on the way the study is conducted. In such cases, the amendment will be submitted to the IRB/ERC and written verification that the modification was submitted will be obtained. Administrative changes will be agreed upon by the sponsor, and the PI and will be documented, and the local IRB and the HSRRB will be notified in writing of any administrative changes.

At a minimum, the IRBs/ERCs must be informed in a timely manner by the investigator of the following:

- All subsequent revisions of study documents originally submitted for review.
- All subsequent protocol modifications, as opposed to amendments, for information.
- Any serious and unexpected adverse events occurring during the study.
- Any deviations from the approved protocol procedures.
- Where required, new information that may affect adversely the safety of the subjects or the conduct of the study.
- Annual update or request for re-approval, where required, when the study has been completed.

This study is to be conducted in accordance with 21 CFR 56, which requires that changes in approved research may not be initiated without IRB review and approval except where necessary to remove apparent immediate hazards to the human research subject.

## The Local Medical Monitor (Local Safety Monitor)

The Local Medical Monitor (LMM) will be an experienced, host nation physician based near the study site. He or she must have the experience required to monitor human subjects during the conduct of a clinical trial and must be prepared to provide care to research subjects if any such need arises during the conduct of the study. In addition to the LMM, an alternate LMM, possessing the same qualifications, will be designated before study commencement. This alternate LMM will assume all the LMM’s responsibilities in the event that the latter cannot meet his or her obligations for whatever reason.

The overall role of the LMM will be to vouchsafe responsible actions with regard to the ethics and clinical safety of a study, especially in regard to the proper assessment of AEs. At the same time he or she should support the clinical investigators and act as a facilitator between them and the DSMB.

The PI will report all SAEs to the LMM, who will review them and provide an unbiased written report of events within 10 calendar days of the initial report. At a minimum, this report will comment on the outcomes of the SAE and relationship to the test article and indicate concurrence or nonconcurrence with the details of the report provided by the PI. Reports detailing solicited AEs will be provided by the PI to the LMM 8 days after each of the 9 doses (i.e., Study Days 6, 20, 34, 49, 62, 69, 83, 90; NB: There are 8 report days for reports on 9 doses because reports on doses 1c and 2a will both be provided on Study Day 34.) Courtesy copies of these reports will also be sent (by email or fax) to GSK Biologicals, per their request. After reviewing each report, the LMM will, if he concurs, recommend proceeding with the next scheduled dose. This concurrence may be given informally (e.g., telephonically) but will be recorded by the PI in the study regulatory file.

The LMM’s involvement will be particularly important when decisions have to be made quickly. Code break envelopes will be in the safekeeping of the LMM, who may unblind individual study subjects if that is deemed necessary for medical or ethical reasons. In exceptional circumstances (e.g., a death possibly related to vaccination) the LMM would have the authority to suspend the whole or any specific aspect of the trial. The LMM may then call for trial resumption after discussion with, and approval by, the DSMB.

The LMM’s role will include:

- Providing advice to study investigators on whether a set of clinical circumstances in a study warrants formal notification of the DSMB, the study sponsor or others.
- Providing clinical advice on any illness in study subjects especially in circumstances in which treatment might influence the course of the trial.
- Review all SAEs as outlined above.
- Review 9 reports of all solicited AEs (after Doses 1a, 1b, 1c, 2a, 2b, 2c, 3a, 3b and 3c) and render concurrence/non-concurrence on subsequent doses as outlined above.
- Make at least two obligatory visits to the WRP Kombewa Clinic to directly observe selected ongoing study operations.

The LMM will work closely with the PI throughout the course of the trial and relay any relevant safety information to the PI. It is the joint responsibility of the LMM and PI to convey this information to the sponsor, the DSMB and MVI.

## The Data & Safety Monitoring Board (DSMB)

### Composition of the Board

For purposes of this study, the Data and Safety Monitoring Board (DSMB) is an independent committee consisting of up to 5 experts in malaria, infectious diseases, biostatistics and other appropriate disciplines who are appointed to oversee the ethics and safety aspects of study conduct.

### Role of the Board

The DSMB may convene at any time during the study to review relevant safety data and to review and approve the Reporting and Analysis Plan (RAP). Unscheduled meetings may be required. Meetings must be documented and minutes made available for the study files on site and to the sponsor. The DSMB may, if deemed necessary, convene a meeting with, or request further information from, the PI, the LMM, and designated project representatives of the WRAIR, GSK Biologicals, and MVI /PATH at any stage of the study.

The main role of the DSMB is to review and analyze clinical safety data collected during the trial and to assess SAEs.

The PI must provide the DSMB with:

- Prompt reports of any SAEs occurring during the study.
- Routine reports of solicited AEs after doses 1a and 1b, as well as after dose 3c. (The first 2 reports will result in notification of concurrence or non-concurrence by the DSMB for proceeding with the subsequent dose escalations (i.e., doses 1b and 1c, respectively; See Section 11.1.4: ‘Reporting of Adverse Events’, Section 8.1.2: ‘Vaccination Process’ and Appendix B [Section 17.2.5: ‘Specific Schedule of Events’]).
- Any protocol amendments, informed consent changes or revisions of other documents originally submitted for review.
- Any protocol modifications (for information).
- Any new information that may affect adversely the safety of the subjects or the conduct of the study.

The DSMB will be empowered to put the study on hold pending review of potential safety issues (see Section 7.9.2). All SAEs, including death, will be reported by the PI to the DSMB, the Manager of Clinical Safety Vaccines at GSK Biologicals, the HSRRB, and MVI. All SAEs will be provided on both an “as-randomized” and an “as received” basis to the board, which will review for data trends in relation to safety issues and will have the right to seek additional clinical data about all cases. The DSMB will seek the input of the PI as needed. The DSMB may perform any of its own statistical calculations deemed necessary to support recommendations to the sponsor. All documentation provided to Board members for information and review must be treated in a confidential manner.

MVI will propose new members for the committee in the event that members must be replaced.

## Risks & Potential Benefits to Subjects

### Vaccination

Risks associated with vaccination include local inflammatory reactions to the injected product, such as injection site pain and swelling and some limitation of leg movement. Systemic effects may include flu-like syndrome, fever, nausea/GI symptoms, headache, malaise, myalgia, and arthralgia. To date all the symptoms associated with the FMP-1/AS02A vaccine have been transient, mainly mild to moderate in intensity and have resolved without sequelae. While exceedingly rare, allergic reactions, to include life-threatening anaphylaxis, are associated with many vaccine preparations and must therefore be considered as a potential risk in this study.

### Medical Treatment of Subjects

Free medical treatment will be provided to all enrolled subjects during the active immunization phase and the follow-up period. The pharmacy at the WRP Kombewa Clinic will be able to provide subjects with common over-the-counter analgesics such as paracetamol, as well as antibiotics for the treatment of minor infections free of charge. If further evaluation or treatment is necessary, the subject will be referred to the local health facility (Kombewa Sub-District Hospital; KSDH) which is located a few steps away from the study clinic. After duty hours, subjects will be able to receive medical attention at the study clinic or at this hospital. If in the judgment of the PI, or the clinical officer on duty, a subject requires hospitalization, referral to the KSDH or to the NNPGH in Kisumu will be arranged.

Medical care for ailments not related to vaccination will not extend beyond the study period. Medical care for ailments related to vaccination will extend at least until the condition has resolved.

### Rabies Vaccination

During the conduct of the study, subjects randomized to receive the Imovax® Rabies vaccine will benefit from this due to the high prevalence of rabies in Kenya. At the end of the study all subjects’ parents will be informed of which vaccine the subject received. Subjects randomized to the FMP-1 vaccine will be offered rabies vaccine at that time. This later dosing will be done at the recommended schedule of 0, 7, and 21 (or 28) days.

## Precautions to Minimize Risk

### Vaccination

As outlined above, the subjects will be monitored closely during their participation in this study. The study vaccine has been prepared according to Good Manufacturing Procedures (GMP). The vaccines will be administered in the WRP Kombewa Clinic under the supervision of an investigator with adequate drugs and equipment available for the treatment of anaphylaxis. All vaccine doses will be given by slow injection to minimize injection site reactions such as pain.

### Malaria Treatment During the Study

Medications available for the treatment of clinical malaria (see Section 8.3.2) will include oral Coartem, Malarone, and sulfadoxine/pyrimethamine. Oral and parenteral quinine will also be available.

## Procedures for Maintaining Confidentiality

Subjects will be assigned a unique identifier number, the Subject Identification (SID) number. All results will be linked to this number. Study records will only be available to staff members and will be kept in locked cabinets at the study site. Following the conclusion of the study, records will be maintained on site for a minimum of two years. These records may be reviewed by representatives of KEMRI, the USAMRMC, the FDA, and the study sponsor as part of their responsibility to oversee this research.

# REFERENCES

1. Nchinda TC. Malaria: A reemerging disease. Emerging Infectious Dis. 1998; 4(3):398-403.
2. Sturchler D. How much malaria is there worldwide? Parasitol Today 1989;5:39.
3. Blackman MJ, Heidrich HG, Donachie S, et al. A single fragment of a malaria merozoite surface protein remains on the parasite during red cell invasion and is the target of invasion-inhibiting antibodies. J Exp Med 1990 July; 172:379-382.
4. Chang SP, Gibsoon HL, Lee-Ng CT, et al. A carboxyl-terminal fragment of *Plasmodium falciparum* gp195 expressed by a recombinant baculovirus induces antibodies that completely inhibit parasite growth. J Immunol July 1992; 149(2):548-555.
5. Egan AF, Morris J, Barnish G, et al. Clinical immunity to *Plasmodium falciparum* malaria is associated with serum antibodies to the 19-kDa c-terminal fragment of the merozoite surface antigen, PfMSP-1. J Infect Dis March 1996; 173:765-769.
6. Chang SP, Case SE, Gosnell WL, et al. A recombinant baculovirus 42-kilodalton c-terminal fragment of *Plasmodium falciparum* merozoite surface protein 1 protects *Aotus* monkeys against malaria. Infect Immun 1996 Jan; 64(1):253-261.
7. Ling IT, Ogun SA, and Holder AA. Immunization against malaria with a recombinant protein. Parasite Immunol 1994; 16:63-67.
8. Daly TM and Long CA. A recombinant 15-kilodalton carboxy-terminal fragment of *Plasmodium yoelii* 17XL merozoite surface protein 1 induces a protective immune response in mice. Infect Immun 1993 June; 61(6):2462-2467.
9. Daly T and Long CA. Humoral response to a carboxy-terminal region of the merozoite surface protein-1 plays a predominant role in controlling blood-stage infection in rodent malaria. J Immunol 1995; 155;236-243.
10. Burghaus PA, Wellde BT, Hall T, et al. Immunization of *Aotus nancymai* with recombinant C terminus of *Plasmodium falciparum* merozoite surface protein 1 in liposomes and alum adjuvant does not induce protection against a challenge infection. Infect Immun 1996 Sept; 64(9):3614-3619.
11. Crisanti A, Müller HM, Hilbich C, et al. Epitopes recognized by human T cells map within the conserved part of the GP190 of *P. falciparum*. Science June; 19??; 240: 1324-1326.
12. Rzepczyk CM, Ramasamy R, Mutch DA, et al. Analysis of human T cell response to two *Plasmodium falciparum* merozoite surface antigens. Eur J Immunol 1989; 19:1797-1802.

1. Wu JY, Gardner BH, Murphy CI, et al. Saponin adjuvant enhancement of antigen-specific immune responses to an experimental HIV-1 vaccine. Journal of Immunology 1992;148:1519-25.
2. Soltysik S, Bedore DA, Kensil CR. Adjuvant activity of QS21 isomers. Annals of the New York Academy of Sciences 1993; 690:392-395.
3. Ribi E, Cantrell J, Feldner T, et al. Biological activities of monophosphoryl lipid A, in Microbiology 1986, Levine L, Bonventre PF, Morello JA, et al., Washington D.C., 1986.
4. Myers KR, Truchot AT, Ward J, et al. "A critical determination of Lipid A endotoxin activity", in Cellular and Molecular Aspects of Endotoxin Reactions: Proceedings of the 1st Congress of the International Endotoxin Society. 1990, Nowotny A (ed), Elsevier, Amsterdam, pp 145-156, 1990.
5. Loppnow H, Durrbaum I, Brade H, et al. "Lipid A, the immunostimulatory principle of lipopolysaccharides ?", in Advances in Experimental Medicine and Biology, Friedman H, Klein TW, Nakano M, et al. (eds) 256, pp 561-566, 1990.
6. Johnson AG, and Tomai MA, "A study of the cellular and molecular mediators of the adjuvant action of a nontoxic monophosphoryl lipid A", in Advances in Experimental Medicine and Biology, Friedman H, Klein TW, Nakano M, et al. (eds) 256, pp 567-579, 1990.
7. Stoute JA, Slaoui M, Heppner DG, et al. A preliminary evaluation of a recombinant circumsporozoite protein vaccine against *Plasmodium falciparum* malaria. N Engl J Med 1997; 336: 86-91.
8. Open phase I study to evaluate the safety, reactogenicity and immunogenicity of SmithKline Beecham Biologicals' vaccine against malaria. (Malaria-002). Data on file at SmithKline Beecham Biologicals.
9. Kester KE, McKinney DA, et al. Efficacy of a recombinant circumsporozoite protein vaccine regimens against experimental *Plasmodium falciparum* malaria. J Infect Dis 183:640-7, 2001.
10. Open phase I study to evaluate the safety, reactogenicity and immunogenicity of Smithkline Beecham Biologicals’ candidate vaccine against malaria in semi-immune adult males in the Gambia. Protocol 257049/004 (Malaria-004) - Interim safety report March 23, 1998. Data on file at SmithKline Beecham Biologicals.
11. Stoute JA. Unpublished results.
12. World Survey of Rabies, No. 34. 1998.
13. Dreesen DW, et al. Two-year comparative trial on the immunogenicity and adverse effects of purified chick embryo cell rabies vaccine for pre-exposure immunization. Vaccine. 1989; 7: 397-400.
14. Dreesen DW. Investigation of antibody response to purified chick embryo cell tissue culture vaccine (PCECV) or human diploid cell culture vaccine (HDCV) in healthy participants. Study synopsis 7USA401RA, September 1996 - December 1996 (unpublished).
15. Nicholson KG, et al. Pre-exposure studies with purified chick embryo cell culture rabies vaccine and human diploid cell vaccine: serological and clinical responses in man. Vaccine. 1987; 5: 208-210.
16. Vodopija I, et al. An evaluation of second generation tissue culture rabies vaccines for use in man: a four-vaccine comparative immunogenicity study using a pre-exposure vaccination schedule and an abbreviated 2-1-1 post-exposure schedule. Vaccine. 1986; 4: 245-248.
17. Wasi C, et al. Purified chick embryo cell rabies vaccine (letter). Lancet. 1986; 1: 40.
18. Cox JH, et al. Prophylactic immunization of humans against rabies by intradermal inoculation of human cell culture vaccine. J Clin Microbiol 1976;3:96-101.
19. Kuwert EK, et al. Some experiences with human diploid cell strain (HDCS) rabies vaccine in pre and post-exposure vaccinated humans. Dev Biol Stand 1978;40:79-88.
20. Ajjan N, et al. Resultats de la vaccination antirabique par le vaccin inactive concentre souche rabies PM/W138-1503-3M cultives sur cellules diploides humaines. Dev Biol Stand 1978; 40:89-100.
21. Costy-Berger F. Vaccination antirabique preventive par du vaccin prepare sur cellules diploides humaines. Dev Biol Stand 1978; 40:101-4.
22. CDC. Recommendations of the Immunizations Practices Advisory Committee (ACIP). Rabies Prevention United States. MMWR 1984, 33:393-402, 407-8.
23. Boe N., et al. Guillain-Barre after vaccination with human diploid cell rabies vaccine. Scand J Infect Dis 1980; 12:231-232.
24. CDC. Adverse reactions to human diploid cell rabies vaccine. MMWR 1980; 29:609-610.
25. Bernard KW, et al. Neuroparalytic illness and human diploid cell rabies vaccine. JAMA 1982: 248:3136-3138.
26. CDC. Systemic allergic reactions following immunization with human diploid cell rabies vaccine. MMWR 1984; 33:185-187.
27. Sharif SK, NA Kimathi and JD Quick, eds., *Clinical Guidelines for Diagnosis and Treatment of Common Hospital Conditions in Kenya*, Nairobi: Ministry of Health, Government of Kenya, 1^st^ Ed., Nov 1994; pp 103-7.
28. Ogutu BR, Smoak BL, Nduati RW, Mbori-Ngacha DA, Mwathe F, Shanks GD. The efficacy of pyrimethamine-sulfadoxine (*Fansidar*) in the treatment of uncomplicated *Plasmodium falciparum* malaria in Kenyan children. Trans R Soc Trop Med Hyg 94:83-84, 2000.
29. Adjulk M, Agnamey P, Bablker A, Bormann A, Brasseur P, Cisse M, et al. Amodiaquine-artesunate vs. Amodiaquine for uncomplicated *Plasmodium falciparum* malaria in African children: a randomised multi-center trial. Lancet 359:1365-1372, 2002.
30. von Seidlein L, Bojang K, Jones P *et al*. A randomized controlled trial of artemether/benflumetol, a new antimalarial and pyrimethamine/sulfadoxine in the treatment of uncomplicated falciparum malaria in African children. Am.J.Trop.Med.Hyg. 1998; **58**:638-44.
31. *Division of AIDS Toxicity Table for Grading Severity of Pediatric Adverse Experiences*. Bethesda, Md: Division of AIDS, National Institutes of Health; 1994.

# STUDY BUDGET

## KEMRI Budget

**[Material has been redacted here]**

## Budget Justification

The above budget was prepared using expense records from previous studies in western Kenya which have utilized similar number of subjects.

# APPENDICES

## 17.1 Appendix A: Study Personnel

### **17.1.1 Roles of Study Personnel**

| **Investigator** | **Title** | **Responsibility** |
| --- | --- | --- |
| Mark Withers, M.D., M.P.H. | Principal Investigator | Responsible for overall execution, protocol preparation, clinic procedures & follow-ups, data analysis, & manuscript preparation |
| José A. Stoute, M.D. | Associate Investigator | Protocol preparation, data analysis, immunizations |
| Bernhards Ogutu, MBChB, PhD | Associate Investigator | Protocol preparation, clinical evaluations, data analysis. Provides overall supervision to junior physicians & clinical officers. |
| John N. Waitumbi, Ph.D. | Associate Investigator | Oversees all immunological assays. |
| Allan G. Otieno, MBChB | Associate Investigator | Clinical evaluations |
| Joram Siangla, M.Sc. | Associate Investigator | Measurement of anti-MSP-1 antibody |
| Joseph Koros, HND | Associate Investigator | Measurement of anti-MSP-1 antibody |
| Willis Okoth, B.Sc. | Associate Investigator | Measurement of anti-MSP-1 antibody |
| D. Gray Heppner, M.D. | Associate Investigator | Protocol preparation, data analysis, immunization |
| Kent E. Kester, M.D. | Associate Investigator | Protocol preparation, clinical evaluations,  Humoral assays |
| James Cummings, M.D. | Associate Investigator | Clinical evaluations |
| Jeff Lyon, Ph.D. | Associate Investigator | Vaccine production, humoral assays |
| Evelina Angov, Ph.D. | Associate Investigator | Vaccine production |
| Alan Magill, M.D. | Associate Investigator | Clinical evaluations |
| Christian Ockenhouse, M.D. | Associate Investigator | Clinical Evaluations |
| Carolyn Holland, MPH | Associate Investigator | Logistics Coordinator for WRAIR |
| Melanie Onyango, BSc | Research Coordinator | Insures subject visit schedules according to protocol. & coordinates Luo language prep-aration of informed consents (written & oral). |
| Denise McKinney, RN, CCRC, CCRA | Clinical Coordinator | Vaccine preparation, randomization, assures compliance with all regulations, including study explanation & obtaining informed consent from parents |
| Kathryn Tucker, M.S. | Statistician | Statistical analysis, preparation of database, and preparation of reports. |
| Janet Wittes, Ph.D. | Statistician |

### **17.1.2 Curricula Vitae of Study Personnel [All but the PI’s have been redacted]**

***LTC Mark R. Withers, M.D., M.P.H.***

DATE OF BIRTH: 24 October 1958

EDUCATION:

Alderson-Broaddus College, Philippi, WV; BA (History) & BS (Biology), 1981

West Virginia University, Morgantown, WV; MD, 1986

Harvard School of Public Health, Boston, MA; MPH, 1997

TRAINING & EXPERIENCE:

Internal Medicine Intern, West Virginia University Hospitals, Morgantown, WV, 1986-87

Deputy Surgeon, 1st Special Operations Command, Ft Bragg, NC, 1988-89

Battalion Surgeon, 1st Bn, 3rd Special Forces Group (Airborne), Ft Bragg, NC, 1989-92

Combat Deployment: Operation Desert Storm, 1991

Internal Medicine Resident, University of Wisconsin Hospitals & Clinics, Madison, WI, 1992-94

Staff Internist, Martin Army Community Hospital, Ft Benning, GA, 1995-96

Director, Post TB Clinic; Ward Attending & Consultant; Staff, Internal Medicine Clinic

Aerospace Medicine Resident, USAF School of Aerospace Medicine, Brooks AFB, TX, 1998

Medical Research Fellow & Staff Scientist, Dept of Virus Diseases, Walter Reed Army Institute of

Research, Washington, DC, 1998-2000

Command Surgeon, ARCENT-Kuwait, Camp Doha, Kuwait, 2000

Staff Scientist, DVD, Walter Reed Army Institute of Research, Silver Spring, MD, 2000-2002

Director, Kombewa Clinic, “The Walter Reed Project”, Kisumu, Kenya, 2002-Present

CERTIFICATIONS & LICENSURES:

Diplomate, National Board of Medical Examiners, 1987

Diplomate, American Board of Internal Medicine, 1995

Diplomate, American Board of Preventive Medicine, 1999

State Medical Licenses: WV (Inactive), NC (Inactive), WI (Active)

PUBLICATIONS:

Withers, MR, SK Shrestha, CW Preston, et al., "Hepatitis E Virus Antibody Levels in an Urban Adult

Population in Nepal", *Am J Trop Med Hyg*, (Submitted).

Seriwatana, J, MR Withers, LN Binn, et al., "Development and Evaluation of a Quantitative Enzyme

Immunoassay for Swine Hepatitis E Virus Antibody and Report of an Archival Serosurvey", *J Virol*

*Methods* (Submitted).

Withers, MR, MT Correa, M Morrow, et al., "Hepatitis E Virus Antibody Levels in North Carolina Swine

Workers, Non-swine Workers, Swine and Murids, *Am J Trop Med Hyg*, 66(4), 2002, pp. 384-388 .

Seriwatana, J, MR Withers, LN Binn, et al., "Development and Evaluation of a Quantitative Enzyme

Immunoassay to Detect Hepatitis E Virus Infections in Swine [Abstract 314], *Program and Abstracts of*

*the 50th Annual Meeting of the American Society of Tropical Medicine and Hygiene*, 65(3), Nov 2001.

Withers, MR, GW Christopher and S Hatfill, "Infectious Disease Considerations" [Book chapter], In:

"Aeromedical Evacuation" 2002 (In press).

Withers, MR, MT Correa, M Morrow, et al., "Hepatitis E Virus Antibody Levels in North Carolina Swine

Workers, Swine and Murids [Abstract 237], *Program and Abstracts of the 49th Annual Meeting of the*

*American Society of Tropical Medicine and Hygiene*, 62(3), March 2000.

Withers, MR and GW Christopher, "Aeromedical Evacuation of Biological Warfare Casualties: A Treatise

on Infectious Diseases on Aircraft", *Mil Med* 165, Suppl. 3:001, 2000.

Withers MR, "You're the Flight Surgeon: Absent Right Pulmonary Artery", *Aviat Space Environ Med*

1999 (June); 70: 619-20.

Withers, MR and BJ Funke, "Human Costs of Sanctions" [Letter], *N Engl J Med* 1997 Aug 28;337(9):643;

discussion 644.

Mullett, MD and MR Withers, "Physical Characteristics of Infant Endotracheal Tubes", *J Perinatol*, 1988:

124-6.

## 17.2 Appendix B: Overview of Study Operational Scheme

### **17.2.1 Operational Study Milestones**

All dates are provisional and are intended for planning purposes only.

Begin screening: 27 March 2003

Dose 1a: 28 April 2003

Dose 1b: 12 May 2003

Dose 1c: 26 May 2003

Dose 2a: 27 May 2003

Dose 2b: 9 June 2003

Dose 2c: 23 June 2003

Dose 3a: 24 June 2003

Dose 3b: 7 July 2003

Dose 3c: 21 July 2003

Begin post-immunization surveillance period: 26 August 2003

Interim Data Safety Report: Early October 2003

Final Data Safety Report: November 2004

### **Study Recruitment Plan**

One hundred thirty-five (135) healthy subjects aged 12 to 47 months will be recruited by non-coercive means according to existing U.S. Army regulations (AR 70-25 and AR 40-38).

The aims and objectives the study will be thoroughly discussed with local community leaders prior to the start date. A preliminary census of all of Kombewa District has been done (August - October 2001) in anticipation of malaria vaccine studies in this area.

| Table 14. A selected portion of the pediatric population of Kombewa District, Kisumu Division, Western Kenya. Only children living (1) within one mile of 22 proposed field stations (See Table 15 & attached maps) & (2) falling into three age groups (12-24, 24-36, & 36-48 months) are presented. These data are taken from a “Walter Reed Pro-ject” census performed by Mr Shadrack Odera & others from Aug to Oct 2001, and reflect the demographic reality at that time. The total adult & pediatric population of Kombewa District was found to be approximately 63,000. | | | | | |
| --- | --- | --- | --- | --- | --- |
| **Number of Family Compounds with** | | | | | |
|  | **1 Child** | **2 Children** | **3 Children** | **4 Children** | **Total** |
| 12-24 month olds | 1191  (1191 children) | 120  (240 children) | 14  (42 children) | 4  (16 children) | (1479 children) |
| 24-36 month olds | 1429  (1429 children) | 151  (302 children) | 25  (75 children) | 3  (12 children) | (1744 children) |
| 36-48 month olds | 1146  (1146 children) | 114  (228 children) | 13  (39 children) | 0  (0 children) | (1335 children) |
| All (12-48 month olds) | 3766  (3766 children) | 385  (770 children) | 52  (156 children) | 7  (28 children) | (4558 children) |

Prospective subjects that met age and other criteria one year ago have been identified as indicated in Table 14. Additionally, children in the ages of interest are indicated in Table 15, which summarizes the pediatric populations within one mile radius of 22 proposed field sites. As some prospective field sites have radii that overlap, these numbers are somewhat inflated; the grand total of 6,371 children shown (Table 15) is about 40% higher than the actual total of 4,558 (Table 14). Locations of Kombewa field stations are indicated on the map in Figure 1. Locations of children within the stated radii are shown, according to age group, in the maps in Figures 2, 3 and 4.

| Table 15. A selected portion of the pediatric population of Kombewa District, Kisumu Division, Western Kenya. Only those children living within one mile of 22 proposed field stations (See Table 14 & attached maps) & falling into three age groups are presented. The grand total number is about 28% inflated due to catchment overlap (i.e., some proposed field sites are less than a mile apart). | | | | | |
| --- | --- | --- | --- | --- | --- |
|  | **Field Stations** | Ages (months) | | | |
|  |  | 12-24 | 24-36 | 36-48 | Totals |
| 1. | WRP Kombewa Clinic | 155 | 159 | 132 | 446 |
| 2. | Abol Primary School | 116 | 173 | 117 | 406 |
| 3. | Lieye Primary School | 125 | 156 | 114 | 395 |
| 4. | Jonyo Primary School | 110 | 153 | 103 | 366 |
| 5. | Chwa Polytechnic School | 118 | 116 | 101 | 335 |
| 6. | Bar Korwa Primary School | 106 | 112 | 110 | 328 |
| 7. | Alwala Primary School | 103 | 120 | 92 | 315 |
| 8. | Rabongi Primary School | 90 | 118 | 98 | 306 |
| 9. | Rapogi Nursery School | 97 | 132 | 75 | 304 |
| 10. | Ngere Primary School | 102 | 115 | 73 | 290 |
| 11. | Nyaundi Primary School | 93 | 118 | 77 | 288 |
| 12. | Kitare Primary School | 89 | 108 | 88 | 285 |
| 13. | Kondik Market | 82 | 102 | 94 | 278 |
| 14. | Korumba Primary School | 93 | 93 | 72 | 258 |
| 15. | Nyawanga Primary School | 90 | 95 | 68 | 253 |
| 16. | Kolenyo Market | 74 | 89 | 76 | 239 |
| 17. | Ang’oga Market | 67 | 103 | 69 | 239 |
| 18. | Kopingo Market | 82 | 77 | 76 | 235 |
| 19. | Ranen Primary School | 82 | 86 | 64 | 232 |
| 20. | Oswre Primary School | 74 | 83 | 57 | 214 |
| 21. | Oruga Primary School | 62 | 82 | 57 | 201 |
| 22. | Ramuya Primary School | 46 | 73 | 39 | 158 |
|  | ***TOTALS*** | 2056 | 2463 | 1852 | 6371 |

At least one month prior to screening, the parental consent explanation forms will be distributed to the parents of prospective subjects and study objectives and procedures will be explained to them in their primary language via a videotaped presentation. Recruitment will be incremental until the desired number of subjects is enrolled. Subjects in each cohort will be randomized separately from the other cohorts, each cohort at the time of its first vaccination.

The three field sites (actually, the study center and 2 outlying field sites) indicated in Table 16 are proposed as the active sites for the present phase I trial. Coordination with community leaders in these areas has been ongoing and interest in the trial locally is both high and positive. These sites are identified provisionally and for planning purposes only.

| **Table 16: Three sites proposed as field stations for recruitment & subsequent follow-up for the 135 subjects in the present study** | | | | | |
| --- | --- | --- | --- | --- | --- |
|  | **Field Station** | **Location** | **Sub-Location** | **No. Children*** | **Long/Lat Coordinates** |
| 1. | WRP Kombewa Clinic | South Central Seme | Upper Kombewa | 446 | 00° 10’S/ 34°52’E |
| 2. | Bar Korwa Pri School | North Central Seme | East Katieno | 328 | 00° 07’S/ 34°51’E |
| 3. | Rapogi Primary School | West Seme | West Reru | 201 | 00° 09’S/ 34°45’E |
|  | **Total** |  |  | 975 |  |

* Number of children 12 – 48 months old within 1mile radius of field stations circa Oct 2001.

One hundred thirty-five children from 12 to 47 months of age will be recruited from the vicinities of Kombewa Town, Bar Korwa and Rapogi. Subject numbers will be evenly divided among the 3 year groups: 45 12-23 month olds, 45 24-35 month olds and 45 36-47 month olds. According to the 2001 WRP census, there were 155, 159 and 132 of these, respectively, residing within one mile of the clinic at Kombewa Town. The comp-arable numbers for Bar Korwa were 106, 112 and 110; and for Rapogi, 62, 82, and 57. Of course, the 12-23 month olds at these sites are now 24-36 month olds and the 24-35 month olds are 36-47 months old. These numbers, however, suggest that the pediatric population in these areas is more than adequate to support the number of subjects con-templated for the proposed study.

### **Study Operational Field Plan**

With 3 field stations in use for this phase I study, and 135 total subjects, approximately 45 subjects will be followed at each station. An effort will be made to select subjects in roughly these numbers from each site. While the monthly scheduled study visits are to be undertaken at the WRP Kombewa Clinic, it is anticipated that parents will need to bring a sick or symptomatic child to a field station at night or at other inconvenient times. Kombewa field stations are intended to be contact points for subjects and their parents with WRP staff and, as such, they have been selected with an eye to ease of foot travel. A parent with a child might reasonably be expected to walk a mile in 30 minutes or so, and thus, field sites were evaluated with this transit time in mind.

Kombewa field stations are intended to be occupied by a WRP field worker 24 hours per day throughout the study period and, as such, they will all represent a physically secure (lockable) room at a school, market or church and will be provisioned with basic overnight supplies, such as radio handset, mobile telephone (where service is available), basic medical kit (bandages, latex gloves), kerosene lantern, flashlight, batteries, raincoat, blanket, rubber boots, folding cot, mosquito net, insecticide spray and padlock with keys. At the WRP Kombewa Clinic a unit mobile phone battery will be available for field worker use.

Seven field workers will be assigned to each field station, necessitating the employment of 21 field workers for this study. This number gives a ratio of 6.3 subjects to each field worker.

On-duty field workers, while not usually medically trained personnel, are each answerable to not only a senior field worker, but to an on duty Clinical Officer (Kenyan equivalent of physician assistant) or to a physician. An on-call schedule will be devised, such that, with one worker on duty at the station and one worker off duty (recovering from night call the night before), a minimum of 5 workers are to be available for home visits on any given day (assuming no sick days are taken). New field workers will undergo a 2-3 week training period during which they will be exposed to essential medical aspects of the present study, the operational plan for the field workers involved in the study and especially, the logistics of field station duty (radio communication, trans-portation, etc).

Six 4WD ground vehicles will be permanently stationed at the WRP Kombewa Clinic; 5 to do twice daily visits to field stations involved in another, concurrent field study in Kombewa and one as a dedicated ambulance. All vehicles will be available for use in both studies. Ambulance drivers will be equipped with a dedicated vehicle radio and a cell phone. A senior, supervising field worker is on call each night as are either one of 2 physicians or one of 8 clinical officers who will rotate night call at the WRP Kombewa Clinic.

### **Generic Schedule of Events**

| Visit # | **Days & Dates** | **Approx.**  **Length of Visit** | Scheduled Activities |
| --- | --- | --- | --- |
| 1  (Screening Visit) |  | 2.5 - 3 hours | Briefing (Information presented about the study; discussion period afterwards for questions and answers)  Sign written consent form  Take vital signs (Temperature, heart rate, blood pressure); Weight check  Medical history obtained; Physical Examination performed  Blood drawn (1/2 teaspoonful) |
| Photo ID |  | 30 minutes | Photograph taken and Volunteer ID card provided |
| 2 |  | 3 hours | Take vital signs (Temperature, heart rate, blood pressure); Weight check  Recent medical history reviewed; brief physical examination; Review exclusion criteria  Blood drawn (1/2 teaspoonful)  Identity verified; Vaccination given  Observation for 60 minutes; Take vital signs; Assessment |
| 3 |  | 30 minutes | Take vital signs (Temperature, heart rate, blood pressure)  Assessment; examination of injection site |
| 4 |  | 30 minutes | Take vital signs (Temperature, heart rate, blood pressure)  Assessment; examination of injection site |
| 5 |  | 30 minutes | Take vital signs (Temperature, heart rate, blood pressure)  Assessment; examination of injection site |
| ***6*** |  | ***30 minutes*** | ***Take vital signs (Temperature, heart rate, blood pressure)***  ***Brief medical history and symptom-directed physical examination*** |
| 7 |  | 1 hour | Take vital signs (Temperature, heart rate, blood pressure); Weight check  Brief medical history and physical examination  Blood drawn (1/2 teaspoonful) |
| 8 |  | 3 hours | Take vital signs (Temperature, heart rate, blood pressure); Weight check  Recent medical history reviewed; brief physical examination  Blood drawn (1/2 teaspoonful)  Identity verified; Vaccination given  Observation for 60 minutes; Take vital signs; Assessment |
| 9 |  | 30 minutes | Take vital signs (Temperature, heart rate, blood pressure)  Assessment; examination of injection site |
| 10 |  | 30 minutes | Take vital signs (Temperature, heart rate, blood pressure)  Assessment; examination of injection site |
| 11 |  | 30 minutes | Take vital signs (Temperature, heart rate, blood pressure)  Assessment; examination of injection site |
| ***12*** |  | ***30 minutes*** | ***Take vital signs (Temperature, heart rate, blood pressure)***  ***Brief medical history and physical examination*** |
| 13 |  | 1 hour | Take vital signs (Temperature, heart rate, blood pressure); Weight check  Brief medical history and physical examination  Blood drawn (1/2 teaspoonful) |
| 14 |  | 3 hours | Take vital signs (Temperature, heart rate, blood pressure); Weight check  Recent medical history reviewed; brief physical examination  Blood drawn (1/2 teaspoonful)  Identity verified; Vaccination given  Observation for 60 minutes; Take vital signs; Assessment |
| 15 |  | 30 minutes | Take vital signs (Temperature, heart rate, blood pressure)  Assessment; examination of injection site |
| 16 |  | 30 minutes | Take vital signs (Temperature, heart rate, blood pressure)  Assessment; examination of injection site |

| Visit # | **Day and Date** | **Approx.**  **Length of Visit** | Scheduled Activities |
| --- | --- | --- | --- |
| 17 |  | 30 minutes | Take vital signs (Temperature, heart rate, blood pressure)  Assessment; examination of injection site |
| ***18*** |  | ***30 minutes*** | ***Take vital signs (Temperature, heart rate, blood pressure)***  ***Brief medical history and physical examination*** |
| 19 |  | 1 hour | Take vital signs (Temperature, heart rate, blood pressure); Weight check  Brief medical history and physical examination  Blood drawn (1/2 teaspoonful) |
| *20 |  | 1 hour | Take vital signs (Temperature, heart rate, blood pressure); Weight check  Brief medical history and physical examination  Blood drawn (1/2 teaspoonful) |
| 21 |  | 1 hour | Take vital signs (Temperature, heart rate, blood pressure); Weight check  Brief medical history and physical examination  Blood drawn (1/2 teaspoonful) |
| 22 |  | 1 hour | Take vital signs (Temperature, heart rate, blood pressure); Weight check  Brief medical history and physical examination  Blood drawn (1/2 teaspoonful) |
| 23 |  | 1 hour | Take vital signs (Temperature, heart rate, blood pressure); Weight check  Brief medical history and physical examination  Blood drawn (1/2 teaspoonful) |

After Visit # 20, a field worker will visit you once a month. The field worker will check to see whether you have changed where you live, and will remind you of your next clinic appointment.

***Important: If your child becomes ill at any time during this study, you should come in with the child to the clinic & be seen.***

### **Specific Daily Schedule of Events**

The dates on this schedule are for planning purposes and are provisional only. This sche-dule presupposes that Cohorts B and C will be available for vaccinations two weeks after previous doses are given. Any of these vaccination dates may change to somewhat later dates, however, in the event of a temporary discontinuation of vaccinations (postpone-ment may be for up to 5 weeks) or for other unforeseen reasons.

| **Study Day** | **Date**  **(2003)** | **Cohort A** | **Cohort B** | **Cohort C** | **Events** |
| --- | --- | --- | --- | --- | --- |
| -30/31 | 27-28 MAR | Visit 0 | Visit 0 | Visit 0 | Video & Consenting for Parents |
| -5/6 | 22-23 APR | Visit 1 | Visit 1 | Visit 1 | 1st Visits & Screenings of all Subjects |
| -3/4 | 24-25 APR |  |  |  | Notifications of Enrollment |
| -2 | 26 APR |  |  |  |  |
| -1 | 27 APR |  |  |  |  |
| 0 | 28 APR | Dose 1a  Visit 2 |  |  | Enrollment & 1st Vaccination of Cohort A |
| 1 | 29 APR | Visit 3 |  |  | Post-vaccination F/U visit for Cohort A |
| 2 | 30 APR | Visit 4 |  |  | Post-vaccination F/U visit for Cohort A |
| 3 | 01 MAY | Visit 5 |  |  | Post-vaccination F/U visit for Cohort A |
| 4 | 02 MAY |  |  |  |  |
| 5 | 03 MAY |  |  |  |  |
| 6 | 04 MAY |  |  |  |  |
| 7 | 05 MAY | Visit 6 |  |  | Day 7 post-vaccination F/U visit for Cohort A |
| 8 | 06 MAY |  |  |  | Dose 1a AE Report to DSMB & LMM |
| 9 | 07 MAY |  |  |  |  |
| 10 | 08 MAY |  |  |  |  |
| 11 | 09 MAY |  |  |  |  |
| 12 | 10 MAY |  |  |  |  |
| 13 | 11 MAY |  |  |  |  |
| 14 | 12 MAY |  | Dose 1b  Visit 2 |  | Enrollment & 1st Vaccination of Cohort B  (Requirement: Formal DSMB/LMM concurrence) |
| 15 | 13 MAY | Visit 7 | Visit 3 |  | Day 15 post-vaccination F/U visit for Cohort A; Post-vaccination F/U visit for Cohort B |
| 16 | 14 MAY |  | Visit 4 |  | Post-vaccination F/U visit for Cohort B |
| 17 | 15 MAY |  | Visit 5 |  | Post-vaccination F/U visit for Cohort B |
| 18 | 16 MAY |  |  |  |  |
| 19 | 17 MAY |  |  |  |  |
| 20 | 18 MAY |  |  |  |  |
| 21 | 19 MAY |  | Visit 6 |  | Day 7 post-vaccination F/U visit for Cohort B |
| 22 | 20 MAY |  |  |  | Dose 1b AE Report to DSMB & LMM |
| 23 | 21 MAY |  |  |  |  |
| 24 | 22 MAY |  |  |  |  |
| 25 | 23 MAY |  |  |  |  |
| 26 | 24 MAY |  |  |  |  |
| 27 | 25 MAY |  |  |  |  |
| 28 | 26 MAY |  | Visit 7 | Dose 1c  Visit 2 | Enrollment & 1st Vaccination of Cohort C  (Requirement: Formal DSMB/LMM concurrence)  Day 14 post-vaccination F/U visit for Cohort B |
| 29 | 27 MAY | Dose 2a  Visit 8 |  | Visit 3 | 2nd vaccination of Cohort A  Post-vaccination F/U visit for Cohort C |
| 30 | 28 MAY | Visit 9 |  | Visit 4 | Post-vaccination F/U visit for Cohorts A & C |
| 31 | 29 MAY | Visit 10 |  | Visit 5 | Post-vaccination F/U visit for Cohorts A & C |
| 32 | 30 MAY | Visit 11 |  |  | Post-vaccination F/U visit for Cohort A |
| 33 | 31 MAY |  |  |  |  |
| 34 | 01 JUN |  |  |  |  |
| 35 | 02 JUN |  |  | Visit 6 | Day 7 post-vaccination F/U visit for Cohort C; LMM Report: Solicited AEs (NB: Report for both 1c & 2a) |
| 36 | 03 JUN | Visit 12 |  |  | Day 7 post-vaccination F/U visit for Cohort A; Dose 1c AE Report to LMM |
| 37 | 04 JUN |  |  |  | Dose 2a AE Report to LMM |
| 38 | 05 JUN |  |  |  |  |
| 39 | 06 JUN |  |  |  |  |
| 40 | 07 JUN |  |  |  |  |
| 41 | 08 JUN |  |  |  |  |
| 42 | 09 JUN |  | Dose 2b  Visit 8 | Visit 7 | 2^nd^ vaccination of Cohort B  Day 14 post-vaccination F/U visit for Cohort C |
| 43 | 10 JUN | Visit 13 | Visit 9 |  | Day 14 post-vaccination F/U visit for Cohort A; Post-vaccination F/U visit for Cohort B |
| 44 | 11 JUN |  | Visit 10 |  | Post-vaccination F/U visit for Cohort B |
| 45 | 12 JUN |  | Visit 11 |  | Post-vaccination F/U visit for Cohort B |
| 46 | 13 JUN |  |  |  |  |
| 47 | 14 JUN |  |  |  |  |
| 48 | 15 JUN |  |  |  |  |
| 49 | 16 JUN |  | Visit 12 |  | Day 7 post-vaccination F/U visit for Cohort B |
| 50 | 17 JUN |  |  |  | Dose 2b AE Report to LMM |
| 51 | 18 JUN |  |  |  |  |
| 52 | 19 JUN |  |  |  |  |
| 53 | 20 JUN |  |  |  |  |
| 54 | 21 JUN |  |  |  |  |
| 55 | 22 JUN |  |  |  |  |
| 56 | 23 JUN |  |  | Dose 2c  Visit 8 | 2^nd^ vaccination of Cohort C |
| 57 | 24 JUN | Dose 3a  Visit 14 |  | Visit 9 | 3^rd^ Vaccination of Cohort A  Post-vaccination F/U visit for Cohort C |
| 58 | 25 JUN | Visit 15 | Visit 13 | Visit 10 | Post-vaccination F/U visit for Cohorts A & C; Day 16 post-vaccination F/U visit for Cohort B |
| 59 | 26 JUN | Visit 16 |  | Visit 11 | Post-vaccination F/U visit for Cohorts A & C |
| 60 | 27 JUN | Visit 17 |  |  | Post-vaccination F/U visit for Cohort A |
| 61 | 28 JUN |  |  |  |  |
| 62 | 29 JUN |  |  |  |  |
| 63 | 30 JUN | Visit 18 |  |  | Day 7 post-vaccination F/U visit for Cohort C; LMM Report: Solicited AEs |
| 64 | 01 JUL |  | Visit 14 | Visit 12 | Day 7 post-vaccination F/U visit for Cohort A; Dose 2c AE Report to LMM |
| 65 | 02 JUL |  |  |  | Dose 3a AE Report to LMM |
| 66 | 03 JUL |  |  |  |  |
| 67 | 04 JUL |  |  |  |  |
| 68 | 05 JUL |  |  |  |  |
| 69 | 06 JUL |  |  |  |  |
| 70 | 07 JUL | Visit 19 | Dose 3b  Visit 15 |  | 3^rd^ Vaccination of Cohort B  Day 14 post-vaccination F/U visit for Cohort C; LMM Report: Solicited AEs |
| 71 | 08 JUL |  | Visit 16 | Visit 13 | Post-vaccination F/U visit for Cohort B;  Day 14 post-vaccination F/U visit for Cohort A |
| 72 | 09 JUL |  | Visit 17 |  | Post-vaccination F/U visit for Cohort B |
| 73 | 10 JUL |  | Visit 18 |  | Post-vaccination F/U visit for Cohort B |
| 74 | 11 JUL |  |  |  |  |
| 75 | 12 JUL |  |  |  |  |
| 76 | 13 JUL |  |  |  |  |
| 77 | 14 JUL |  |  |  | Day 7 post-vaccination F/U visit for Cohort B |
| 78 | 15 JUL |  |  |  | Dose 3b AE Report to LMM |
| 79 | 16 JUL |  |  |  |  |
| 80 | 17 JUL |  |  |  |  |
| 81 | 18 JUL |  |  |  |  |
| 82 | 19 JUL |  |  |  |  |
| 83 | 20 JUL |  |  |  |  |
| 84 | 21 JUL |  |  | Dose 3c  Visit 14 | 3^rd^ Vaccination of Cohort C;  LMM Report: Solicited AEs |
| 85 | 22 JUL |  | Visit 19 | Visit 15 | Post-vaccination F/U visit for Cohort C; Day 15 post-vaccination F/U visit for Cohort B |
| 86 | 23 JUL |  |  | Visit 16 | Post-vaccination F/U visit for Cohort C |
| 87 | 24 JUL | Visit 20 |  | Visit 17 | Post-vaccination F/U visit for Cohort C  Day 30 post-vaccination F/U visit for Cohort A |
| 88 | 25 JUN |  |  |  |  |
| 89 | 26 JUL |  |  |  |  |
| 90 | 27 JUL |  |  |  |  |
| 91 | 28 JUL |  |  | Visit 18 | Day 7 post-vaccination F/U visit for Cohort C; DSMB (& LMM) Report: Solicited AEs |
| 92 | 29 JUL |  |  |  | Dose 3c AE Report to LMM |
| 93 | 30 JUL |  |  |  |  |
| 94 | 31 JUL |  |  |  |  |
| 95 | 01 AUG |  |  |  |  |
| 96 | 02 AUG |  |  |  |  |
| 97 | 03 AUG |  |  |  |  |
| 98 | 04 AUG |  |  |  | Day 14 post-vaccination F/U visit for Cohort C |
| 99 | 05 AUG |  |  | Visit 19 | Day 14 post-vaccination F/U visit for Cohort C |
| 100 | 06 AUG |  | Visit 20 |  | Day 30 post-vaccination F/U visit for Cohort B |
| 101 | 07 AUG |  |  |  |  |
| 102 | 08 AUG |  |  |  |  |
| 103 | 09 AUG |  |  |  |  |
| 104 | 10 AUG |  |  |  |  |
| 105 | 11 AUG |  |  |  |  |
| 106 | 12 AUG |  |  |  |  |
| 107 | 13 AUG |  |  |  |  |
| 108 | 14 AUG |  |  |  |  |
| 109 | 15 AUG |  |  |  |  |
| 110 | 16 AUG |  |  |  |  |
| 111 | 17 AUG |  |  |  |  |
| 112 | 18 AUG |  |  |  |  |
| 113 | 19 AUG |  |  |  |  |
| 114 | 20 AUG |  |  | Visit 20 | Day 30 post-vaccination F/U visit for Cohort C |
| 115 | 21 AUG |  |  |  |  |
| 116 | 22 AUG |  |  |  |  |
| 117 | 23 AUG |  |  |  |  |
| 118 | 24 AUG |  |  |  |  |
| 119 | 25 AUG |  |  |  |  |
| 120 | 26 AUG |  |  |  | End “Follow-up Period for Unsolicited AEs”; Begin “Post-vaccination safety surveillance period for SAEs” (To Study day 240) |
| 121 | 27 AUG |  |  |  |  |
| 122 | 28 AUG |  |  |  |  |
| 123 | 29 AUG |  |  |  |  |
| 124 | 30 AUG |  |  |  |  |
| 125 | 31 AUG |  |  |  |  |
| 126 | 01 SEP |  |  |  |  |
|  |  |  |  |  |  |
| 144 | 19 SEP |  |  |  | Partial (“by group”) unblinding of study data  (Or 30 days after the 30 day post-vaccination F/U visit for Cohort C’s 3^rd^ dose) |
|  |  |  |  |  |  |
| 156 | 01 OCT |  |  |  |  |
| 157 | 03 OCT |  |  |  | Interim Safety Data Report (from SCI) |
|  |  |  |  |  |  |
| 187 | 01 NOV |  |  |  |  |
|  |  |  |  |  |  |
| 217 | 01 DEC |  |  |  |  |
|  |  |  |  |  |  |
| 239 | 23 DEC |  |  |  | End post-vaccination safety surveillance period for SAEs |
| 240 | 24 DEC |  |  |  | Begin long-term follow-up period for malaria & immunogenicity (Study days 240-364);  Formal full (“by individual”) study unblinding |
| 248 | 01 JAN 04 |  |  |  |  |
|  |  |  |  |  |  |
| 279 | 01 FEB 04 |  |  |  |  |
|  |  |  |  |  |  |
| 307 | 29 FEB |  |  |  | Leap Day! |
| 308 | 01 MAR |  |  |  |  |
|  |  |  |  |  |  |
| 339 | 01 APR 04 |  |  |  |  |
|  |  |  |  |  |  |
| 364 | 26 APR 04 |  |  |  | End long-term follow-up period for malaria & immunogenicity |

| ??? | NOV 04 |  |  |  | Final Safety Data Report |
| --- | --- | --- | --- | --- | --- |

## 17.3 Appendix C: Administrative Matters

### **17.3.1 General Administrative Matters**

I. Responsibilities of the Investigator

· To ensure that he/she has sufficient time to conduct and complete the study and has adequate staff and appropriate facilities which are available for the duration of the study and to ensure that other studies do not divert essential subjects or facilities away from the study at hand.

· To submit an up-to-date curriculum vitae and other credentials (e.g. medical license number in the United States) to the sponsor and—where required—to relevant authorities.

· To acquire the normal ranges for laboratory tests performed locally and, if required by local regulations, obtain the Laboratory License or Certification.

· To prepare and maintain adequate case histories designed to record observations and other data pertinent to the study.

· To conduct the study in compliance with the protocol and appendices (Section 17).

· To cooperate with a representative of the sponsor in the monitoring process of the study and in resolution of queries about the data.

II. Protocol Amendments & modifications

No changes to the study protocol will be allowed unless discussed in detail with the sponsor and filed as an amendment/modification to this protocol.

Any amendment/modification to the protocol will be adhered to by the participating center and will apply to all subjects. Written IRB/IEC approval of protocol amendments is required prior to implementation. All amendments will be submitted to the HSRRB through Office of Research Management, and to the KEMRI Ethical Review Committee, and to PATH HSPC. No amendments will go into effect without written approval from HSRRB, KEMRI ERC, and PATH HSPC except when the amendments are purely of an administrative nature or when the changes are necessary to eliminate immediate hazards to the subjects.

This study is to be conducted in accordance with 21 CFR 56, which requires that changes in approved research may not be initiated without IRB review and approval except where necessary to remove apparent immediate hazards to the human research subject.

III. Sponsor’s Termination of Study

The sponsor reserves the right to discontinue the clinical study at any time for medical or administrative reasons. When feasible, a 30-day written notification will be tendered.

1. Case Report Form Instructions

Prior to screening the first potential subject, the investigator will provide a list showing the signature and hand-written initials of all individuals authorized to make or change entries on case report forms (CRFs). If the authorized individuals should change during the study, the investigator is to inform the sponsor.

CRFs will be supplied by Statistics Collaborative, Inc., for recording all data. It is the responsibility of the investigator or co-investigator to ensure that CRFs are legible and completely filled in with a black ink fountain or ballpoint pen.

Errors must be corrected by drawing a single line through the incorrect entry and writing in the new value/data positioned as close to the original as possible. The correction must then be initialed, dated and justified, where necessary, by the authorized individual making the change. The original entry must not be obliterated, overwritten or erased when a correction is made.

Every effort will be made by the investigator or designated staff to complete the relevant sections of the case report as soon as feasible following a visit. Similarly, when a subject completes the study, every effort will be made to complete the CRF as soon as the last data become available.

As soon as the subject has completed/withdrawn from the study and the case report form is completed the principal investigator or designated physician(s) under his/her super-vision will sign the study conclusion pages of the source document to confirm that they have reviewed the data and that the data are completed and accurate.

An original (top copy) case report form or log sheets must be submitted for all subjects who have undergone protocol specific procedures, whether or not the subject completed the study.

While completed CRFs will be reviewed by a professional monitor at the study site, errors detected by subsequent in-house case report form review may necessitate clarification or correction of errors and documentation and approval by the investigator. Whenever possible the investigator should assist in clarification or correction of errors detected after study finalization within 48 hours of them being brought to the attention of the investigator.

Any questions or comments related to the CRFs should be directed to the assigned Site Monitor.

Monitoring by USAMMDA (i.e. “the Sponsor”)

See Section 17.3.2.I “Monitoring/Quality Assurance”.

VI. Archiving of Data

The investigator/ institution should maintain all study documentation until at least 2 years after the last approval of a marketing application in an ICH region and until there are no pending or contemplated marketing applications in an ICH region or at least 2 years have elapsed since the formal discontinuation of the clinical development of the investigational product. These documents should be retained for a longer period however if required by the applicable regulatory requirements or by an agreement with the sponsor. It is the responsibility of the sponsor to inform the investigator/institution as to when these documents no longer need to be retained. The investigator/ institution should take measures to prevent accidental or premature destruction of these documents.

Similarly, the sponsor-specific study documentation should be retained until at least 2 years after the last approval of a marketing application in an ICH region and until there are no pending or contemplated marketing applications in an ICH region or at least 2 years have elapsed since the formal discontinuation of clinical development of the invest-igational product. These documents should be retained for a longer period however if required by the applicable regulatory requirements or if needed by the sponsor. The sponsor should inform the investigator/institution in writing of the need for record retent-ion and should notify the investigator/institution in writing when the study-related records are no longer needed.

VII. Audits

For the purpose of compliance with Good Clinical Practice and Regulatory Agency Guidelines it may be necessary for a Drug Regulatory Agency to conduct a site audit. This may occur at any time from start to after conclusion of the study.

When an investigator signs the protocol, he agrees to permit Drug Regulatory Agencies and the sponsor access to source data/ documents. Furthermore, if an investigator refuses an inspection, his data will not be accepted in support of a New Drug Registration and/or Application.

The Inspector will be especially interested in the following items:

· Log of visits from the sponsor's representatives

· IRB/IEC approval

· Vaccine accountability

· Approved study protocol and amendments

· Informed consent of the subjects (written or witnessed oral consent)

· Medical records supportive of case report form data

· Reports to the IRB/IEC and the sponsor

· Record retention

### **17.3.2 USAMRMC Specific Administrative Procedures**

I Monitoring/Quality Assurance

Monitoring of this protocol will be performed by representatives of the USAMMDA Office of Quality Assurance, and GSK Biologicals. Study monitors will conduct a study initiation visit (study day 0), mid-study visits at regular intervals, and a study close-out visit soon after the conclusion of the study (approximately month 13). The monitor will review CRFs and will compare them against source documents to verify accurate data collection, to evaluate adherence to Good Clinical Practices, and to ensure completeness, accuracy, and integrity of study data. The investigator must ensure provision of reason-able time, space and adequate qualified personnel for monitoring visits.

Copies of all regulatory documents will be on file in the Walter Reed Project Field Laboratory in Kisian. Source documents, will be archived in the Walter Reed Project Kombewa Clinic or at a storage facility in nearby Kisumu unless other long-term storage options are subsequently defined. In addition, documentation of test article storage, inventory, and accountability will be maintained at the clinical site.

II Evaluations During and Following the Project

The medical evaluations of subjects will be recorded by one of the physician investigat-ors on standard forms. Blood samples for antibody tests will be obtained by approp-riately trained individuals. Consent forms along with a copy of the final approved proto-col will be retained indefinitely. The volunteer registry data sheets (VDRSs) will be collected and submitted to the Office of Regulatory Compliance and Quality (RCQ) at the conclusion of the study (after unblinding). RCQ then maintains the information in a database allowing subjects to be contacted regarding new information about the vaccine as it becomes available. This database is maintained for a minimum of 75 years.

III Withdrawal from Protocol for Individual Subjects

Subjects will be allowed to withdraw from the study at any time.

IV Modification of the Protocol

Any required protocol ***modifications*** will be submitted in writing to the WRAIR Office of Research Management (ORM) and the KEMRI Scientific Steering Committee (SSC) and to IRBs of the participating facilities and to the Office of Regulatory Compliance and Quality, Office of the Surgeon General, US Army. Protocol ***amendments*** must be reviewed and approved by the KEMRI ERC, the WRAIR ORM, and, where appropriate, by the WRAIR Scientific Review Committee, PATH HSPC, GSK Biologicals and HSRRB prior to implementation. ***Administrative changes*** to the protocol must be submitted to the KEMRI ERC, MVI/PATH, and HSRRB. If required, subjects will be provided with a revised informed consent document for their signature.

V Disposition of Unused Medications

Unused investigational vaccine doses will be accounted for and will be returned to the manufacturer for safekeeping or disposed of according to the manufacturer’s policy.

VI Use of Information and Publications Arising from this Study

It is anticipated that the results of this study will be presented to the scientific community via oral presentations at meetings and written publications in scientific journals. The official final report will be submitted through appropriate channels and upon approval by the WRAIR, Dept. of Immunology to the local IRB, the Regulatory Affairs Branch of RCQ and to the HSRRB. This report will contain detailed information about the subjects, their tolerance of the vaccines, their side effects and laboratory abnormalities, as well as their overall immune responses to immunization.

## 17.4 Appendix D: Parental Informed Consent

### **MSP-1 Malaria Vaccine (FMP-1) Study Recruitment Script**

# MSP-1 Malaria Vaccine (FMP-1) Study

Recruitment Script

Dr. Mark Withers

We are from the Walter Reed Project, Kombewa clinic based in Kombam village. We are about to begin a study to test an investigational vaccine against malaria. The study will last for 12 months and will involve a total of 23 clinic visits. During three of these visits, there will be injections. Participants will also be required to give about one-half teaspoon of blood a total of 11 times for the duration of the study.

Parent’s Name: _______________________________Compound Number: ____________

Village: ______________________ Child’s Age: _________ Child’s Sex: _________

Which language(s) do you best understand?

Luo

|  |
| --- |

Kiswahili

|  |
| --- |

English

|  |
| --- |

# Part 1: Please check the appropriate response

| Question | Yes | No |
| --- | --- | --- |
| 1. Is your child between the ages of 12 and 47 months? |  |  |
| 2. Do you plan to remain in the area for the next 12 months? |  |  |
| 3. Are you willing to attend a briefing session at WRP Kombewa? |  |  |

# Part 2: Briefing day and times Please check ONE appointment only *

Day Date Time Day Date Time

|  |  | 10.00 am | 2.00 pm |  |  | 10:00 am | 2:00 pm |
| --- | --- | --- | --- | --- | --- | --- | --- |
| Monday |  |  |  | Monday |  |  |  |
| Tuesday |  |  |  | Tuesday |  |  |  |
| Wednesday |  |  |  | Wednesday |  |  |  |
| Thursday |  |  |  | Thursday |  |  |  |
| Friday |  |  |  | Friday |  |  |  |

Field Worker’s Signature: ________________________________________________

Date: _________________________________

### **Parental Informed Consent Explanation**

(To be translated, read, and questions answered in a language in which the participant’s parent or legal representative is fluent.)

**TITLE OF STUDY:** A double blind, randomized, controlled, dose escalation phase I field trial in 12-47 month old children in western Kenya to evaluate the safety and immunogenicity of WRAIR’s MSP-1 (FMP-1) malaria vaccine adjuvanted in GlaxoSmithKline’s Biological’s AS02A versus rabies vaccine

**INSTITUTIONS:** Kenya Medical Research Institute, Walter Reed Project, Nairobi; Walter Reed Army Institute of Research, Washington D.C., Malaria Vaccine Initiative at the Program for Appropriate Technology in Health (PATH), and the US Agency for International Development. Collaborators: GlaxoSmithKline Biologicals.

**PRINCIPAL INVESTIGATOR:** Dr Mark R. Withers, Visiting Scientist, Kenya Medical Research Institute and Walter Reed Project, Kisumu, Kenya P.O. Box 54, Tel 254-57-22942.

**PARTICIPATION INFORMATION:** We would like you to allow your child to participate in a medical research study. It is important that you understand the following information that applies to all participants in our studies: 1) Participation is entirely voluntary. 2) You may withdraw your child from participation in this study, or any part of the study, at any time. Refusal to allow your child to participate will involve no penalty **or** loss of benefits to which your child is otherwise entitled. If you decide to withdraw your child from the study later, please inform any member of the Walter Reed Project Kombewa Clinic staff or make an appointment with the Principal Investigator listed above. 3) After you read the explanation, please feel free to ask any questions that will allow you to understand clearly the nature of the study. You will be informed of any new findings that may affect your willingness to allow your child to continue in the study.

**INTRODUCTION:** Malaria is a disease that affects many people throughout the tropics, including Kenya. It is caused by parasites that are transmitted by mosquito bites. Investigators at Walter Reed Army Institute of Research in collaboration with GlaxoSmithKline Biologicals, Belgium have developed an experimental vaccine against malaria called FMP-1. Preliminary tests of this vaccine in over 60 adults in the United States and 40 adults in Kenya have shown that this vaccine is safe. We would now like to test the FMP-1 malaria vaccine in children in western Kenya to make sure that it is safe to give to children who have already been exposed to malaria. To do this, we will compare its safety to another vaccine that is already being used, the rabies vaccine.

**PROCEDURES TO BE FOLLOWED:** If you decide to allow your child to participate in this research study, we will set a date for you to bring your child back for an appointment with one of the study doctors. During that appointment, we will ask you about your child’s medical history, and your child will be given a physical examination. We will draw less than a teaspoon of blood from your child in order to evaluate the health of your child for the study. It will take about one week to know the results of the examination and blood tests. If we find anything wrong that will not allow your child to participate in the study, you will be informed and your child will be referred to the Kombewa Sub-District Hospital for evaluation.

If your child’s exams are good, and if you agree to allow your child to continue, your child will be scheduled to receive 3 doses of either the experimental malaria vaccine or rabies vaccine at a later date. Prior to receiving the first dose of the vaccine, a picture will be taken of you and your child in order to create an identification card. You will need to bring this card with you whenever you bring your child to the clinic to be seen. Your child will have a 2 out of 3 chance of receiving the malaria vaccine and a 1 out of 3 chance of receiving the rabies vaccine. Neither you nor the doctors in the study will know which vaccine your child has received until the end of the study. This is done to make sure that the doctors evaluate your child honestly.

Once the first dose of vaccine is given, the second dose will be given about 30 days later and the third dose about 30 days after the second one. The vaccine will be given by injection into the muscle of one of your child’s legs. After each injection, you will be asked to stay in the clinic with your child for 60 minutes so that we may observe your child. After each injection, you will also need to bring your child back after 1, 2, 3, 7 and 14 days so that we can see how your child is feeling. Following the last dose of vaccine, a field worker will visit you at your home once every month. During the last six months of the study you will be asked to bring your child to the clinic every 3 months for evaluation.

During the study, we will need to draw blood from your child a total of 11 times. Less than a teaspoon of blood will be drawn from your child during each of these visits. This will be done to make sure that your child remains healthy and to measure the effect of the vaccine. Some of these samples may be stored indefinitely to do other tests that are not anticipated at this time. If such use is contemplated, the investigators will first seek approval from the KEMRI Ethical Review Committee. The total amount of blood required by the study will be about 1 1/2 tablespoons. If your child becomes ill, we may need to draw additional blood from your child to run further tests. If your child becomes ill from malaria, treatment will be provided.

**RISKS:** Minor bruising may appear at the site where blood is drawn. If your child receives the rabies vaccine, you should expect soreness, swelling and, in some cases, redness at the site of injection that will disappear after about 2 to 3 days. In addition, muscle soreness, itching, irritability or fussiness, low-grade fever, fatigue, headache, nausea and dizziness are possible during the same period of time. If any of these symptoms occur, we may give your child medication to help provide relief. As with any vaccine, there is a small possibility that your child may have an allergic reaction due to vaccination. The same symptoms may occur if your child receives the malaria vaccine. These reactions may be mild, such as a rash, or may be severe and life threatening. A life threatening reaction is extremely rare (the chances are about one in a million for commonly used vaccines, such as rabies vaccine). The injection is likely to result in a few days of local pain and temporary leg motion limitation (which requires use of analgesics) in more than half of the recipients. Allergic reactions to the some of the medications used in this study are also possible. There may be other reactions to the malaria vaccine that at this time are not known. If new information about the safety of the vaccine becomes available, you will be informed.

**DURATION OF SUBJECT'S PARTICIPATION**: 12 months

**BENEFITS:** Your child will receive follow-up medical care for 12 months at the Walter Reed Project Clinic located in the village of Kombewa, Kombewa Division. This care will include treatment of any vaccine side-effects, such as pain or swelling, with analgesics or other treatments. Treatment of your child for malaria and for other illnesses will be free of charge. Children who received the malaria vaccine will also be offered the opportunity to receive rabies immunization at the conclusion of the study. Transportation between field stations and the Kombewa Clinic for medical treatments and study visits will be provided for you and your child free of charge. However, neither you nor your child will receive any direct payment for participating in this study.

**DISCOMFORTS:** There is the possibility of mild discomfort at the site from where blood is obtained. As mentioned above, swelling, soreness and redness should be expected at the site of vaccine injection.

**PRECAUTIONS TO FOLLOW:** Once a child is evaluated for possible entry into the study, with the exception of true emergencies, medical care should be sought only through the WRP Kombewa Clinic, and no medications should be given to the child unless provided by the clinic. This should continue until you have been told that your child is not eligible or cannot enroll in the study. Please note: The malaria vaccine has **not** been proven to prevent malaria and has not been approved for use in the United States. Therefore, you should continue to practice your regular malaria prevention methods, including having your child sleep under a bed net. The malaria vaccine itself cannot cause malaria, and your child may not be protected by the vaccine from a natural infection with malaria.

**NUMBER OF PARTICIPANTS IN THE STUDY**: 135.

**ASSURANCE OF CONFIDENTIALITY OF PARTICIPANT’S IDENTITY:** Information about your child’s participation in this study will remain confidential. In any reports, subjects will referred to by study number only. Access to study files will be limited to members of the WRP staff only and all files will be kept in locked cabinets. The information collected may be reviewed by representatives of the Kenya Medical Research Institute, the U.S. Army Medical Research and Materiel Command (USAMRMC), the US Food and Drug Administration, PATH, and the study sponsor as part of their responsibility to oversee research. You will receive a copy of this consent form. By policy of the USAMRMC, your child’s name, address, the name of the study, and the dates of your child’s participation will be kept in records to easily identify people participating in research sponsored by USAMRMC and to make sure that you are adequately informed of risks and new information about the vaccine as it becomes available. The information we collect will be stored for a minimum of 75 years.

**CIRCUMSTANCES UNDER WHICH YOUR CHILD’S PARTICIPATION MAY BE TERMINATED WITHOUT YOUR CONSENT:**

The study doctor may decide that your child should no longer take part in this study due to:

1. Health conditions that might make participation dangerous to your child’s health.

2. Any other conditions that might make continued participation dangerous to your child’s health.

**MEDICAL CARE FOR INJURY OR ILLNESS:** Your child has the right to medical care at no cost for any injury or illness which occurs as a direct result of participation in this research project. You will not receive any money as payment for any injury or illness, only medical care for your child.

**PERSONS AND PLACES FOR ANSWERS IN THE EVENT OF RESEARCH RELATED INJURY:** If you think your child has a medical problem, please report to the nearest WRP field station or directly to the Walter Reed Project Kombewa Clinic, near Kombewa Town. If for some reason this is not possible, contact Dr. Mark R. Withers, Walter Reed Project, P.O. Box 54, Kisumu, Kenya Tel. 057-22942.

**ALTERNATIVES TO STUDY PARTICIPATION:** The only alternative to participation in this research study is not to participate in the study.

**FOR INFORMATION OR ANSWERS TO QUESTIONS CONCERNING YOUR RIGHTS AS A RESEARCH PARTICIPANT YOU MAY CONTACT:** The Chairman of the Kenya National Ethical Review Committee, C/O Kenya Medical Research Institute, P.O. Box 54840, Nairobi, Kenya Tel. 02-722541.

**IF THERE IS ANY PORTION OF THIS CONSENT EXPLANATION SHEET THAT YOU DO NOT UNDERSTAND, ASK THE INVESTIGATOR BEFORE SIGNING.**

### **17.4.2 Parental Schedule of Events**

| **Visit #** | **Day and Date** | **Approx.**  **Length of Visit** | **Scheduled Activities** | | |
| --- | --- | --- | --- | --- | --- |
| Briefing Day |  | 2.5 - 3 hours | Briefing (Information presented about the study; discussion period afterwards for questions and answers). Sign written consent form | | |
| 1  (Screening Visit) |  | 2.5 - 3 hours | Take vital signs (Temperature, heart rate, blood pressure). Medical history obtained. Physical Examination performed. Blood drawn (1/2 teaspoonful). Weight check. | | |
| Photo ID |  | 30 minutes | Photograph taken and Volunteer ID card provided | | |
| 2 |  | 3 hours | Take vital signs (Temperature, heart rate, blood pressure). Recent medical history reviewed. Brief physical examination. Blood drawn (1/2 teaspoonful). ***Vaccination given***. Observation for 60 minutes. Take vital signs & check weight. Assessment. | | |
| 3 |  | 30 minutes | Take vital signs (Temperature, heart rate, blood pressure); Assessment; examination of injection site | | |
| 4 |  | 30 minutes | Take vital signs (Temperature, heart rate, blood pressure)  Assessment; examination of injection site | | |
| 5 |  | 30 minutes | Take vital signs (Temperature, heart rate, blood pressure)  Assessment; examination of injection site | | |
| 6 |  | 30 minutes | Take vital signs (Temperature, heart rate, blood pressure); Assessment; examination of injection site | | |
| 7 |  | 1 hour | Take vital signs (Temperature, heart rate, blood pressure). Weight check. Brief medical history and physical examination. Blood drawn (1/2 teaspoonful) | | |
| 8 |  | 3 hours | Take vital signs (Temperature, heart rate, blood pressure). Weight check. Recent medical history reviewed. Brief physical examination. Blood drawn (1/2 teaspoonful). ***Vaccination given***. Observation for 60 minutes. Take vital signs. Assessment. | | |
| 9 |  | 30 minutes | Take vital signs (Temperature, heart rate, blood pressure); Assessment; examination of injection site | | |
| 10 |  | 30 minutes | Take vital signs (Temperature, heart rate, blood pressure); Assessment; examination of injection site | | |
| 11 |  | 30 minutes | Take vital signs (Temperature, heart rate, blood pressure); Assessment; examination of injection site | | |
| 12 |  | 30 minutes | Take vital signs (Temperature, heart rate, blood pressure); Assessment; examination of injection site | | |
| 13 |  | 1 hour | Take vital signs (Temperature, heart rate, blood pressure). Weight check. Brief medical history and physical examination. Blood drawn (1/2 teaspoonful) | | |
| 14 |  | 3 hours | Take vital signs (Temperature, heart rate, blood pressure). Weight check. Recent medical history reviewed. Brief physical examination. Blood drawn (1/2 teaspoonful). ***Vaccination given***. Observation for 60 minutes. Take vital signs. Assessment. | | |
| 15 |  | 30 minutes | Take vital signs (Temperature, heart rate, blood pressure); Assessment; Examination of injection site | | |
| 16 |  | 30 minutes | Take vital signs (Temperature, heart rate, blood pressure); Assessment; examination of injection site | | |
| 17 |  | 30 minutes | Take vital signs (Temperature, heart rate, blood pressure); Assessment; examination of injection site | |  |
| 18 |  | 30 minutes | Take vital signs (Temperature, heart rate, blood pressure); Assessment; examination of injection site |  | |
| 19 |  | 1 hour | Take vital signs (Temperature, heart rate, blood pressure). Weight check. Brief medical history and physical examination. Blood drawn (1/2 teaspoonful) |  | |
| *20 |  | 1 hour | Take vital signs (Temperature, heart rate, blood pressure). Weight check. Brief medical history and physical examination. Blood drawn (1/2 teaspoonful) |  | |
| 21 |  | 1 hour | Take vital signs (Temperature, heart rate, blood pressure). Weight check. Brief medical history and physical examination. Blood drawn (1/2 teaspoonful) |  | |
| 22 |  | 1 hour | Take vital signs (Temperature, heart rate, blood pressure). Weight check. Brief medical history and physical examination. Blood drawn (1/2 teaspoonful) |  | |
| 23 |  | 1 hour | Take vital signs (Temperature, heart rate, blood pressure). Weight check. Brief medical history and physical examination. Blood drawn (1/2 teaspoonful) |  | |

After Visit # 20, a field worker will visit you once a month. The field worker will check to see whether you have changed where you live, and will remind you of your next clinic appointment.

***Important: If your child becomes ill at any time during this study, he or she should be brought to the WRP Kombewa Clinic to be seen. The clinic is staffed 24 hours a day, 7 days a week.***

### **17.4.3 Informed Consent Agreement**

**Study Title**: A double blind, randomized, controlled, dose escalation phase I field trial in 12 to 47 month old children in western Kenya to evaluate the safety and immunogenicity of WRAIR’s MSP-1 (FMP-1) malaria vaccine adjuvanted in GlaxoSmithKline Biologicals’ AS02A versus rabies vaccine

**Study Principal Investigator**: Dr. Mark Withers

Child’s Name: _________________________________ Child’s Age: _________ months

The research study has been explained to me and I understand and agree to the following:

1. It is up to me to decide whether to allow my child to participate in this study, and I can withdraw my child at any time without fear of any penalty.
2. The study will take 12 months and my child and I need to be available during that time.
3. My child will receive 3 injections of either an experimental malaria vaccine or rabies vaccine. I will have to bring my child back to the clinic for evaluation at 1, 2, 3, 7 and 14 days after each injection.
4. My child may experience side effects such as soreness at the site of injection. Although very unlikely, more serious and life threatening side effects such as allergic reactions are possible, but medicine and equipment will be available for treatment at the clinic.
5. My child will have to visit the clinic 23 times during the study, and at times up to 1/2 teaspoon of blood will be obtained from my child.
6. A field worker will visit my home about once per month after the last dose of vaccine to make sure my child is doing well.
7. I have read and received a copy of the informed consent explanation and I have been given the opportunity to ask questions to my satisfaction.
8. At the end of the study, if my child received the malaria vaccine, he or she will also be permitted to receive the rabies vaccine series.
9. If I have any further questions, I may contact Dr. Mark Withers at 057-22942.

___________________

Thumbprint of subject’s parent or legal representative if unable to sign:

Signature of Person Giving the Consent Explanation Date

__________________________________________ __________________

Parent’s / Legal Representative’s Signature Date

__________________________________________

Parent’s / Legal Representative’s Printed Name

__________________________________________ __________________

Thumbprint of subject’s 2^nd^ parent or legal representative if unable to sign:

2^nd^ Parent’s / Legal Representative’s Signature Date

__________________________________________

2^nd^ Parent’s / Legal Representative’s Printed Name

__________________________________________________

__________________________________________________

__________________________________________________

Explanation, if only one parent’s signature is available (If applicable)

Compound / House Number: ___________

Village Name: _______________________

### **17.4.4 Consent Form for Future Research Use & Long-Term Blood Sample Storage**

I agree that the investigators may store blood samples of my child indefinitely for possible use in other research studies. I understand that no human genetic (DNA) studies will be undertaken with these samples. I also understand that, if such use is contemplated, the investigators will first seek the approval of the KEMRI Ethical Review Committee. I undestand that the decision to allow storage of blood samples is not optional.

___________________

Thumbprint of subject’s parent or legal representative if unable to sign:

Signature of Person Giving the Consent Explanation Date

__________________________________________ __________________

Parent’s / Legal Representative’s Signature Date

__________________________________________

Parent’s / Legal Representative’s Printed Name

__________________________________________ __________________

Thumbprint of subject’s 2^nd^ parent or legal representative if unable to sign:

2^nd^ Parent’s / Legal Representative’s Signature Date

__________________________________________

2^nd^ Parent’s / Legal Representative’s Printed Name

### **17.4.4 Identification Photograph Consent Form**

I agree that the investigators may take photographs of me and my child for purposes of reliable identification during this research study only. I also understand that, at study completion, all such identification photographs will remain strictly confidential.

___________________

Thumbprint of subject’s parent or legal representative if unable to sign:

Signature of Person Giving the Consent Explanation Date

__________________________________________ __________________

Parent’s / Legal Representative’s Signature Date

__________________________________________

Parent’s / Legal Representative’s Printed Name

__________________________________________ __________________

Thumbprint of subject’s 2^nd^ parent or legal representative if unable to sign:

2^nd^ Parent’s / Legal Representative’s Signature Date

__________________________________________

2^nd^ Parent’s / Legal Representative’s Printed Name

*Complete section below only if subject’s parent or legal representative is illiterate:*

**Witness to Consent Interview**

On the date given next to my signature, I witnessed the “Informed Consent Interview” for the Research Study named above in this document. I attest that the information in these consent forms were explained to the subject’s parent or the subject's legally authorized representative, and the subject’s parent or subject’s representative indicated that his/her questions and concerns were adequately addressed.

Signature of Witness _____________________________ Date ________________

Printed Name of Witness _____________________________________________

**Witness to Subject’s Signature**

On the date given next to my signature, I witnessed the subject’s parent or the subject’s legally authorized representative, sign his/her name or imprint his/her thumbprint(s) on the consent form(s).

Signature of Witness _____________________________ Date ________________

Printed Name of Witness ____________________________________________
